# Interaction Analysis of Adenovirus L5 Protein With Pancreatic Cancer Cell Surface Receptor to Analyze Its Affinity for Oncolytic Virus Therapy

**DOI:** 10.3389/fonc.2022.832277

**Published:** 2022-03-10

**Authors:** Maryum Nisar, Rehan Zafar Paracha, Alvina Gul, Iqra Arshad, Saima Ejaz, Didar Murad, Shahzeb Khan, Zartasha Mustansar

**Affiliations:** ^1^ Research Center for Modelling and Simulation (RCMS), National University of Sciences and Technology (NUST), Islamabad, Pakistan; ^2^ Atta-ur-Rahman School of Applied Biosciences (ASAB), National University of Sciences and Technology (NUST), Islamabad, Pakistan; ^3^ Department of Pharmacy, University of Malakand, Chakdara, Pakistan; ^4^ Discipline of Pharmaceutical Sciences, School of Health Sciences, University of KwaZulu-Natal, Durban, South Africa

**Keywords:** pancreatic cancer, oncolytic viruses, immunotherapy, overexpressed receptors, HADDOCK, adenovirus, binding affinity, interaction pattern

## Abstract

This study seeks to investigate the interaction profile of the L5 protein of oncolytic adenovirus with the overexpressed surface receptors of pancreatic cancer. This is an important area of research because pancreatic cancer is one of the most fatal malignancies with a very low patient survival rate. Multiple therapies to date to improve the survival rate are reported; however, they show a comparatively low success rate. Among them, oncolytic virus therapy is a type of immunotherapy that is currently under deliberation by researchers for multiple cancer types in various clinical trials. Talimogene laherparepvec (T-VEC) is the first oncolytic virus approved by the US Food and Drug Administration (FDA) for melanoma. The oncolytic virus not only kills cancer cells but also activates the anticancer immune response. Therefore, it is preferred over others to deal with aggressive pancreatic cancer. The efficacy of therapy primarily depends on how effectively the oncolytic virus enters and infects the cancer cell. Cell surface receptors and their interactions with virus coat proteins are a crucial step for oncolytic virus entry and a pivotal determinant. The L5 proteins of the virus coat are the first to interact with host cell surface receptors. Therefore, the objective of this study is to analyze the interaction profile of the L5 protein of oncolytic adenovirus with overexpressed surface receptors of pancreatic cancer. The L5 proteins of three adenovirus serotypes HAdV2, HAdV5, and HAdV3 were utilized in this study. Overexpressed pancreatic cancer receptors include SLC2A1, MET, IL1RAP, NPR3, GABRP, SLC6A6, and TMPRSS4. The protein structures of viral and cancer cell protein were docked using the High Ambiguity Driven protein–protein DOCKing (HADDOCK) server. The binding affinity and interaction profile of viral proteins against all the receptors were analyzed. Results suggest that the HAdV3 L5 protein shows better interaction as compared to HAdV2 and HAdV5 by elucidating high binding affinity with 4 receptors (NPR3, GABRP, SLC6A6, and TMPRSS4). The current study proposed that HAdV5 or HAdV2 virus pseudotyped with the L5 protein of HAdV3 can be able to effectively infect pancreatic cancer cells. Moreover, the current study surmises that the affinity maturation of HAdV3 L5 can enhance virus attachment with all the receptors of cancer cells.

## 1 Introduction

Pancreatic cancer is the most fatal and extremely aggressive malignancy with a high recurrence and mortality rate (1). Chemotherapies and other targeted therapies show insignificant clinical outcomes with very little effect on the overall patient survival rate. Pancreatic cancer cells have been reported with significant drug resistance even against the Food and Drug Administration (FDA)-approved chemotherapies such as gemcitabine and FOLFIRINOX (2, 3). The 5-year survival rate of pancreatic cancer patients increases from 6% to 9% only, after extensive attempts from the year 2011 to 2020 for developing efficient therapy (4). Owing to the high tendency of developing resistance against chemotherapies, it has obtained noticeable attention to develop advanced therapies for pancreatic cancer (3). Patients usually develop adverse health conditions after chemotherapy like gastrointestinal (GI) toxicities, GI ulceration, and perforation (5). The aggressive and chemoresistant nature of pancreatic cancer encourages researchers to explore conventional therapies, targeted therapies, and immunotherapies. The targeted therapy involves the prediction of disease-related biomarkers and the development of therapy targeting the predicted biomarker (5). Various genes, e.g., BRCA1, BRCA2, PALB2, and CDKN2A, are tumor suppressor genes, but mutations in these genes escalate the risk of pancreatic cancer development (6). However, due to advancements in the management and treatment of cancer, the target therapies are under consideration for these mutated genes like NTRK gene fusions against BRCA mutations (7). Immunotherapies imply the inoculation of an immune component to the patient’s body for activation of the immune response against cancer. Some immunotherapies under consideration in multiple clinical trials include immunomodulators (i.e., immune checkpoint inhibitors, immune-stimulatory agonists, cytokines, and adjuvants), cancer vaccines, oncolytic virus therapy, and adoptive cell therapies (i.e., T-cell therapy and natural killer cells) (8).

Among a range of cancer therapies, oncolytic virus therapy is a novel targeted immunotherapy and has obtained noticeable attention. Viruses selectively exterminate cancer cells through lysis resulting in antitumor immune simulation. The specificity and effectiveness of oncolytic viruses make them an attractive therapeutic approach. However, success lies under the goal to modify (genetically engineer) viruses for enhanced tumor selectively and immune stimulation. Furthermore, the co-administration of oncolytic viruses with other therapeutic agents has also been found to be very effective (9). The very first oncolytic virus sanctioned by the FDA is talimogene laherparepvec (T-VEC), which is a genetically altered herpes simplex virus type 1 (HSV1) used to treat melanoma (10, 11). It is imperative to study the infection mechanism of viruses in cancer cells for enhancing the tumor selectivity of oncolytic viruses. The entrance of oncolytic viruses into the cancer cell is the first step of oncolytic virus therapy. It is completely dependent on the binding of oncolytic viruses with the surface exposed cellular receptors, resulting in the receptor’s mediated viral endocytosis and activation of pathways that mediate viral entry. Therefore, it is paramount to characterize the cancer cell receptor’s interaction dynamics with the virus receptor.

Human adenovirus is a commonly used oncolytic virus against various cancer types. Gendicine and Oncorine are the two adenoviruses that are currently approved for the treatment of cancer in China (3). ONXY-15 is the first oncolytic virus used in a clinical trial for pancreatic cancer (12). The mode of entry of adenovirus is based on first the interaction of viral coat proteins [fiber knob (L5) and penton (L2)] with human cell receptors, and then the endocytosis of viral particle occurs. First, the L5 protein interacts with human cell receptors, followed by penton protein binding with integrin subunits. The human adenovirus C serotype 2 (HAdV2) and C serotype 5 (HAdV5) are mostly used as oncolytic viruses. The L5 protein of both serotypes interacts with the CAR receptor for virus entry in the cell. A previous study determined that the HAdV3 fiber knob, which interacts with Desmoglein 2 (DSG2), can enhance the oncolytic properties of HAdV2 and HAdV5 (13, 14). The membrane cofactor protein CD46 is also reported as a receptor that interacts with the HAdV3 L5 protein. Some studies suggest that HAdV3 does not show a high binding affinity with CD46 (15). The complement receptor (CR1) and CAR expressed on the erythrocyte membrane interact with the HAdV5 L5 protein and hinder the adenovirus infection (16). In this study, the L5 proteins of 3 serotypes HAdV2, HAdV3, and HAdV5 are analyzed for their interaction pattern with pancreatic cancer receptors. The L5 protein of HAdV2, HAdV3, and HAdV5 are homo-trimeric proteins that consist of multiple regions with the length of 582, 319, and 581 amino acids, respectively. These regions for HAdV2 and HAdV3 include an amino-terminal penton base attachment domain of 44 amino acid length and a long thin central shaft region of 348 amino acids, and the knob domain of HAdV2 is 184 amino acids and 183 for HAdV5 (17). The HAdV3 has a small shaft region of 85 amino acids, and the knob domain is 191 amino acid large (18).

Adenovirus cell attachment is instigated by the interaction of the knob domain region of the fiber protein with the human cell receptors (19). The CAR and DSG2 are cell–cell adhesion molecules, which are underexpressed in multiple cancer types due to the disrupted Raf-MEK-ERK pathway. The Raf-MEK-ERK pathway is overexpressed in pancreatic cancer, suppressing the expression of CAR and DSG2 (20, 21). The proteome analysis of pancreatic cancer cell lines detected CD46, while no clear correlation was observed between the cell line mRNA data for CD46 with the mass spectrometry (MS) data or fluorescence-activated cell sorting (FACS) data. For CD46, no significant increase was observed at the mRNA level for all cell lines tested (22). Lee et al. (22) also tested CD46 overexpression in 10 pancreatic cancer tissue samples. Immunohistochemistry results for CD46 show that staining in 2 of 10 tissues was observed. This study aimed to investigate the interaction pattern of the L5 proteins with multiple pancreatic cancer cell receptors studied. The seven overexpressed genes in the pancreatic cancer cells, which were identified in one of our previous studies, are further analyzed in the prospect of oncolytic virotherapy. In that previous study, integrated transcriptomic analysis of 5 microarray and 2 RNA-seq pancreatic cancer datasets was performed. The datasets analyzed were comprised of pancreatic cancer patient tissue samples. In our previous study, we selected the genes that were highly overexpressed (*log*_2_ *FC* > 1) in all the datasets and also identified in The Cancer Genome Atlas (TCGA) cohort. These genes include MET, NPR3, IL1RAP, SLC2A1, SLC6A6, GABRP, and TMPRSS4, which encode for cellular membrane receptors (23). The overexpression of these receptors in pancreatic cancer is also verified from the literature (24–27).

SLC2A1 gene encodes for glucose transporter type 1 (Glut1), which is a member of the GLUT protein family. It is responsible for glucose transport across the cell membrane. The structure of SLC2A1 comprises 12 transmembrane helices, with 6 extracellular and 7 cytoplasmic domains (28, 29). Carcinogenesis requires an excessive amount of energy, which is harnessed by increased glucose metabolism. The elevated level of Glut1 expression is reported in various cancer types such as pancreatic, breast, endometrial, ovarian, and cervical cancers (30). In pancreatic cancer, the upregulation of Glut1 induces tumor progression by accumulating glycogen and activating various neoplastic pathways in cells (24, 31). The findings from the recently published literature were explored to identify the interaction of Glut1 with any virus. The interaction of Glut1 with the envelope glycoproteins of the human T-cell leukemia virus (HTLV) facilitates the viral entry in the cell (32). Taking this evidence as a precursor (antecedent) for our study, we explored the interaction of adenovirus fiber knot protein with Glut1.

MET gene encodes for a c-Met receptor tyrosine kinase, a large protein with an approximate molecular weight of 190 kDa (33). MET gene decodes in a single chain of 1,390 amino acids and then transforms into 50-kDa extracellular *α* chain and transmembrane 140-kDa *β* chains (34). Met regulates multiple signaling pathways such as Ras, PI3K, JAK/STAT, actin deposition, and *β* -catenin (Wnt) pathways. These signaling pathways cause cell proliferation, evading apoptosis and leading to the development of angiogenesis and invasiveness (35, 36). Met receptor tyrosine kinase is the most attractive oncogenic therapeutic target for multiple malignancies (36). Met overexpression in pancreatic cancer is studied in various previous studies (26, 35), so this signifies its importance as a therapeutic target.

Atrial natriuretic peptide receptor 3 (NPR3) also known as NPR-C is a member of the glycosylated receptor family. This is present in the form of a dimer, and it is responsible for the regulation of natriuretic peptide (NP) hormones in the blood (37). Three NP hormones include atrial natriuretic peptide (ANP), B-type natriuretic peptide (BNP), and C-type natriuretic peptide (CNP). The ANP regulates blood pressure by managing the excretion of sodium and water (38). The NPR-C structure comprises the large extracellular domain (455 amino acids) attached with a helical transmembrane region of 23 amino acids (37). IL-1 receptor accessory protein (IL-1RAcP) is the co-receptor of type I IL-1 receptor (IL-1RI). IL-1R1 is a plasma membrane receptor, which initiates multiple signaling pathways by activating cytoplasmic kinases. IL-1RAcP is crucial for the binding of IL-1 with IL-1RI (39). The IL-1RAcP has a molecular mass of 66 kDa and consists of 570 residues with 347 residues of the long extracellular domain, 21 residues of transmembrane helices, and 182 residues of the long cytoplasmic domain (40).

SLC6A6 encodes for multipass membrane transport protein. It is a member of the solute carrier 6 (SLC6) family of sodium and chloride ion-dependent transporter proteins (41, 42). These proteins are involved in neurotransmission, amino acids, monoamines, osmolytes, GABA, and energy metabolite transport. SLC6A6, also known as TauT, is categorized as GABA transporter and transport taurine (41). Taurine is an essential amino acid and helps in the normal structure development of cells by inducing an anti-apoptotic response. The absence of this amino acid in the cell leads to anatomical structure deformation, while abundance leads to persistent activation of anti-apoptotic pathways. The elevated level of SLC6A6 is reported in multiple malignancies like colorectal, cervical, and gastric cancers (42). The overexpression of SLC6A6 in pancreatic cancer is determined in multiple gene expression-based studies (25, 27, 43). Transmembrane protease serine 4 (TMPRSS4) is a member of the transmembrane serine protease family. TMPRSS4 protein comprises 437 amino acids with a large extracellular proteolytic domain and 48-kDa molecular weight (44). The main function of this protein includes embryo development and cancer progression. In addition, it is responsible for inducing cell invasion, cell proliferation, motility, and cell-matrix adhesion (44, 45). TMPRSS4 shows high clinical potential in various malignancies including pancreatic cancer and is under consideration as a novel oncogenic therapeutic target (44–46).

All the above-enlisted receptors have a direct or indirect link with the progression of pancreatic cancer. In this study first, the structures of all receptors are retrieved. For SLC2A1, MET, IL1RAP, and NPR3 receptors, Protein Data Bank (PDB) structures are available, while for other receptors, machine learning-based structure prediction is utilized to generate the protein structure. The structures of the L5 proteins for all three serotypes are downloaded from PDB. All the receptors are docked with the HAdV2, HAdV3, and HAdV5 L5 proteins. The binding affinity and interaction pattern of each complex are analyzed to identify which target receptor effectively binds with the L5 proteins. For the positive control, the interaction pattern of CAR and DSG2 with the HAdV2, HAdV3, and HAdV5 L5 proteins is analyzed and compared with pancreatic cancer receptors. The interaction analysis of CR1 with the HAdV5 L5 protein and CD46 with the HAdV3 L5 protein is also performed to compare the binding affinity of these receptors with pancreatic cancer receptors. NPR3, GABRP, SLC6A6, and TMPRSS4 showed the highest binding affinity with the HAdV3 L5 protein, while SLC2A1 and IL1RAP show better interaction with HAdV2.

## 2 Materials and Methods

**Figure 1:**
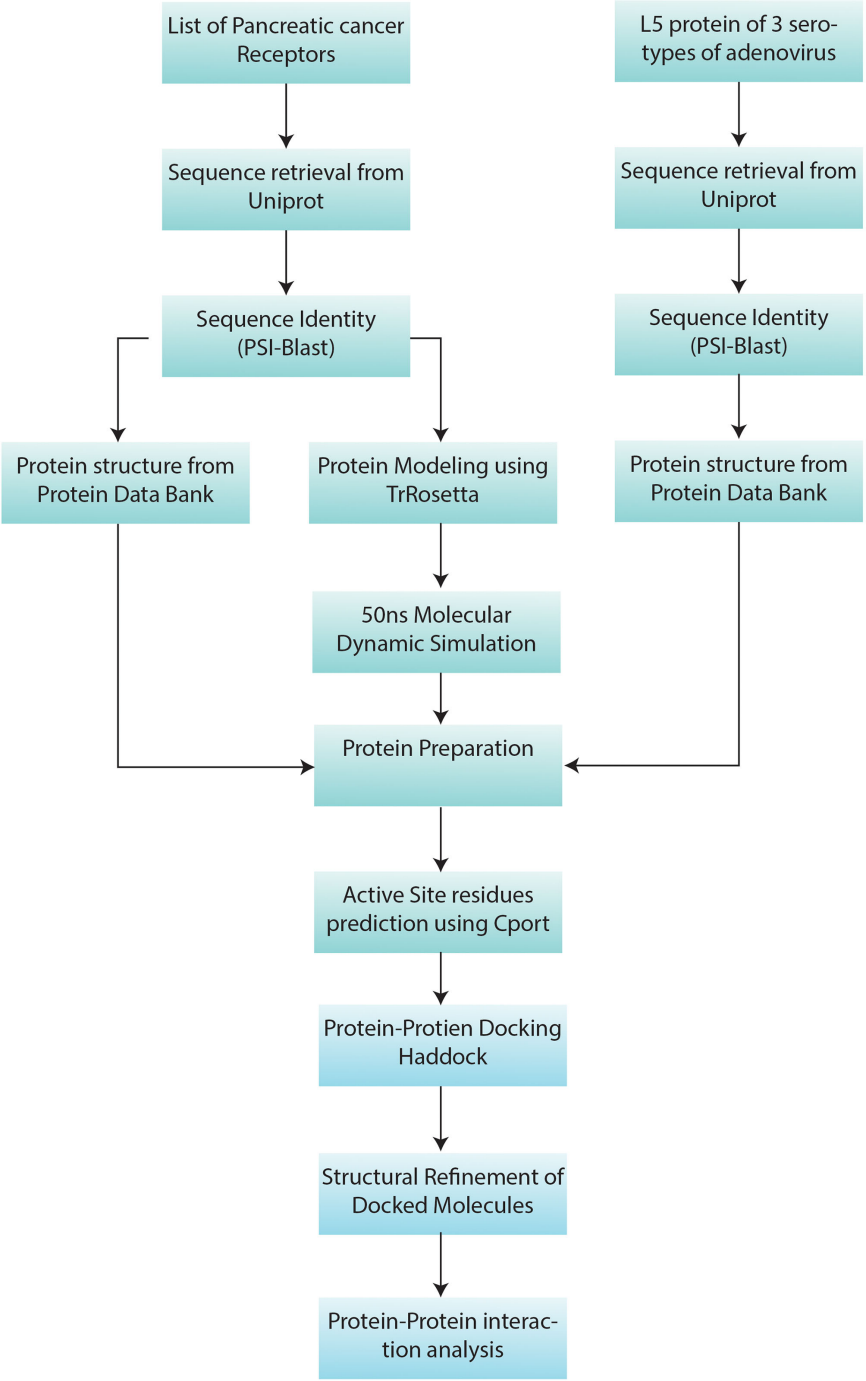
Methodology workflow: the sequence and structure information of 7 pancreatic cancer receptors (**Table 1**) and L5 proteins of HAdV2, HAdV3, and HAdV5 (**Table 2**) were retrieved from UniProt. Then PSI-Blast was performed. Protein Data Bank (PDB) structures of proteins with available structures were downloaded from the PDB database. Machine learning-based structure prediction was performed for proteins with a sequence identity of less than 60%. The 50-ns molecular dynamic (MD) simulation was performed to refine the predicted models. Then pancreatic cancer receptors and HAdV L5 proteins were docked and refined. Then interaction analysis of both interacting molecules was performed.

The overall workflow of this study includes structure retrieval and preparation of human receptors and virus L5 protein. Then the docking of receptors with L5 and interaction analysis of both molecules was performed. The overall workflow of the study is given in **Figure 1**.

### 2.1 Structure Modeling of Human Receptors

The sequences of receptors were retrieved from UniProt (https://www.uniprot.org/) against each UniProt ID provided in **Table 1**. The PSI-Blast (https://blast.ncbi.nlm.nih.gov/Blast.cgi) was performed to determine percentage sequence identity based on which structural homologs were identified. The MET, SLC2Al, NPR3, IL1RAP, CAR, DSG2, CD46, and CR1% identity was greater than 97%, which means that the protein structure for these receptors was already available. For the SLC2A1 receptor, multiple PDB structures with a similarity >99% were identified with a sequence length of 492 amino acids. Out of all hits, a structure with high resolution (2.4 Å) with PDB ID 6THA was selected and downloaded from PDB (https://www.rcsb.org/). The PDB ID 1KY0 was selected for the NPR3 receptor based on the PSI-Blast result. The 1KY0 structure of the NPR3 extracellular domain is present with 88% sequence coverage and a high-resolution (2.40 Å) X-ray diffraction structure. The MET receptor comprises a large extracellular domain, a transmembrane region, and an intracellular domain with a sequence length of 1,390 amino acids. The extracellular domain comprises 25 to 932 amino acid residues. Two PDB structures with similarity >99% and 57% sequence coverage were identified through PSI-Blast. The PDB structures 2UZX (2.80 Å) and 6GCU (6.00 Å) are further analyzed. Both cover the extracellular domain region but have a missing loop region. To model the missing loop regions for MET, a homology protein model was generated using Swiss-Model (https://swissmodel.expasy.org/), using 2UZX as the PDB template.

**Table 1:** Pancreatic cancer receptors.

Sr. no.	Protein receptor name	UniProt ID	Sequence length	% identity	PDB ID	Sequence coverage	PDB model used
1	MET proto-oncogene, receptor tyrosine kinase (MET)	P08581	1,390	100%	2UZX	57%	Homology model of 2UZX
2	Solute carrier family 2 member 1 (SLC2A1)	P11166	492	100%	6THA	100%	6THA
3	Natriuretic peptide receptor 3 (NPR3)	P17342	541	100%	1YK0	88%	Homology model of 1YK0
4	Interleukin 1 receptor accessory protein (IL1RAP)	Q9NPH3	570	100%	4DEP	61%	Homology model of 4DEP
5	Gamma-aminobutyric acid type A receptor pi subunit (GABRP)	O00591	440	48.34%	4COF	67%	TrRosetta model
6	Solute carrier family 6 member 6 (SLC6A6)	P31641	620	47.34%	4YPB	87%	TrRosetta model
7	Transmembrane protease, serine 4 (TMPRSS4)	Q9NRS4	437	37.99%	7MEQ	82%	TrRosetta model
Positive control							
1	Coxsackievirus and adenovirus receptor (CAR/CXADR)	P78310	365	100%	1EAJ	34%	1EAJ
2	Desmoglein 2 (DSG2)	Q14126	1,118	99.82%	5ERD	49%	5ERD
3	Membrane cofactor protein (CD46)	P15529	392	100.00%	3O8E	64%	3O8E
4	Complement receptor type 1 (CR1)	P17927	2,039	97.60%	2MCZ	128 residues	2MCZ
				100.00%	5FO9	196 residues	4FO9

Protein receptor name and UniProt ID, followed by sequence length and % sequence identity with known Protein Data Bank (PDB) structures/PSI-Blast hit and then followed by PDB ID and sequence coverage. The last column indicates the information of the model used for the analysis.

For CAR PDB structure, 1EAJ (1.35 Å) was selected, with 100% percentage identity and 34% sequence coverage identified through PSI-Blast. The selected PDB structure comprises amino-terminal immunoglobulin domain (D1). This D1 domain is necessary and sufficient for HAdV binding with CAR (47). For the DSG2 PDB structure, 5ERD (2.90 Å) was selected. The structure comprises the extracellular domain of DSG2, with sequence coverage of 49% and 99.82% percentage identity. For the CD46 PDB structure, 3O8E (2.84 Å) was selected, having sequence coverage of 64% and 100% identity. The CR1 extracellular domain comprises 30 sushi domains. For CR1, two PDB structures 2MCZ and 5FO9 (3.30 Å) were selected for further analysis. The 2MCZ PDB structure consists of 1 and 2 sushi domains and 5FO9 15, 16, and 17 sushi domains. No significant structural homologs were identified for GABRP, SLC6A6, and TMPRSS4. The sequence similarity for these three receptors was less than 60% and greater than 30%, so threading-based structure prediction was performed. The protein sequences from UniProt were submitted to three threading servers I-TASSER (https://zhanglab.ccmb.med.umich.edu/I-TASSER/) (48), RaptorX (http://raptorx.uchicago.edu/) (49), and machine learning-based structure prediction server TrRosetta (https://robetta.bakerlab.org/) (50). TrRosetta was developed on CASP advances in predicting distance distribution for scoring structure models. TrRosetta modeled the structures by presenting inter-residue orientations and efficient energy minimization-based structure realization of Rosetta. The TrRosetta deep neural network is on millions of known sequences and structures (51). Then the structure models of these servers were validated using ERRAT, QMean, Verify 3D, ProCheck, and QMeanBrane. The results of TrRosetta were better than those of other servers and thus used for the rest of the analysis.

Molecular dynamic (MD) simulation of selected models of TrRosetta server for proteins was performed. GROMACS (GROningen MAchine for Chemical Simulations) was used for energy minimization and MD simulation of all proteins (52). The OPLS-AA (Optimized potential for Liquid simulation) force field was used for writing the topology of protein molecules (53). For the solvation step, a cubic box of water molecules was used. Protein was placed in the center of the box with 1.0-nm distance from the box edge and 2.0-nm distance periodic image of the protein. The 3-point solvent model spc216.gro for equilibrating protein was used. For removing the charges on protein, GROMACS used the genion tool. Then energy minimization was performed, and minimized structures with the least steric clashes were obtained. Then simulations for 50,000 steps were performed for NVT equilibration. The temperature of the system was equilibrated to 300K. Then the pressure and the density of the system were equilibrated; a 50,000-step simulation was performed for NPT equilibration. Then equilibrated proteins were simulated for 50 ns (50,000 ps) (25,000,000 steps). Root mean square deviation (RMSD) for protein backbone energy minimization from the initial step to the final step of MD is explored. To determine protein compactness throughout MD simulation, the radius of gyration was analyzed. Xmgrace tool was used for analyzing and generating the plots of energy minimization, RMSD, and the radius of gyration (54).

### 2.2 Adenovirus Proteins

The sequences of the L5 protein for all serotypes were retrieved from UniProt (https://www.uniprot.org/) against each UniProt ID provided in **Table 2**. The PSI-Blast (https://blast.ncbi.nlm.nih.gov/Blast.cgi) was performed to determine the percentage sequence identity basis on which PDB structures were determined. The percentage identity and sequence coverage of selected PDB structures are provided in **Table 2**. The selected PDB structures were downloaded from PDB (https://www.rcsb.org/). For HAdV2 and HAdV3, high-resolution PDB structures IQIU (2.40 Å) and 1H7Z (1.60 Å) were selected. PDB structure for both serotypes comprises the head region and a small portion of the shaft region. For HAdV5, L5 4ATZ PDB was selected with a resolution of 1.95 Å and comprises the knob region. Although the sequence coverage for all the L5 proteins was less, it was considered for this study because the structure of the knob region is required to analyze its interaction pattern with pancreatic cancer receptors.

**Table 2:** Adenovirus serotypes L5 protein.

Sr. no.	Serotype name	UniProt ID	Sequence length	% identity	PDB ID	Sequence coverage
1	Human adenovirus C serotype 2 (HAdV2) Fiber protein (L5)	P03275	582	100%	1QIU	45%
2	Human adenovirus C serotype 5 (HAdV5) Fiber protein (L5)	P11818	581	97.45%	4ATZ	33%
2	Human adenovirus B serotype 3 (HAdV3) Fiber protein (L5)	P04501	319	99.4%	1H7Z	61%

Serotype name, UniProt ID, sequence length, % sequence identity with known Protein Data Bank (PDB) structures/PSI-Blast hit, and PDB Ids. The last column sequence coverage.

### 2.3 Structure Preparation for Docking

Then receptor molecules were prepared in ProteinPrepare module of Play Molecule by removing water molecules, and residues were optimized regarding protonation state with respect to selected titration state on pH 8.0 (55). The pancreatic juice has a pH of 8.0–8.3, so for this analysis, pH 8.0 was used (56). Then prepared molecules were submitted in CPORT haddock server (https://alcazar.science.uu.nl/services/CPORT/) to determine active site residues or protein interface residues (57). Consensus Prediction Of interface Residues in Transient complexes (CPORT) was the combination of six interface prediction servers (WHISCY, PIER, ProMate, cons-PPISP, SPPIDER, and PINUP). It generates a consensus score from all these servers to accurately predict the residues (57). The active site surface residues of the HAdV L5 proteins, pancreatic cancer receptors, and positive control receptors are given in **Tables S1–S3**, respectively.

### 2.4 Docking Run

Protonated structures of receptors and L5 proteins were submitted to HADDOCK 2.4 (https://wenmr.science.uu.nl/haddock2.4/) server for protein–protein docking of both molecules (58). The GURU interface of High Ambiguity Driven protein–protein DOCKing (HADDOCK) was used for docking, and the list of active and passive side residues predicted by CPORT was given as input. HADDOCK was a flexible docking approach comprising three steps/stages: (*it*_0_) rigid body energy minimization, (*it*_1_) semi-flexible refinement in torsion angle space, and (*it*_w_) refinement in explicit solvent refinement. The HADDOCK scoring function is the linear combination of energies and buried surface area in all three stages of docking. Docking run through HADDOCK software is computationally extensive, so the server provides users with an easy-to-use interface and removes the burden of computational resources required for installation and docking run (58, 59). The HADDOCK server generates clusters of similar conformations for interacting molecules.

### 2.5 Binding Affinity and Interaction Analysis

The interaction model from the top HADDOCK cluster was refined to remove structural restraints using HADDOCK 2.4 Refinement (https://wenmr.science.uu.nl/haddock2.4/refinement/1). After that, binding affinity (ΔG) and dissociation constant (*K_d_*) of the protein–protein complex were calculated using PRODIGY (PROtein binDIng enerGY prediction) (https://wenmr.science.uu.nl/prodigy/) (60). Prodigy also calculates the number and type of intermolecular interactions within 5.5-Å distance cutoff (61). Other tools and software used include Pymol and Chimera.

## 3 Results

For the docking run, structures of receptors and L5 protein were prepared. For SLC2A1, MET, NPR3, and IL1RAP, receptors and L5 proteins with known PDB structures were employed, while structures of GABRP, TMPRSS4, and SLC6A6 were modeled.

### 3.1 Modelling of GABRP, TMPRSS4, and SLC6A6

The PDB structures of GABRP, TMPRSS4, and SLC6A6 proteins were not available. The PSI-Blast result for GABRP shows 48.34% identity, SLC6A6 47.34%, and TMPRSS4 37.99% (**Table 1**). All three proteins show identity levels between 60% and 30%, so threading-based and machine learning-based structure predictions were used for model generation. The protein model produced from TrRosetta webserver was selected for further analysis. Then MD simulation of 50 ns was performed in GROMACS to simulate protein for the best possible conformation in water. The resultant plots of MD simulations are provided in **Figures S1–3**. The MD plots include equilibration phase graphs: pressure (a), density (c), and temperature (d). In the equilibration phase, protein system pressure, density, and temperature are stabilized before the MD run.

**Figures S1B**, **S2B** represent the potential energy minimization plots for GABRP and SLC6A6, which are proteins that reached their minimized state on 2,000 ps. **Figure S3B** represents the potential energy minimization plots for TMPRSS4. Energy minimization of protein is performed to remove any steric clashes in structure and structure relaxed in a solvent medium. **Figure S1E** shows the RMSD plot of GABRP RMSD value of approximately 0.6 nm on 50 ns. **Figures S2E**, **S3E** represent the RMSD plots for SLC6A6 and TMPRSS4, with RMSD values of approximately 0.5 and 1.5, respectively. The radius of gyration is used to scale the amount of protein compactness (folding state). If the protein is stably folded, then it shows relatively steady values of radius of gyration. **Figures S1F, S2F**, **S3F** represent the relatively invariable radius of gyration values with respect to the time change, on a temperature of 300K. The compactness of GABRP stabilizes approximately 3.7 to 3.9 Rg(nm) from time steps 18,000 to 50,000 ps (**Figure S1F**). The compactness of SLC6A6 stabilizes approximately 2.7 to 2.75Rg(nm) from time steps 20,000 to 50,000 ps. The compactness of TMPRSS4 stabilizes approximately 2.4 to 2.6 Rg(nm) from time steps 15,000 to 50,000 ps. This represents that all proteins remain very stable and remain in their folded form.

### 3.2 Protein–Protein Interaction of HAdV2 L5 Protein With Receptors

#### 3.2.1 Pancreatic Cancer Receptors

**Figure 2:**
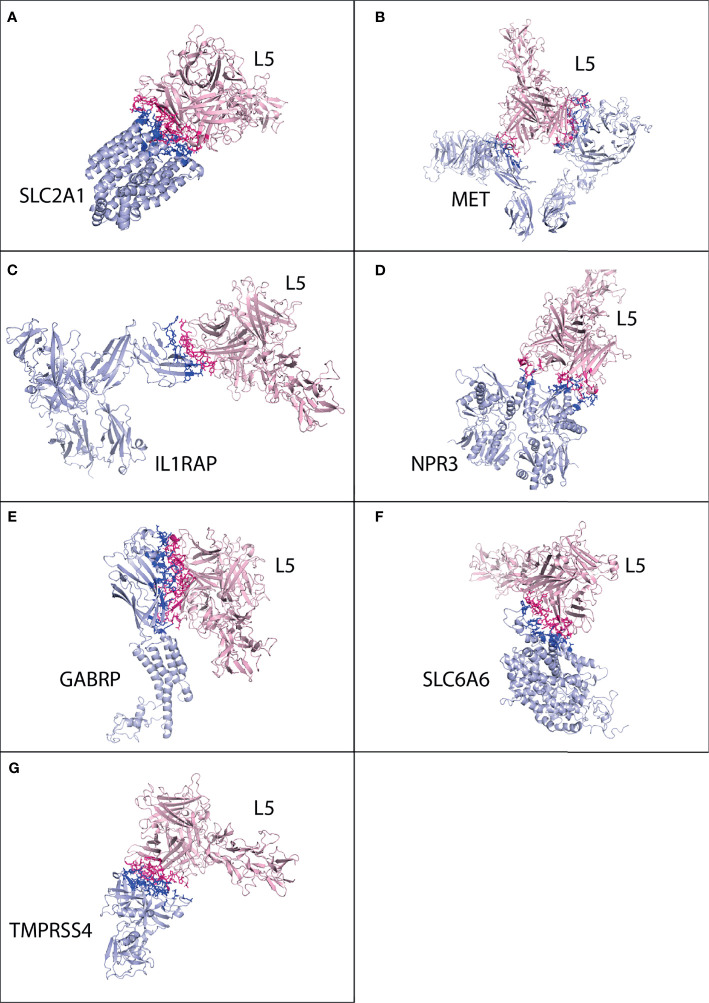
Protein–protein interaction complex of HAdV2 L5 protein with pancreatic cancer receptors. The pink ribbon represents L5 protein, while purple ribbons represent cancer receptors. The interacting residues are shown by a stick, L5 protein is shown in dark pink, and receptor is shown in dark blue. **(A)** SLC2A1 made a total of 141 interactions. **(B)** MET generated 76 interactions. **(C)** IL1RAP made 75 interactions. **(D)** NPR3 made 78 interactions. **(E)** GABRP made a total of 169 contacts. **(F)** SLC6A6 made 84 interactions. **(G)** TMPRSS4 made 119 interactions.

The protonated structures of pancreatic cancer receptors were docked with the protonated HAdV2 L5 protein using the GURU interface of the HADDOCK server. The HADDOCK generates water-refined interaction conformations of both interacting molecules and groups similar conformations in clusters. The superimposed structures of all structures in one cluster were generated (**Figures S7**–**S13**), along with the plots of HADDOCK scores, Cluster size, RMSD, Van der Waals energy, electrostatic energy, desolvation energy, restraint violation energy, buried surface area, and Z-score. The best possible interaction complex for all receptors with the best HADDOCK score and lowest Z-score are then selected for refinement. The interacting complexes were refined for the orientation of interacting molecules using the Refinement module of HADDOCK. The statistical parameter values for refined complexes are provided in **Table 3**. The complexes were further analyzed in Prodigy for the number and types of interactions between the HAdV2 L5 protein and receptors. Prodigy generates a binding affinity value (ΔG) and a total number of interactions between both molecules. Also, the information of the total number of charged-charged interactions, charged–polar, charged–apolar, polar–polar, polar–apolar, and apolar–apolar interactions was generated in the analysis provided in **Table 4**. The cartoon representation of the refined structure and interaction complexes generated by Prodigy is provided in **Figure 2**.

**Table 3:** HADDOCK parameters after refinement.

Sr. no.	Interacting proteins	HADDOCK score	Cluster size	RMSD	Van der Waals energy	Electrostatic energy	Desolvation energy	Restraints violation energy	Buried surface area
HAdV2 L5									
1	SLC2A1	−182.2 ± 1.5	20	0.5 ± 0.3	−121.6 ± 2.8	−309.7 ± 20.4	1.4 ± 1.8	0.0 ± 0.0	3,278.8 ± 40.1
2	MET	−111.8 ± 2.5	20	0.6 ± 0.3	−56.6 ± 6.6	−308.2 ± 25.2	9.3 ± 4.1	0.9 ± 0.3	2,151.4 ± 129.7
3	IL1RAP	−111.7 ± 3.3	20	0.5 ± 0.3	−54.6 ± 4.3	−276.3 ± 8.0	−1.9 ± 2.0	0.9 ± 0.8	1,523.7 ± 45.6
4	NPR3	−102.4 ± 1.1	20	0.5 ± 0.3	−70.4 ± 4.7	−207.1 ± 21.4	9.4 ± 1.0	0.5 ± 0.3	2,098.0 ± 58.8
5	GABRP	−227.8 ± 1.3	20	0.5 ± 0.3	−133.8 ± 1.8	−393.5 ± 19.5	−15.3 ± 2.5	0.0 ± 0.0	3,983.4 ± 40.4
6	SLC6A6	−146.0 ± 3.1	10	0.6 ± 0.3	−83.9 ± 2.4	−144.6 ± 8.1	−33.3 ± 3.0	0.7 ± 0.3	2,255.2 ± 65.5
7	TMPRSS4	−176.2 ± 1.6	10	0.5 ± 0.3	−93.8 ± 3.5	−380.7 ± 25.3	−6.3 ± 5.4	0.4 ± 0.1	2,770.5 ± 61.1
1	CAR	−104.3 ± 3.3	20	0.5 ± 0.3	−49.3 ± 3.0	−277.1 ± 43.1	0.4 ± 3.4	0.7 ± 0.2	1,722.2 ± 31.1
2	DSG2	−141.9 ± 3.3	20	0.5 ± 0.3	−59.6 ± 3.3	−404.4 ± 24.5	−1.4 ± 1.1	0.2 ± 0.1	2,084.2 ± 77.5
HAdV3 L5									
1	SLC2A1	−164.2 ± 1.9	10	0.5 ± 0.3	−94.8 ± 4.7	−450.8 ± 23.3	20.7 ± 2.7	0.6 ± 0.4	2,949.9 ± 100.2
2	MET	−115.6 ± 0.6	20	0.6 ± 0.4	−65.8 ± 1.4	−186.1 ± 8.9	−12.6 ± 2.6	0.2 ± 0.1	1,985.7 ± 41.3
3	IL1RAP	−84.7 ± 0.9	20	0.5 ± 0.3	−49.7 ± 1.0	−132.1 ± 9.3	−8.7 ± 1.6	0.8 ± 0.3	1,796.0 ± 23.1
4	NPR3	−126.8 ± 3.0	20	0.5 ± 0.3	−70.3 ± 1.1	−357.4 ± 19.7	14.9 ± 0.8	1.2 ± 0.4	2,221.1 ± 27.8
5	GABRP	−241.0 ± 5.1	20	0.5 ± 0.3	−159.0 ± 1.4	−323.2 ± 22.7	−17.5 ± 5.5	0.8 ± 0.3	4,318.4 ± 45.8
6	SLC6A6	−242.1 ± 1.5	20	0.6 ± 0.3	−138.5 ± 3.9	−382.4 ± 21.5	−27.1 ± 1.0	0.0 ± 0.0	3,628.4 ± 42.5
7	TMPRSS4	−200.6 ± 4.6	10	0.5 ± 0.3	−105.0 ± 4.2	−538.9 ± 19.6	12.1 ± 3.3	0.7 ± 0.4	3,212.8 ± 47.6
1	CAR	−168.4 ± 5.5	20	0.5 ± 0.3	−89.6 ± 2.1	−394.1 ± 24.3	−0.1 ± 2.3	0.7 ± 0.2	2,879.1 ± 82.2
2	DSG2	−171.7 ± 3.5	20	0.6 ± 0.3	−82.8 ± 3.4	−483.4 ± 1.8	7.6 ± 2.6	0.9 ± 0.5	2,780.1 ± 92.3
3	CD46	−170.7 ± 5.0	20	0.5 ± 0.3	−97.0 ± 2.6	−326.7 ± 34.8	−8.5 ± 3.2	1.2 ± 0.6	2,927.2 ± 59.2
HAdV5 L5 protein									
1	SLC2A1	−191.0 ± 4.3	10	0.5 ± 0.3	−112.9 ± 2.8	−432.2 ± 19.4	8.3 ± 2.5	0.7 ± 0.3	3,246.7 ± 11.3
2	MET	−129.0 ± 3.5	20	0.5 ± 0.3	−75.0 ± 2.6	−279.7 ± 14.0	1.8 ± 1.6	1.1 ± 0.5	2,509.0 ± 85.0
3	IL1RAP	−80.9 ± 1.4	20	0.5 ± 0.3	−34.3 ± 1.7	−204.3 ± 26.4	−5.9 ± 5.0	0.8 ± 0.6	1,279.8 ± 40.1
4	NPR3	−76.7 ± 1.0	20	0.5 ± 0.3	−49.2 ± 1.3	−136.5 ± 8.7	−0.3 ± 1.9	0.4 ± 0.3	1,360.9 ± 21.5
5	GABRP	−143.2 ± 5.0	20	0.5 ± 0.3	−105.6 ± 3.8	−139.9 ± 20.6	−9.7 ± 1.6	0.6 ± 0.5	2,980.7 ± 42.9
6	SLC6A6	−208.7 ± 6.3	10	0.6 ± 0.3	−114.8 ± 1.1	−395.2 ± 23.3	−14.9 ± 2.7	0.8 ± 0.3	2,911.2 ± 67.0
7	TMPRSS4	−148.9 ± 0.1	10	0.5 ± 0.3	−66.9 ± 4.7	−454.9 ± 20.8	8.8 ± 2.8	1.4 ± 0.7	2,461.4 ± 41.7
1	CAR	−89.0 ± 1.9	20	0.5 ± 0.3	−51.5 ± 1.2	−225.5 ± 18.2	7.5 ± 1.2	1.0 ± 0.6	1,483.2 ± 37.0
2	DSG2	−122.2 ± 2.3	20	0.5 ± 0.3	−82.0 ± 2.7	−186.3 ± 13.9	−3.0 ± 2.6	0.8 ± 0.6	2,075.7 ± 68.2
3	CR1(2MCZ)	−156.2 ± 0.9	20	0.5 ± 0.3	−74.8 ± 2.9	−348.3 ± 19.4	−11.8 ± 3.3	0.8 ± 0.7	2,380.6 ± 39.0
4	CR1(5FO9)	−128.2 ± 1.2	20	0.5 ± 0.3	−69.1 ± 1.3	−294.2 ± 5.0	−0.3 ± 0.9	0.7 ± 0.2	2,171.6 ± 32.9

The table represents the information of refined clusters for all pancreatic cancer receptors, CAR, DSG2, CD46, and CR1 against HAdv2, HAdV3, and HAdV5 L5 proteins. The HADDOCK score is protein–protein docking score, cluster size is the number of similar interaction conformations generated and clustered together by HADDOCK. RMSD (root mean square deviation) conveys how much deviation of protein structure occurs from its original conformation. Van der Waals energy is a result of comparatively weak electric forces. Electrostatic energy refers to electromagnetic force that occurs when a molecule is bearing a static electrical charge. Desolvation energy is required for the interacting proteins to substitute the water molecules on the binding surface. The whole calculations for each structural restraint constitute the restraints violation energy, and it is supposed to be close to zero.

**Table 4:** Prodigy results.

Sr. no.	Interacting proteins	ΔG (kcal mol^−1^)	Kd (M) at 37.0°C	No. of total ICs	ICs charged–charged	ICs charged–polar	ICs charged–apolar	ICs polar–polar	ICs polar–apolar	ICsapolar–apolar
HAdV2 L5 protein										
1	SLC2A1	−16.0	5.4e−12	141	16	26	23	16	45	15
2	MET	−7.6	4.1e−06	76	11	31	14	12	6	2
3	IL1RAP	−11.5	7.3e−09	75	9	19	15	7	20	5
4	NPR3	−10.4	5.0e−08	78	10	19	9	7	18	15
5	GABRP	−17.8	3.0e−13	169	11	28	40	19	46	25
6	SLC6A6	−11.6	6.7e−09	84	10	10	21	11	24	8
7	TMPRSS4	−15.5	1.1e−11	119	18	23	29	8	29	12
1	CAR	−9.4	2.3e−07	63	7	14	12	10	13	7
2	DSG2	−12.7	1.1e−09	86	7	20	13	8	29	9
HAdV3 L5 protein										
1	SLC2A1	−13.7	2.3e−10	118	8	20	28	11	35	16
2	MET	−9.6	1.6e−07	73	6	14	15	10	16	12
3	IL1RAP	−9.9	1.1e−07	62	4	14	13	4	16	11
4	NPR3	−13.1	5.9e−10	92	7	15	19	2	25	24
5	GABRP	−21.9	3.4e−16	168	5	20	36	9	63	35
6	SLC6A6	−11.7	5.8e−09	147	12	25	20	22	37	31
7	TMPRSS4	−15.0	2.4e−11	128	10	29	34	8	31	16
1	CAR	−12.2	2.7e−09	107	7	28	18	9	26	19
2	DSG2	−14.0	1.3e−10	110	18	21	27	4	23	17
3	CD46	−13.0	7.2e−10	105	8	11	25	12	30	19
HAdV5 L5 protein										
1	SLC2A1	−13.6	2.4e−10	138	11	30	39	9	26	23
2	MET	−10.8	2.3e−08	93	9	25	21	13	19	6
3	IL1RAP	−8.5	9.8e−07	44	4	5	15	4	8	8
4	NPR3	−9.8	1.3e−07	55	3	6	14	1	13	18
5	GABRP	−15.7	8.7e−12	114	9	11	35	3	27	29
6	SLC6A6	−12.6	1.4e−09	129	11	28	30	13	28	19
7	TMPRSS4	−13.6	2.5e−10	96	11	18	19	6	28	14
1	CAR	−10.0	8.9e−08	59	4	13	14	5	13	10
2	DSG2	−11.1	1.4e−08	89	7	16	24	10	21	11
3	CR1(2MCZ)	−13.9	1.7e−10	87	5	12	22	4	25	19
4	CR1(5FO9)	−10.9	2.2e−08	94	6	11	9	19	31	18

The table represents the binding affinity and number of interactions of pancreatic cancer receptors, CAR, and DSG2 with HAdV2, HAdV3, and HAdV5 L5 proteins generated by the Prodigy server. Binding affinity (ΔG) represents the firmness with which two molecules bind with each other.

Kd, dissociation constant; ICs, interfacial contacts.

Cluster 2 was selected for the SLC2A1 interaction complex with the L5 protein HAdV2 (HADDOCK score −64.6 ± 16.0 and Z-score −1.8) (**Figure S7**). The interacting residues were predicted from Prodigy for SLC2A1. The SLC2A1 receptor forms a total of 141 interactions with the L5 protein (**Figure 2A** and **Table 4**). The information of interacting residues is provided in **Table S1**. The list of interacting residues includes 34 to 42 (*β* turn and H2 domain), 175 to 188 (3 *β* 1 *γ* 2 *β* turns and H10 domain), 295 to 308 (H18 domain and *β* turn), 355 to 366 (3 *β* turns corresponding to H22 domain), and 426 to 431 (H24 domain). The residues of two chains of the HAdV2 L5 protein complex interact with SLC2A1. In chain B, residue numbers include 439 to 443 (*β* turn), 523 to 528 (H5 domain and *β* turn), and 566 to 570 (*β* turn). In chain C, residue numbers include 412 to 415 (*β* hairpin), 449 to 460 (*β* turn and F stand), 469 to 472 (*β* and *γ* turn), 505 to 513 (*γ* turn and H4 domain), and 538 to 547 (*β* hairpin turn).

For MET, cluster 18 (HADDOCK score −79.5 ± 1.1 and Z-score −2.0) was selected out of all generated clusters (**Figure S8**). The domains of both A and B chains of MET dimer interact with HAdV2 L5, with 76 total interactions (**Figure 2B** and **Table 4**). Chain A residues include 557 to 562 (*β* and *γ* turn) and 639 to 646 (*β* hairpin turn). Chain B residues include 267 to 273 (*β* and *γ* hairpin turn), 308 to 311 (H3 domain), 381 to 384 (*β* turn), 392 to 398 (*β* turn hairpin), and 421 to 425 (F sheet). The residues of two chains of the HAdV2 L5 protein interact with the MET receptor. Chain B residue numbers include 409 to 417 (*β β* hairpin turn), 441 to 447 (H2 domain), 467 to 476 (*β γ β* turn), and 544 to 547 (*β β* hairpin turn). The C chain residues include 412 to 416 (*β* hairpin) and 449 to 451 (*β* turn).

Only one cluster with 4 conformations was generated for the interaction of IL1RAP and HAdV2 L5 in HADDOCK (**Figure S9**). The IL1RAP made a total of 75 interactions with HAdV2 L5, showing the highest number of interactions and better binding affinity out of all 3 L5 proteins (**Figure 2C** and **Table 4**). The IL1RAP receptor-interacting residues include 65 to 69 (J sheet) 77 to 86 (J sheet and H2 domain) and 117 to 122 (*β* hairpin turn). In the B chain of the L5 protein interacts with the IL1RAP receptor, its residues include 407 to 410 (*β* hairpin turn), 414 to 419 (*β* hairpin turn), 441 to 451 (*β β* turn and H2 domain), and 474 to 477 (*β* turn). For NPR3, cluster 3 (HADDOCK score −55.0 ± 14.4 and Z-score −1.4) was selected (**Figure S10**). Both chains of NPR3 receptor interact with HAdV2 L5, making a total of 75 interactions (**Figure 2D** and **Table 4**). Chain A residues include 124 and 125. Chain B residues include 47 to 52 (half A sheet), 81 to 91 (half H1 domain and *β β* turn), 95 to 100 (*β* turn and half A sheet), 121 to 124 (H2 domain), and 380 to 383 (*β β* turn). The B and C chains of the L5 protein interact with the NPR3 receptor. B chain residues include 439 to 447 (*β* turn and H2 domain), 523 to 528 (H5 domain, *β β* hairpin turn), and 566 to 570 (*β* turn). Chain C residues include 446 to 456 (H2 domain and *β* turn) and 507 to 512 (*γ* turn and H4 domain).

For GABRP, cluster 2 (HADDOCK score 85.9 ± 8.6 and Z-score −1.9) was selected (**Figure S11**). The GABRP receptor makes the highest number of contacts (169) with HAdV2 L5 out of all receptors, while GABRP makes a total of 168 contacts with HAdV3 (**Figure 2E** and **Table 4**). The residues of GABRP receptor interacting with HAdV2 L5 include 1 to 12 (H1 domain, *β β* turn, and start of H2 domain), 31 to 39 (two *β* turns), 70 to 73, 108 to 110 (*β* turn), 131 to 141 (*β* turn and A sheet), 204 to 211 (*α γ β* turn), and 241 to 244 (H7 domain). The two chains of the L5 protein interact with the GABRP receptor. Chain B residues include 402 to 410 (half E sheet end *β β* hairpin turn), 415 to 420 (*β* hairpin turn), 439 to 448 (*eta* turn and H2 domain), 474 to 477 (*β* turn), and 484 to 486 (*β* hairpin turn and E sheet). Chain C residues include 463 to 470 (*β γ* turn), 501 to 514 (*β β γ* turn and H4 domain), and 538 to 552 (H6 domain and *β β β* hairpin turn).

For SLC6A6, cluster 1 (HADDOCK score −115.8 ± 6.9 and Z-score −1.6) was selected (**Figure S12**). The SLC6A6 makes 84 contacts with the L5 protein (**Figure 2F** and **Table 4**), the list of interacting residues is as follows: 150 to 155 (*β β* turn), 163 to 167 (*β β* turn), 180 to 183 (*β* turn), 201 to 205 (loop), and 216 to 222 (H13 domain). The two chains of L5 proteins B and C interact with the SLC6A6 receptor. The residues of chain B include 403 to 420 (*β β β* hairpin turn), 468 to 477 (*β γ β* turn), and 483 to 486 (*β* hairpin and E sheet). Chain C residues include 501 to 507 (*β β β* turn) and 581 and 582. For TMPRSS4, cluster 8 (HADDOCK score 139.9 ± 9.5 and Z-score −1.9) was selected (**Figure S13**). The TMPRSS4 and L5 protein have a total of 119 interactions (**Figure 2G** and **Table 4**), and the list of interacting residues of TMPRSS4 is as follows: 227 to 230 (E sheet), 245 to 251 (*β* turn), 282 to 290 (*β γ β* hairpin turn), 331 to 339 (*β* hairpin turn), 363 to 365 (*γ β β* hairpin turn), 383 to 387 (*β β* turn), and 405 to 410 (*β* hairpin turn). Chain B of the L5 protein interacts with TMPRSS4, and its residues are 406 to 420 (*β β β* hairpin turn), 439 to 450 (*β* turn and H2 domain), and 469 to 477 (*β γ β* turn); other interacting residues include 488, 490, 544, 547, and 566. The secondary structure of SLC2A1, MET, IL1RAP, NPR3, GABRP, SLC6A6, and TMPRSS4 showing the interacting domains of receptors is given in **Figure S38**. The secondary structure of the HAdV2, HAdV3, and HAdV5 L5 proteins showing the interacting domains of receptors is given in **Figure S37**. The secondary structure of SLC2A1, MET, IL1RAP, NPR3, GABRP, SLC6A6, and TMPRSS4 showing the interacting domains of receptors is given in **Figure S38**. The list of interacting residues is provided in **Table S1**.

#### 3.2.2 CAR and DSG2

**Figure 3:**
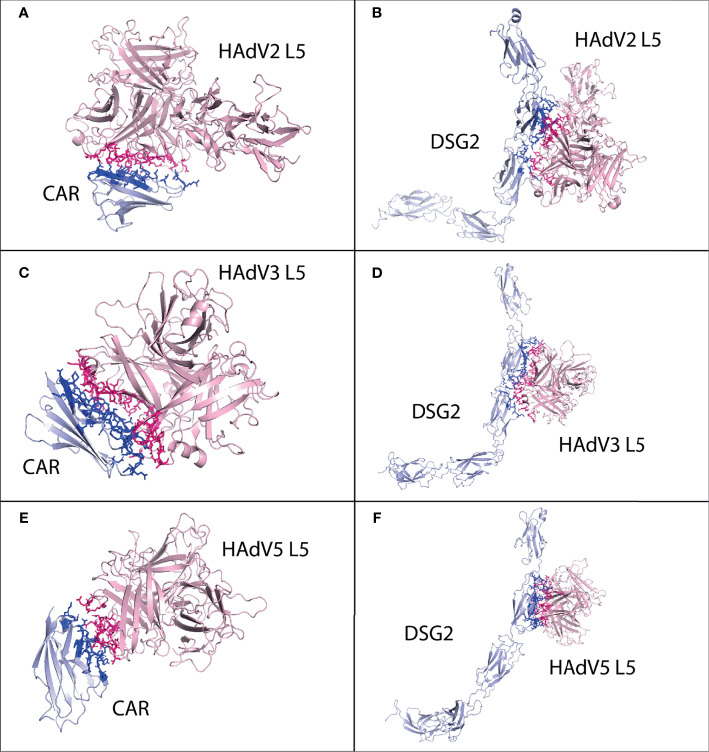
Protein–protein interaction complex of HAdV2, HAdV3, and HAdV5 L5 protein with human CAR, DSG2, and CR1 receptors. **(A)** CAR made a total of 63 interactions with HAdV2 L5 protein. **(B)** DSG2 made 86 interactions with HAdV2 L5 protein. **(C)** CAR made 107 interactions with HAdV3 L5 protein. **(D)** DSG2 made 110 interactions with HAdV3 L5 protein. **(E)** CAR made 59 interactions with HAdV5 L5 protein. **(F)** DSG2 made 89 interactions with HAdV5 L5 protein.

The best possible interaction complex for CAR and DSG2 was obtained after docking, with the best HADDOCK score and lowest Z-score then selected for refinement. For CAR, cluster 3 (HADDOCK score −57.2 ± 11.9 and Z-score −1.0) was selected (**Figure S14**). The CAR made a total of 63 interactions with the HAdV2 L5 protein, shown in **Figure 3A**, and the details of the interactions are provided in **Table 4**. The interacting residues of CAR receptor include 16 to 18 (*β* turn), 27 to 44(B sheet, *β* turn and C sheet), 94 to 100 (*β* hairpin turn and H1 domain), and 102, 104, and 106. Chains B and C of the L5 protein trimer interact with the CAR receptor. The residues of chain B include 406 to 410 (*β* hairpin loop), 416 to 420 (*ta* hairpin loop), 474 to 477 (*β* turn), 484, and 485. The residues of chain C include 501 to 509 (*β γ* turn), 548 to 550 (*β* hairpin loop), and 581.

For DSG2, cluster 5 (HADDOCK score −66.5 ± 11.8 and Z-score −1.1) was selected (**Figure S15**). The DSG2 made a total of 86 interactions with the HAdV2 L5 protein, shown in **Figure 3B**, and the details of the interactions are provided in **Table 4**. The interacting residues of DSG2 receptor include 200 to 208 (half D sheet, *β γ β* turn), 230 to 232, 237 to 242 (half D sheet), 251 to 257 (*β* hairpin loop), 294 to 298 (*β* turn), and 271, 273, 275, and 301. Chains A and B of the L5 protein trimer interact with the DSG2 receptor. The residues of chain A include 408, 409 (*β* hairpin loop), 468 to 477 (*β γ β* turn), and 482 to 485 (E sheet, *β* hairpin loop). The residues of chain B include 461 to 464 (*ta* turn), 501, 502, 506 (*β* turn), 541 to 551 (*β β* hairpin loop), and 581. The list of interacting residues is provided in **Table S2**.

### 3.3 Protein–Protein Interaction of HAdV3 L5 Protein With Receptors

#### 3.3.1 Pancreatic Cancer Receptors

**Figure 4:**
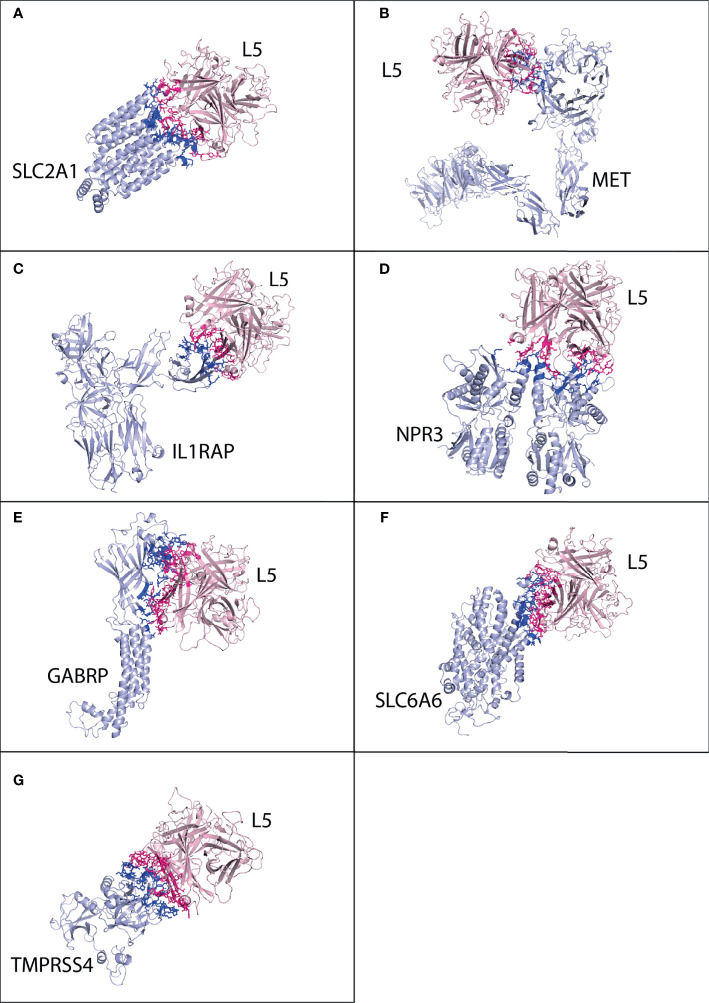
Protein–protein interaction complex of HAdV3 L5 protein with pancreatic cancer receptors. The pink ribbon represents L5 protein, while purple ribbons represent cancer receptors. The interacting residues are shown by a stick, L5 protein is shown in dark pink, and receptor is shown in dark blue. **(A)** SLC2A1 made a total of 118 interactions. **(B)** MET made 73 interactions. **(C)** IL1RAP made 62 interactions. **(D)** NPR3 made 92 interactions. **(E)** GABRP made 168 interactions. **(F)** SLC6A6 made 147 interactions. **(G)** TMPRSS4 made 128 interactions.

The best possible interaction complex for all receptors was obtained after docking, with the best HADDOCK score and lowest Z-score then selected for refinement. For SLC2A1, cluster 3 (HADDOCK score 126.4 ± 2.4 and Z-score −1.7) was selected (**Figure S16**). The SLC2A1 made a total of 118 interactions with the HAdV3 L5 protein, shown in **Figure 4A**, and the details of the interactions are provided in **Table 4**. The interacting residues of the SLC2A1 receptor include 55 to 57, 114 to 117 (H6 domain), 175 to 177 (*β* turn), 181 to 183 (*γ* turn), 299 to 300 (H18 domain), 304 to 308 (start of H19 domain), 355 to 361 (*β*, *β* turn), and 426 to 432 (partial H24 domain). Chains A and C of the L5 protein trimer interact with the SLC2A1 receptor. The residues of chain A include 174 to 181 (half A sheet and half H1 domain), 261 to 263 (*β* hairpin turn), and 300 to 304 (*β* turn). The residues of chain C include 188 to 196 (B sheet after H1 domain), 242 to 246 (*γ* and *β* turn), 289 to 293 (B sheet), 276, 279, 147, and 149.

For MET, cluster 5 (HADDOCK score −70.6 ± 7.2 and Z-score −2.4) was selected (**Figure S17**). Chain B of MET receptor dimer made a total of 73 interactions with the L5 protein (**Figure 4B** and **Table 4**). Its residues include 240 and 241 (H2 domain), 268 to 271 (*β γ* hairpin turn), 379 to 384 (*β* turn and F sheet), 392 to 404 (*β β* hairpin turn), and 419 and 421. The two chains of the L5 protein interact with the MET receptor. Chain A residues include 260 to 263 (*ta* hairpin turn). Chain C residues include 197 to 205 (*β* turn), 249 to 253 (B sheet), and 281 to 288 (*β* hairpin turn and half E sheet); other interacting residues include 296, 298, 154, 156, and 217. For IL1RAP, cluster 4 (HADDOCK score −44.2 ± 4.5 and Z-score −1.6) was selected (**Figure S18**). Chain C residues of the IL1RP receptor complex interact with the L5 protein, making a total of 62 contacts (**Figure 4C** and **Table 4**). The residues include 77 to 96 (J sheet, H2, H3 domains *β* turn and k sheet) and 115 to 119 (half of J sheet and *β* hairpin turn). The two chains are the L5 protein interacting with the IL1RAP receptor. Chain A residues include 258 to 264 (*β* hairpin turn). Chain C residues include 190 to 194 (B sheet), 241 to 43 (*γ* and *β* turn), 250, 252, 261, 277 to 281 (*β* hairpin turn), and 289 to 293 (B sheet).

For NPR3, cluster 4 (HADDOCK score −81.0 ± 7.4 and Z-score −1.3) was selected (**Figure S19**). Two chains of NPR3 receptor complex interact with the L5 protein, making the highest number of interactions 92 out of all serotypes (**Figure 4D** and **Table 4**). Chain A residues include 119 to 126 (half H2 domain and *β* sheet). Chain B residues are 47 to 54 (half A sheet), 95 to 99 (*β* turn), 102 to 104 (A sheet before H2 domain), and 118 to 126 (half H2 domain and B sheet). The two chains of the L5 protein interact with the NPR3 receptor. The residues of chain A include 178, 257 to 261 (*β* hairpin turn), and 299 to 304 (*β* turn). The residues of chains B include 145 to 150 (*β* hairpin turn), 182 to 196 (H1 domain), 241 to 247 (*γ β* turn), 278, 289, and 179.

For GABRP, cluster 2 (HADDOCK score 71.4 ± 8.2 and Z-score −2.5) was selected (**Figure S20**). The GABRP receptor also shows the best binding affinity with HAdV3 L5, making 168 interactions (**Figure 4E** and **Table 4**). The interacting residues of GABRP receptor include 1 to 20 (H1 domain, *β*, *β*, *β*, *β* turn, H2 domain, *β* turn), 28 to 34 (*β* turn), 71 to 74 (half A sheet), 132 to 141 (*β* turn, A sheet), 203 to 211 (*β*, *γ*, *β* turn), and 241 to 244 (H7 domain). The two chains of the L5 protein interact with the GABRP receptor. Chain A residues include 174 to 178 (A sheet before H1 domain), 257 to 261 (*β* hairpin turn), and 299 to 304 (*β* turn). The C chain residues include 144 to 149 (*β* hairpin turn), 178 to 194 (H1 domain and B sheet), 240 to 247 (*γ β* turn), 275 to 280 (*β* hairpin turn), 300 to 302, and 250.

For SLC6A6, cluster 2 (HADDOCK score 4.8 ± 10.8 and Z-score −1.8) was selected (**Figure S21**). SLC6A6 shows better binding affinity (−11.7 kcal mol^−1^) and the highest interactions (147) with HAdV3 than other serotypes (**Figure 4F** and **Table 4**). The interacting residues of SLC6A6 receptors include 154 to 158 (*β* turn), 163 to 170 (*β*, *β* turn), 178 to 190 (*β*, *β*, *β* turn), 197 to 203 (H12 domain), and 215 to 222 (H13 domain); other residues in fluids include 449, 147, and 151. The L5 protein residues that interact with SLC6A6 are chain A 258 to 265 (*β* hairpin turn). Chain C residues include 145 to 151 (*β* hairpin turn), 182 to 196 (H1 domain and B sheet), 268 to 279 (half B sheet, *γ β* hairpin turn), and 287 to 293 (B sheet). For TMPRSS4, cluster 3 (HADDOCK score 153.1 ± 2.7 and Z-score −1.2) was selected (**Figure S22**). TMPRSS4 also makes the highest number of interactions with HAdV3 128 (**Figure 4G** and **Table 4**). The residues of TMPRSS4 include 224 to 28 (*β*, *β*, *γ* hairpin turn and F sheet), 245 to 257 (*β*, *β*, *β* turn and F sheet), 276 to 285 (half E sheet and *β*, *γ* hairpin turn), 331 to 339 (*β* hairpin turn), 384 to 387 (*β*, *β* turn), and 405 to 408 (*β* turn). The two chains of the L5 protein interact with TMPRSS4. Chain A residues include 258 to 263 (*β* hairpin turn). Chain C residues include 146 to 150 (*β* hairpin turn), 178 to 183 (half H1 domain), 185 to 196 (B sheet after H1 domain), 241 to 247 (*γ*, *β* turn), 275 to 281 (*γ*, *β* hairpin turn), and 285 to 293 (B sheet). The list of interacting residues for all interaction complexes is provided in **Table S1**.

#### 3.3.2 CAR, DSG2, and CD46

The best possible interaction complex for CAR and DSG2 receptors was obtained after docking, with the best HADDOCK score and lowest Z-score then selected for refinement. For CAR, cluster 1 (HADDOCK score −78.2 ± 4.0 and Z-score −1.3) was selected (**Figure S23**). The CAR made a total of 107 interactions with the HAdV3 L5 protein, shown in **Figure 3C**, and the details of the interactions are provided in **Table 4**. The interacting residues of CAR receptor include 16 to 18 (*β* turn), 23 to 50 (*β* turn, B sheet, *β* turn, C sheet, *β* and *γ* turn), 92 to 109 (*β* hairpin turn, H1 domain, and C sheet), and 131. Chains A and C of the L5 protein trimer interact with the CAR receptor. The residues of chain A include 258 to 265 (*β* hairpin loop) and 296 to 301 (*β* turn). The residues of chain C include 146 to 149 (*β* hairpin loop), 178 to 196 (H1 domain, *β* turn and B sheet), 242, 244, 245, 250 (*γ* and *β* turn), and 291 to 294 (half B sheet).

For DSG2, cluster 1 (HADDOCK score −82.4 ± 9.2 and Z-score −1.1) was selected (**Figure S24**). The DSG2 made a total of 110 interactions with the HAdV3 L5 protein, shown in **Figure 3D**, and the details of the interactions are provided in **Table 4**. The interacting residues of DSG2 receptor include 199 to 210 (half D sheet, *β γ β* turn), 223 to 232 (*β β β* turn), 247 to 250 (*β* hairpin loop), 272 to 275 (*β* turn), 294 to 297 (half F sheet), 301, and 306. Chains A and C of the L5 protein trimer interact with the DSG2 receptor. The residues of chain A include 146, 149, 178, 179, and 299 to 302 (*β* turn). The residues of chain C include 145 to 153 (*β* hairpin loop), 177 to 179, 190 to 196 (half B sheet), 241 to 245 (*γ β* turn), 272 to 278 (*γ* hairpin loop), and 285 to 289 (B sheet).

**Figure 5:**
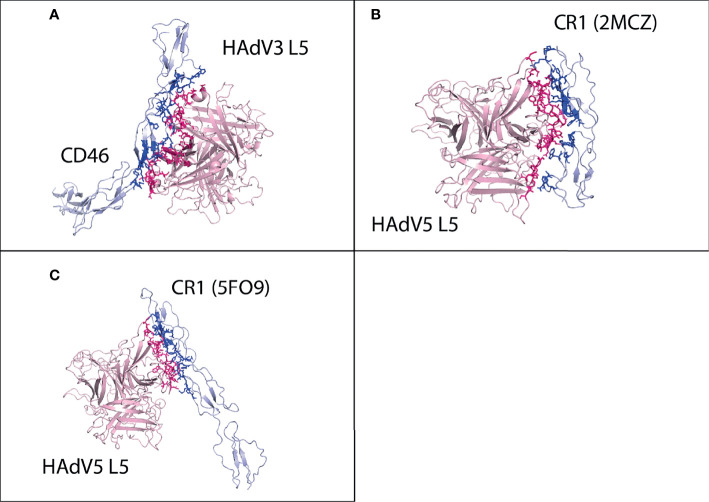
Protein–protein HAdV3 and HAdV5 L5 protein with human CD46 and CR1 receptors. **(A)** CD46 made a total of 105 interactions with HAdV3 L5 protein. **(B)** CR1 domains 1 and 2 made 87 interactions with HAdV5 L5 protein. **(C)** CR1 domains 15, 16, and 17 made 94 interactions with HAdV5 L5 protein.

For CD46, cluster 1 (HADDOCK score −83.8 ± 13.2 and Z-score −1.7) was selected (**Figure S25**). The CD46 made a total of 105 interactions with the HAdV3 L5 protein, shown in **Figure 5A**, and the details of the interactions are provided in **Table 4**. The interacting residues of CD46 receptor include 69 to 73 (*β* turn), 92 to 109 (H1 domain, B sheet, *β* turn, and half C sheet), 112, 115 to 118 (*γ* hairpin loop), 126 to 131 (*β* turn), 148, 149, 156 to 159 (D sheet), and 182 to 184 (*β* hairpin loop). Chains A and C of the L5 protein trimer interact with the CD46 receptor. The residues of chain A include 178, 182, 185, 258 to 262 (*β* hairpin loop), and 298 to 303. The residues of chain C include 145 to 149 (*β* hairpin loop), 178, 179, 182, 185 to 196 (*β* turn, B sheet), 242 to 247 (*β* turn, H4 domain), and 287 to 293 (B sheet). The list of interacting residues for all interaction complexes is provided in **Table S2**.

### 3.4 Protein–Protein Interaction of HAdV5 L5 Protein With Receptors

#### 3.4.1 Pancreatic Cancer Receptors

**Figure 6:**
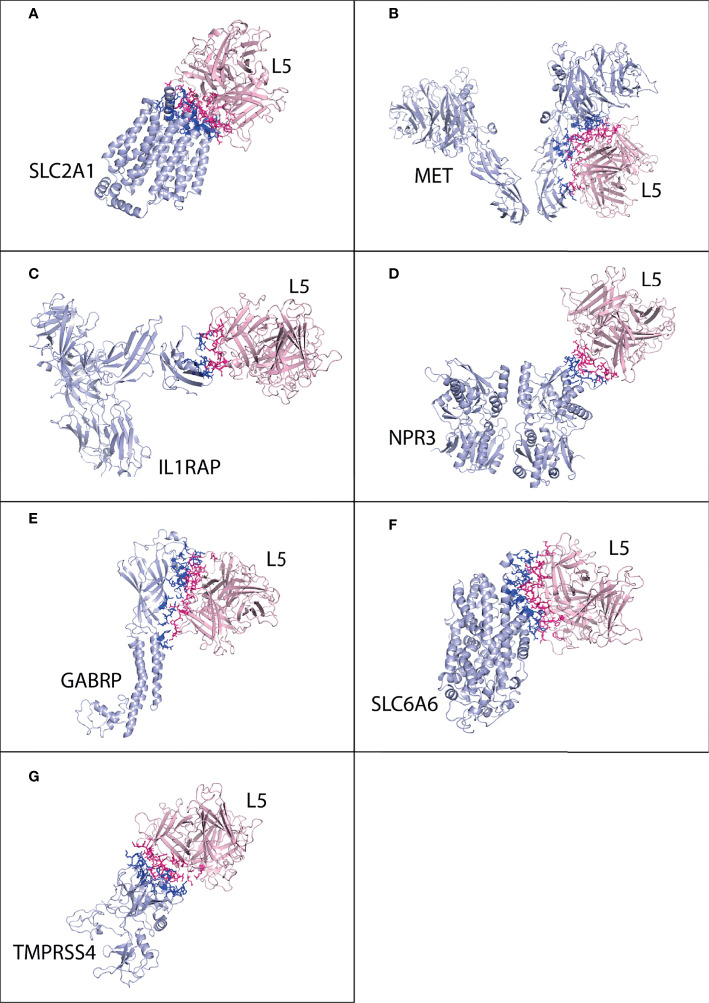
Protein–protein interaction complex of HAdV5 L5 protein with pancreatic cancer receptors. The pink ribbon represents L5 protein, while purple ribbons represent cancer receptors. The interacting residues are shown by a stick, L5 protein is shown in dark pink, and receptor is shown in dark blue. **(A)** SLC2A1 made a total of 138 interactions. **(B)** MET made 93 interactions. **(C)** IL1RAP made 44 interactions. **(D)** NPR3 made 55 interactions. **(E)** GABRP made 114 interactions. **(F)** SLC6A6 made 129 interactions. **(G)** TMPRSS4 made 96 interactions.

The best possible interaction complex generated by docking (HADDOCK) was then selected for refinement and interaction analysis. For SLC2A1, cluster 4 (HADDOCK score −66.3 ± 3.8 and Z-score −1.4) was selected (**Figure S26**). The interaction analysis determines that SLC2A1 makes a total of 138 contacts with HAdV5 L5, shown in **Figure 6A** and **Table 4**. The interacting residues of SLC2A1 include 37 to 45 (half H3 domain), 54 to 61 (loop between H2 and H3), 296 to 305 (half H18, *β* turn), 356 to 362 (*β*, *β*, *β* turn), and 426 to 432 (part of H24 domain). The two chains of the L5 protein interact with SLC2A1. Chain A residues include 396 to 399 (*β*, *β* turn), 404 to 410 (*β* hairpin turn), 476-477 (H2 domain), and 481 to 492 (*β* hairpin turn, A sheet). Chain C residues include 501 to 513 (*β*, *β* turn and H4 domain).

For MET, cluster 36 (HADDOCK score −78.8 ± 3.7 and Z-score −1.7) was selected (**Figure S27**). The MET receptor makes the highest number of contacts (93) with HAdV5 with a better binding affinity (−10.8 kcal mol^−1^) (**Figure 6B** and **Table 4**). The residues of MET receptor include 566 to 569 (J sheet and *β* turn), 582 to 586 (J sheet), 615 to 623 (H10 domain and *β* hairpin turn), and 700 to 706 (M sheet and *β* hairpin turn). Other residents include 40, 459 to 461, 521, 528, and 553. Chains A and B of the L5 protein interact with MET. Chain A residues include 396 to 399 (*β*, v turn), 476 to 477 (H2 domain), and 481 to 496 (*β* hairpin turn, A sheet, and H3 domain). Other important residues include 580 and 581. For IL1RAP, cluster 5 (HADDOCK score −44.2 ± 4.5 and Z-score −1.6) was selected (**Figure S28**). The IL1RAP makes a total of 44 interactions with HAdV5 (**Figure 6C** and **Table 4**). The residues of the IL1RAP receptor include 77 to 86 (J sheet and H2 domain) and 115 to 118 (J sheet); other residues are 70 and 71. Chain A of the L5 protein interacts with the IL1RAP; residues include 469 to 479 (*β*, *β* turn, H2 domain, and half A sheet) and 485 to 492 (A sheet and loop).

For NPR3, cluster 9 (HADDOCK score −52.7 ± 11.0 and Z-score −2.1) was selected (**Figure S29**). The NPR3 makes a total of 55 interactions (**Figure 6D** and **Table 4**). Chain B of NPR3 receptor dimer interacts with the L5 protein, its residues include 47 to 50 (loop and half A sheet) and 88 to 97 (v turn), and other residues are 373 and 377. NPR3 chain A of the L5 protein interacts with residue numbers 466 to 469 (*β* turn), 474 to 477 (H2 domain), 482 to 492 (*β* hairpin turn and A sheet), and 404. For GABRP, cluster 12 (HADDOCK score 63.8 ± 5.4 and Z-score −2.3) was selected (**Figure S30**). The GABRP makes a total of 114 interactions with HAdV5 L5 (**Figure 6E** and **Table 4**). The interacting residues of the GABRP receptor with the L5 protein are 4 to 12 (H1 domain, *β* v *β β* turn and half H2 domain), 29 to 38 (*β β* turn), 71 to 74 (half A sheet), 131 to 141 (half A sheet), 204 to 209 (*β γ β* turn), and 243 to 248 (H7 domain). For GABRP, the two chains of the L5 protein interact with the receptor. Chain A residues include 404 to 409 (*β* hairpin turn), 474 to 479 (H2 domain), 481 to 490 (*β* hairpin turn and A sheet), and 466. Chain B residues include 501 to 508 (*α β β* turn), 543 to 550 (*γ β γ* turn and *β* hairpin turn), and 581.

For SLC6A6, cluster 1 (HADDOCK score −76.9 ± 2.7 and Z-score of −1.5) was selected (**Figure S31**). The SLC6A6 makes a total of 129 interactions with the HAdV5 L5 protein (**Figure 6F** and **Table 4**). The list of interacting residues of SLC6A6 is 152 to 170 (*β*, *β* turn), 179 to 185 (*β* turn), and 201 to 220 (*β*, *β* turn and start of H13 domain); other important residues include 147, 151, 154, and 158. Chain A residues of the L5 protein interact with SLC6A6 including 396 to 399 (*β*, *β* turn), 404 to 409 (*β*, *β* hairpin turn), and 474 to 490 (H2 domain, A sheet, *β* hairpin turn, and A sheet). Chain B residues include 492 to 497 (A sheet), 580, and 581. For TMPRSS4, cluster 1 (HADDOCK score 88.5 ± 0.9 and Z-score of −1.0) was selected (**Figure S32**). The TMPRSS4 makes a total of 96 interactions with the HAdV5 L5 protein (**Figure 6G** and **Table 4**). The interacting residues of TMPRSS4 include 227 to 230 (E sheet), 248 to 250 (*β* turn), 282 to 287 (*β*, *γ* hairpin turn), 331 to 339 (*β* hairpin turn), 382 to 387 (*β*, *β* turn), and 405 to 408 (*β* turn). The residues of chain A of the L5 protein interact with TMPRSS4 including 396 to 398 (*β*, *β* turn), 463 to 469 (*β* turn) 474 to 497 (H2 domain, A sheet, *β* hairpin turn, A sheet), and 581. The list of interacting residues for all interaction complexes is provided in **Table S1**.

#### 3.4.2 CAR, DSG2, and CR1

The best possible interaction complex for all receptors was obtained after docking, with the best HADDOCK score and lowest Z-score then selected for refinement. For CAR, cluster 2 (HADDOCK score −66.9 ± 2.3 and Z-score −1.6) was selected (**Figure S33**). The CAR made a total of 59 interactions with the HAdV5 L5 protein, shown in **Figure 3E**, and the details of the interactions are provided in **Table 4**. The interacting residues of CAR receptor include 33, 34, 79 to 93 (*β β eta* turn and C sheet), 106 to 111 (C sheet and *β* turn), and 113. Chain A of the L5 protein trimer interacts with the CAR receptor. The residues of chain A include 412, 416, 417 (*β* hairpin loop), 466 to 479 (*β* turn and H2 domain), and 487 to 492 (A sheet).

For DSG2, cluster 3 (HADDOCK score 126.4 ± 2.4 and Z-score −1.7) was selected (**Figure S34**). The DSG2 made a total of 89 interactions with the HAdV5 L5 protein, shown in **Figure 3F**, and the details of the interactions are provided in **Table 4**. The interacting residues of CAR receptor include 174 to 177 (E sheet), 189 to 196, 198 to 200 (*β β* turn, and half D sheet), 207 to 209 (*γ β* turn), 212 to 219 (E sheet, *β* hairpin loop), 221 to 225 (E sheet), 245, and 247. Chain A of the HAdV5 L5 protein interacts with the DSG2 receptor. The residues of chain A include 417, 461 to 479 (*β β* turn and H2 domain), 482 to 492 (*β* hairpin loop and A sheet), and 544 to 549 (*γ β* hairpin loop).

For CR1 domain 1 and 2 interaction with HAdV5, cluster 3 (HADDOCK score −86.1 ± 8.3 and Z-score −1.8) was selected (**Figure S35**). The CR1 domains 1 and 2 made a total of 87 interactions with the HAdV5 L5 protein, shown in **Figure 5B**, and the details of the interactions are provided in **Table 4**. The interacting residues of the CR1 receptor include 7 to 12 (*β* turn), 33, 34, 75 to 78 (*β* hairpin loop), 82 to 87 (*β* hairpin loop), and 95 to 106 (*β γ* turn). Chains A and B of the HAdV5 L5 protein interact with the receptor. The residues of chain A include 404 to 408 (*β* hairpin loop), 462 to 477 (*β β β* turn and H2 domain), 482 to 492 (*β* hairpin loop and A sheet), 544, and 546. Chain B residues include 501 to 508 (*β β β* turn), 548, and 549. The interaction profile of CR1 domains 15, 16, and 17 with the HAdV5 L5 protein was also determined. Cluster 1 (HADDOCK score −75.9 ± 4.4 and Z-score −2.5) was selected (**Figure S36**). The CR1 domains 15, 16, and 17 made a total of 94 interactions with the HAdV5 L5 protein, shown in **Figure 5C**, and the details of the interactions are provided in **Table 4**. The interacting residues of the CR1 receptor include 1,012 to 1,016 (W sheet), 1,032 to 1,035 (v turn), 1,078 to 1,081 (Y sheet and *β* hairpin loop), 1,087 to 1,094 (*β* hairpin loop and Y sheet), and 1,110 to 1,119 (Y sheet and *β* hairpin loop). Chain A of the HAdV5 L5 protein interacts with the receptor. The residues of chain A include 404, 417, 462 to 471 (*β β β* turn), 474 to 479 (H2 domain), 485 to 493 (A sheet), and 544 to 547 (*γ* hairpin loop). The secondary structures of CAR, DSG2, and CR1 showing the interacting domains of receptors are given in **Figure S39**. The list of interacting residues for all interaction complexes is provided in **Table S2**.

## 4 Discussion

Pancreatic cancer is the most fatal malignancy because of its dynamic molecular nature. There are many therapies against pancreatic cancer, but all therapies have very low efficacy, so there is a dire need to explore new therapeutic ways. Oncolytic virus therapy is an innovative therapy with fewer side effects than chemotherapy. In this therapy, viruses are injected into the cancerous region; viruses kill the cancer cells and activate patient anticancer immune responses. The mode of entry of oncolytic viruses in cancer cells completely depends on the viral attachment on the cell surface, which mainly depends on the strength of interactions of the viral L5 protein with the cancer cell surface receptors. In our previous study, we analyzed the expression profile of differentiated genes in the pancreatic cancer cell (23). In that particular study, 5 microarray and 2 RNA-seq datasets were analyzed. We identified 7 highly expressed pancreatic cancer cell surface receptors (MET, NPR3, IL1RAP, SLC2A1, SLC6A6, GABRP, and TMPRSS4) in all the analyzed datasets. In the current study, interaction analysis of these seven receptors with the L5 protein of adenovirus type C2 (HAdV2), B3 (HAdV3), and C5 (HAdV5) is performed. Adenovirus type HAdV2 and HAdV5 are mostly used as oncolytic viruses. The L5 proteins of HAdV2, HAdV3, and HAdV5 are reported to interact with the cell surface receptors CAR, DSG3, CD46, and CR1 (13–16). The CAR and DSG2 gene underexpression is reported in pancreatic cancer cells, due to the high expression of MEK and ERK (20, 21). In this study, we have explored the interaction profile of pancreatic cancer receptors with the L5 proteins to analyze the efficacy of adenovirus as an oncolytic virus. Also, we performed an interaction analysis of the L5 protein with CAR, DSG2, CD46, and CR1 for the comparison of binding affinities with pancreatic cancer receptors.

Asp406, Ser408, Pro409, Arg412, Lys420, and Tyr477 are important interacting residues of HAdV2 with the CAR human receptor. Mutation in these residues can result in a decreased binding affinity with the receptor (62, 63). In our study, docking analysis of CAR and DSG2 with the HAdV2 L5 protein was performed, and these important residues are identified in the interaction complexes. These residues are also reported to interact with pancreatic cancer surface receptors. All six residues interact with the surface binding site of SLC6A6 and TMPRSS4 receptors. The GABRP binding site residues interact with all the abovementioned residues except for Arg412. Residues Asp406, Arg412, and Lys420 interact with SLC2A1, while MET receptor interacts with Asp406, Pro409, Arg412, and Lys420. The residue numbers of Ser408, Pro409, and Tyr477 of the HAdV2 L5 protein interact with IL1RAP; out of 6 important residues, NPR3 show interaction only with Lys420. Other than those residues that are reported in previous literature, Val441 and Ser442 also interact with all the receptors.

DSG2 is a cadherin junctional adhesion protein that mediates the HAdV3 entry in the cell. The important residues of the HAdV3 L5 protein required for interaction with human DSG2 receptor include Asn186, Val189, Ser190, Asp261, Phe265, Leu292, and Glu299 (14, 64). The literature reported that residues of the HAdV3 L5 protein are identified to be important in docking analysis for interacting with DSG2, CAR, and CD46. These important residues are also reported to interact in our pancreatic cancer receptor analysis. The GABRP receptor interacts with all the above-listed residues except for Phe265. The NPR3 interacts with Asn186, Val189, Ser190, Asp261, and Glu299. The TMPRSS4 interacts with Asn186, Val189, Ser190, Asp261, and Leu292. The SLC6A6 interacts with Asn186, Val189, Ser190, Asp265, and Leu292. Other than these residues, Asn192 shows interaction with all pancreatic cancer receptors.

The important interacting residues of the HAdV5 L5 protein include Ser408, Pro409A, Lys417G (AB loop) (65), Tyr477 (DE loop), and Leu485Lys in *β* -strand F and include TAYT (Thr489, Ala490, Try491, and Thr492 of FG loop). The replacement mutation or deletion of any residue out of the above-given list results in ablating the HAdV5 L5 protein interaction with the CAR receptor (62). Another literature reported that in addition to the above residues, Ala406 and Arg481 are also important for the interaction of the HAdV5 L5 protein (64). We also performed the docking of the HAdV5 L5 protein with CAR, DSG2, and CR1 to determine the importance of these residues. Residues Lys417, Tyr477, Leu485, and TAYT (Thr489, Ala490, Try491, and Thr492) are identified to interact with all three receptors. The interaction of these important residues with pancreatic cancer receptors is also analyzed. Out of the above residues listed, only Arg412 does not show interactions with any of the receptors. The SLC2A1 receptor interacts with all the residues except for Glu416, while SLC6A6 also interacts with all residues except for Try491. The most important residues of the L5 protein that interact with all the pancreatic cancer receptors are Glu476, Try477, Leu485, Glu487, Gly488, Thr489, ALa490, and Thr492.

The SLC2A1 receptor shows a high binding affinity with all three L5 proteins. The list of SLC2A1 residues that show interaction with all three L5 proteins is Asp177, Asn182, Lys183, Glu299, Lys300, Gln304, Leu358 to Leu361 (LEQL, *β* turn), and Glu426 to Pro431 (ELCGP, H24 domain). The MET has the lowest binding affinity with the L5 protein, out of all the receptors. Only HAdV5 makes the most number of interactions with a total of 93 with a binding affinity of −10.8. MET has no common residues that interact with all L5 proteins. The IL1RAP shows a high binding affinity of −11.5 kcal mol^−1^ with a total of 75 interactions with HAdV2. The list of IL1RAP residues, which show interaction with all three L5 proteins, is Arg77 to Ile83 (RDLEEPI, J sheet, and H2 domain), Phe85, Arg86, Arg117, Asn118, and Thr119 (RNT). NPR3 shows the highest binding affinity with HAdV3 L5 with a total of 92 interactions. The NPR3 residues, which show interaction with all three L5 proteins, are Ala47, Leu48, Pro49, Pro50 (ALPP, A sheet), Pro95, Gly96, and Thr97 (PGT, *β* turn).

The GABRP shows the highest number of interactions with all the L5 proteins with a binding affinity of more than −15 kcal mol^−1^. The GABRP residues that show interaction with all three L5 proteins are Met1, Ser4, Leu5, Leu7, Ala8, Phe9, Cys11, Leu12 (H1 domain, *β* turn and H2 domain), Gly31 to Asp34 (GRSD, *β* turn), Ser37, Ser71 to Ser73 (SIS, A sheet), Glu132 to Asn137 (EVTVGN, *β* turn and A sheet), Leu139, Arg141, His204 to Gln209 (HLRLAQ, *β γ β* turn), Leu243, and Tyr244. The list of SLC6A6 residues that show interaction with all three L5 proteins is Tyr147, Gln151, Gln154, Asn163, His164, Ser165, Trp166, Asn167 (NHSWN, *β* turn), Lys180, Ser181, Val182, Ser183 (KSVS, *β* turn), Arg201, Asn202, Val203 (RNV, H12 domain), and Leu216, Lys217, Trp218, and Asp219 (LKWD, H13 domain). The list of TMPRSS4 residues that show interaction with all three L5 proteins is Gln227, His228, His245, Arg248, Lys249, His250 (RKH, *β* turn), Asn282 to Lys287 (NPMYPK, *β γ* hairpin turn), Phe331 to Met339 (FQNGGKM, *β* hairpin turn), Gln384, Gly385, Ser387, and Ser405 to Tyr408 (SWGY, *β* turn).

In this study, we attempt to identify better interacting adenovirus L5 protein with pancreatic cancer receptors, so that serotype can be used more effectively as an oncolytic virus. The HAdV3 L5 shows better interaction with 4 receptors NPR3, GABRP, SLC6A6, and TMPRSS4 with the highest number of interactions and better binding affinity. HAdV2 shows a better interaction profile with SLC2A1 and IL1RAP with high binding affinity, while HAdV5 interacts well with MET. The affinity maturation can be performed by mutating the fiber knob of the HAdV3 to enhance its interaction with the SLC2A1, MET, and IL1RAP specifically and also escalate the binding affinities with other receptors. David Curiel’s lab research was able to increase the efficacy of HAdV5 against prostate cancer by recombination of HAdV5 with the HAdV3 L5 protein (64, 66). In an experimental study (67), analyzed the efficacy of chimeric HAdV5/HAdV3 modified fiber virus against pancreatic cancer as a chemoprevention gene therapy. They have used fiber knob HAdV3 instead of HAdV5 to retain high infectivity. As the CAR is reduced in most cancer and affects the infectivity *via* HAdV5. They have found that (Ad.5/3-CTV-M7) has an antitumor effect on primary and distant site tumor growth (in metastasis). This study showed that Ad.5/3-CTV-M7 could be utilized as a therapy against pancreatic cancer. Our study also exhibited the same outcome, as the HAdV3 L5 protein interacts better than the HAdV2 and HAdV5 L5 proteins with pancreatic cancer cell receptors.

## 5 Conclusion

Oncolytic virus therapy is a promising immunotherapy approach that can be used for the treatment of pancreatic cancer. The virus’s entry entirely depends on the attachment of the virus with the cancer cell surface receptors. In the current study, the attachment or interaction profile of 3 adenovirus serotypes with the 7 pancreatic cancer cell receptors is analyzed. The L5 proteins of 3 serotypes of adenovirus HAdV2, HAdV3, and HAdV5 are docked with the seven highly expressed pancreatic cancer receptors SLC2A1, MET, IL1RAP, NPR3, GABRP, SLC6A6, and TMPRSS4. The binding affinity of the L5 proteins with human receptors is analyzed. The HAdV3 L5 protein displays a better interaction profile and high binding affinities with 4 receptors NPR3, GABRP, SLC6A6, and TMPRSS4, while HAdV2 shows better results with SLC2A1 and IL1RAP. It is proposed that affinity maturation of HAdV3 can be performed by incorporating the important interacting residues of HAdV2 and HAdV5, to enhance its binding affinity with the remaining 3 receptors SLC2A1, MET, and IL1RAP. In the future, we will perform a wet lab analysis for these interactions to further validate our results.

## Data Availability Statement

The original contributions presented in the study are included in the article/**Supplementary Material**. Further inquiries can be directed to the corresponding author.

## Ethics Statement

Ethical approval/written informed consent was not required for the study of animals/human participants in accordance with the local legislation and institutional requirements.

## Author Contributions

MN and RP conceived and designed the study. MN conducted the analysis and wrote the manuscript. All authors took part in the analytical discussions and critical review of the manuscript.

## Funding

The National University of Sciences and Technology (NUST) will pay the publication fee for this article.

## Conflict of Interest

The authors declare that the research was conducted in the absence of any commercial or financial relationships that could be construed as a potential conflict of interest.

## Publisher’s Note

All claims expressed in this article are solely those of the authors and do not necessarily represent those of their affiliated organizations, or those of the publisher, the editors and the reviewers. Any product that may be evaluated in this article, or claim that may be made by its manufacturer, is not guaranteed or endorsed by the publisher.
