# Supplementary material for: Interaction Analysis of Adenovirus L5 Protein With Pancreatic Cancer Cell Surface Receptor to Analyze Its Affinity for Oncolytic Virus Therapy
Source: Front Oncol. 2022 Mar 10;12:832277. doi: 10.3389/fonc.2022.832277 (PMC8960272; doi:10.3389/fonc.2022.832277)
Supplement: Supplementary file 1 [file DataSheet_1.pdf]

Supplementary Material

Table (S1). Active site residues of HAdV2, HAdV3 and HAdV5 predicted by CPORT.

Sr. No	Serotype Name of L5 protein	Active site residues
1	HAdV2	Chain A: 407, 416, 474, 475, 476, 477<br>Chain B: 408, 409, 410, 418, 467, 475, 476, 477, 478, 490, 491<br>Chain C: 408, 409, 410, 476, 477, 478, 479, 487
2	HAdV3	Chain A: 144, 145, 146, 147, 148, 149, 150, 151, 177, 179, 192, 194, 206, 207, 252, 253, 270, 289<br>Chain B: 146, 147, 148, 193, 195, 197, 253, 271, 288<br>Chain C: 146, 147, 148, 149, 150, 151, 152, 153, 154, 179, 181, 194, 196, 208, 209, 252, 254, 255, 272, 274, 285, 289, 311, 313
3	HAdV5	Chain A: 396, 397, 398, 399, 406, 458, 460, 467, 468, 470, 471, 472, 474, 476, 477, 481, 482, 483, 484, 485, 486, 487, 488, 489, 490, 491<br>Chain B: 430<br>Chain C: 396, 397

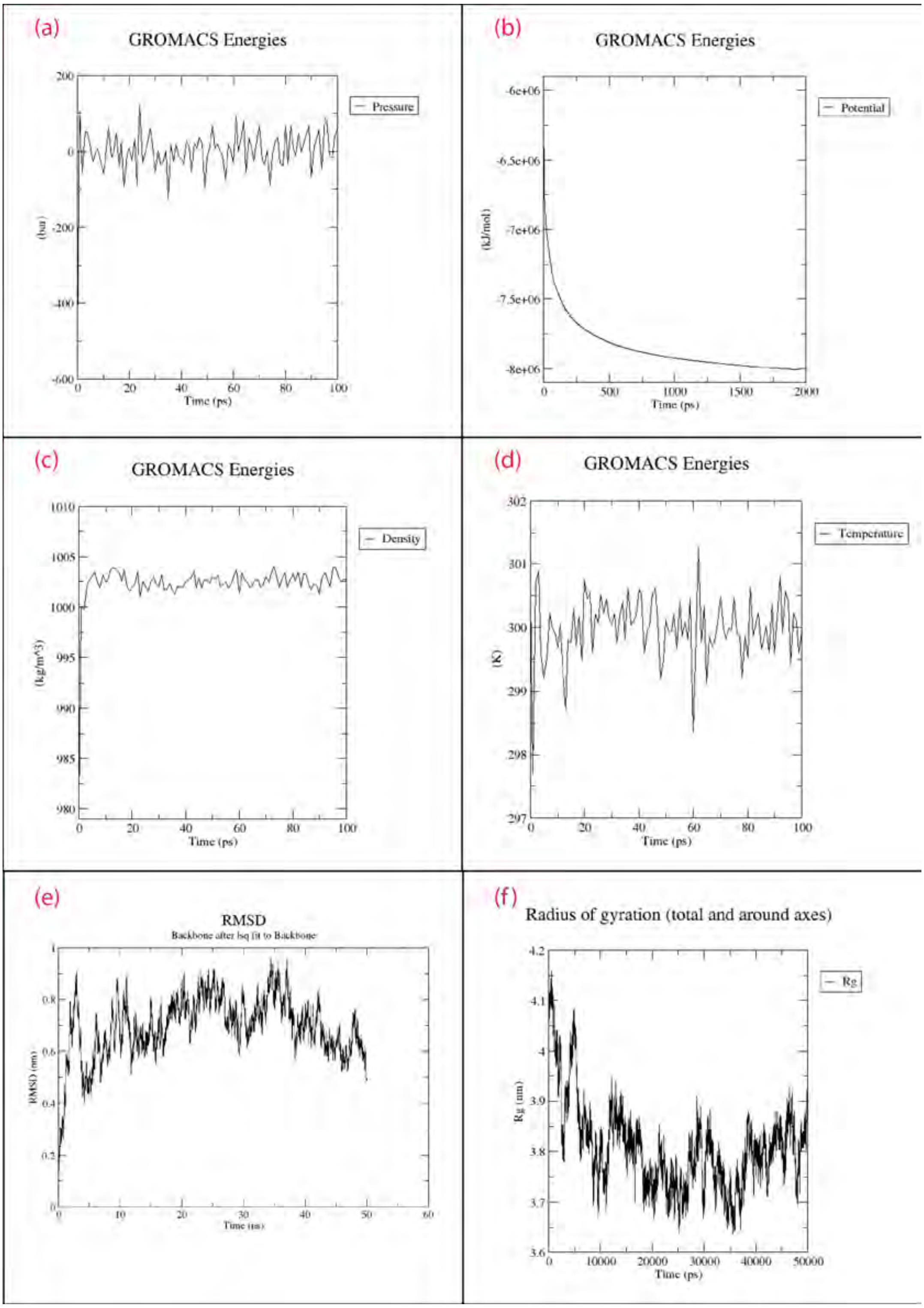

Figure (S1) **Molecular Dynamic (MD) simulation plots of GABRP receptor:** (a) Plot represent pressure equilibration step NPT (b) Potential Energy plot. Represents the steady decrease in potential energy. Native protein reached its minimized state on 2000 ps. (c) Plot represents density equilibration step NPT (d) Plot of temperature equilibration step NVT on 300k temperature (e) RMSD plot shows protein backbone energy minimization by 50ns MD simulation (f) Radius of gyration plot. The compactness of protein stabilizes around 3.7 to 3.9 Rg(nm) from time steps 18000ps to 50000ps.

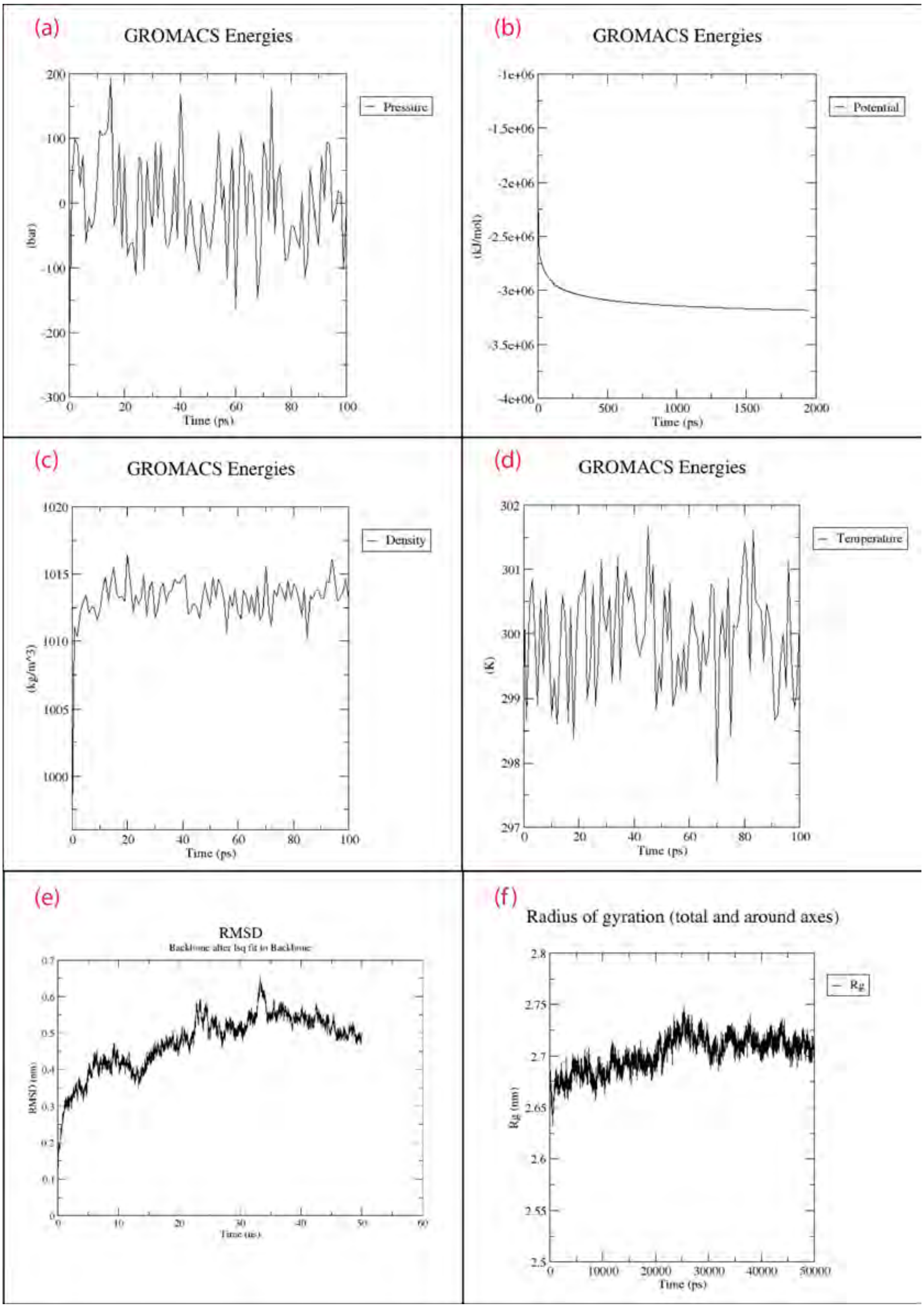

Figure (S2) **Molecular Dynamic (MD) simulation plots of SLC6A6 receptor:** (a) Plot represent pressure equilibration step NPT (b) Potential Energy plot. Represents the steady decrease in potential energy. Native protein reached its minimized state on 2000 ps. (c) Plot represents density equilibration step NPT (d) Plot of temperature equilibration step NVT on 300k temperature (e) RMSD plot shows protein backbone energy minimization by 50ns MD simulation (f) Radius of gyration plot. The compactness of protein stabilizes around 2.7 to 2.75 Rg(nm) from time steps 20000ps to 50000ps.

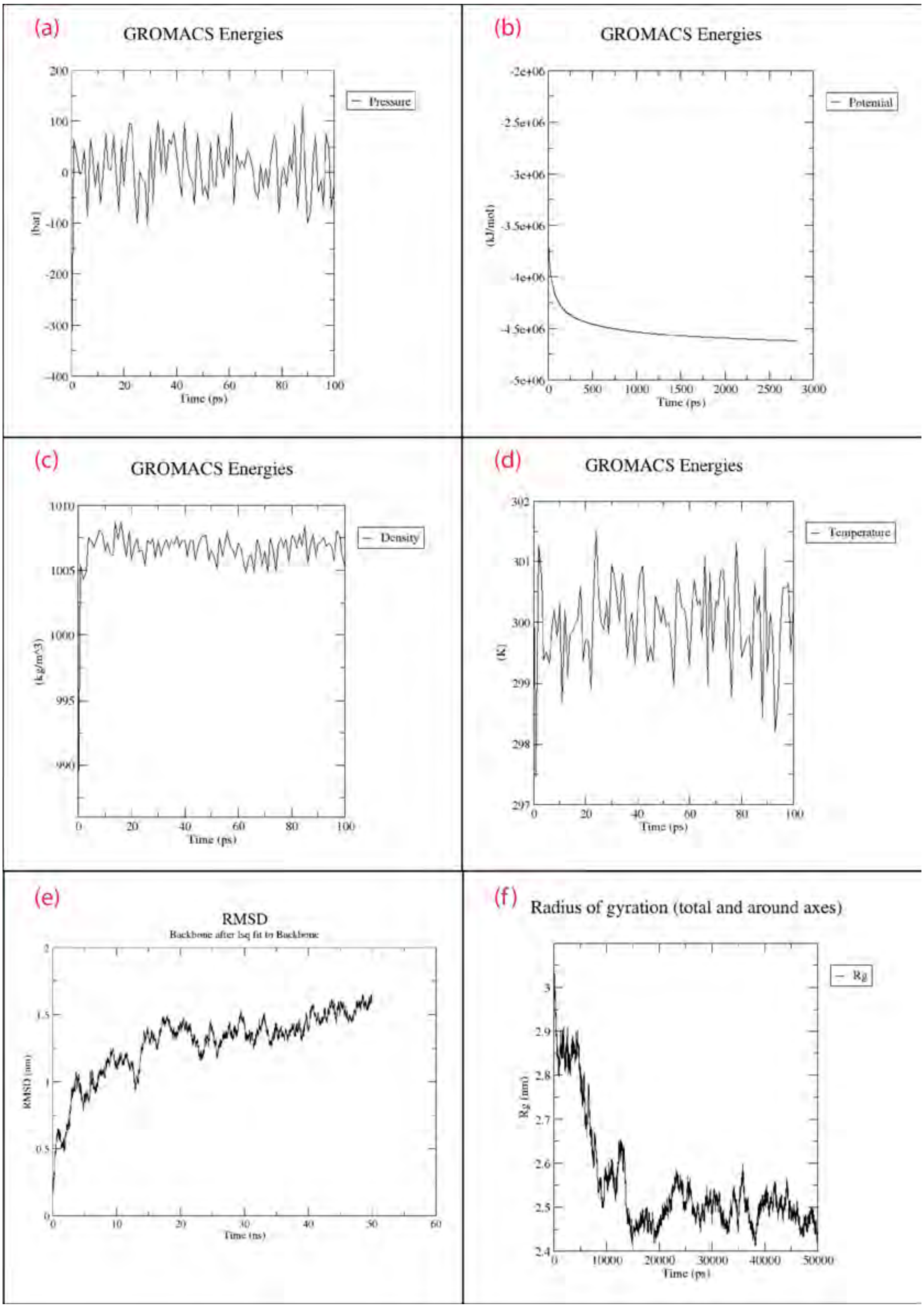

Figure (S3) **Molecular Dynamic (MD) simulation plots of GABRP receptor:** (a) Plot represent pressure equilibration step NPT (b) Potential Energy plot. Represents the steady decrease in potential energy. Native protein reached its minimized state on 3000 ps. (c) Plot represents density equilibration step NPT (d) Plot of temperature equilibration step NVT on 300k temperature (e) RMSD plot shows protein backbone energy minimization by 50ns MD simulation (f) Radius of gyration plot. The compactness of protein stabilizes around 2.4 to 2.6 Rg(nm) from time steps 15000ps to 50000ps.

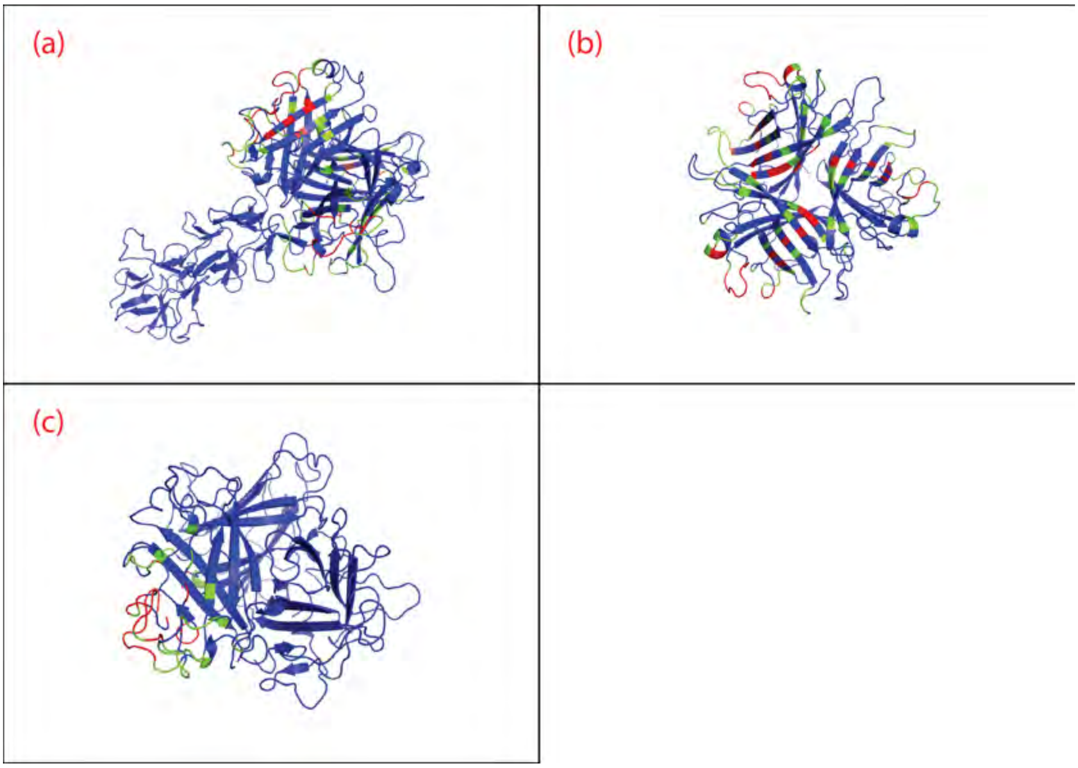

Figure (S4) **CPORT active and passive residues representation of Human adenovirus L5 protein. The list of active residues is provided in Table S1.** (a) The cartoon representation of HAdV2 L5 protein red color ribbon show active residues and green color represent passive residue predicted by CPORT. (b) The cartoon representation active and passive residues of HAdV3 L5 protein. (c) The cartoon representation active and passive residues of HAdV5 L5 protein predicted by CPORT.

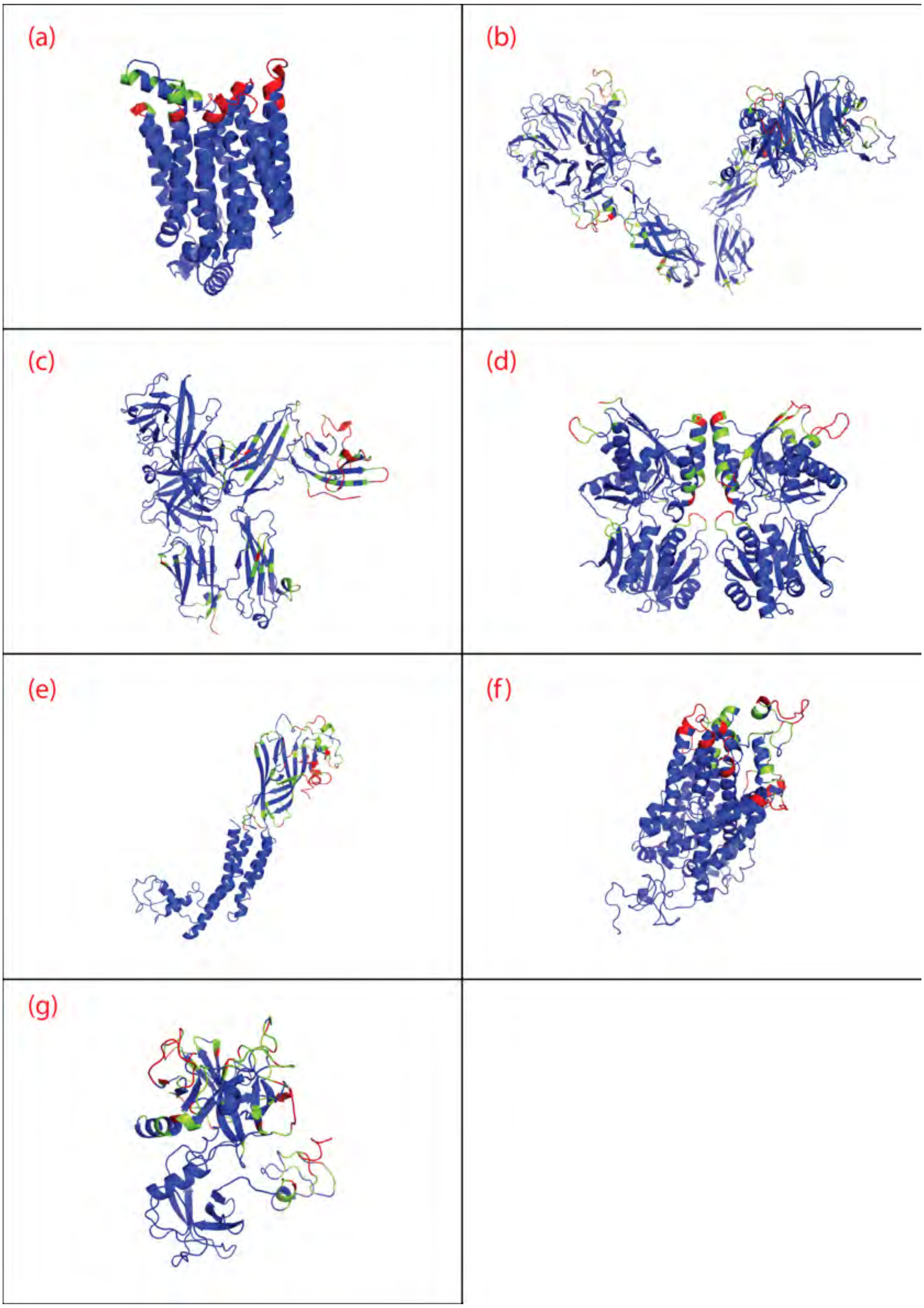

Figure (S5) **CPORT active and passive residues representation of pancreatic cancer receptors. The list of active residues for all seven receptors is provided in Table S2.** (a) The cartoon representation of SLC2A1 red color ribbon show active residues and green color represent passive residue predicted by CPORT. (b) The cartoon representation active and passive residues of MET receptor. (c) The cartoon representation active and passive residues of IL1RAP receptor predicted by CPORT. (d) The cartoon representation active and passive residues of NPR3 receptor predicted by CPORT. (e) The cartoon representation active and passive residues of GABRP receptor predicted by CPORT. (f) The cartoon representation active and passive residues of SLC6A6 receptor predicted by CPORT. (g) The cartoon representation active and passive residues of TMPRSS4 receptor predicted by CPORT.

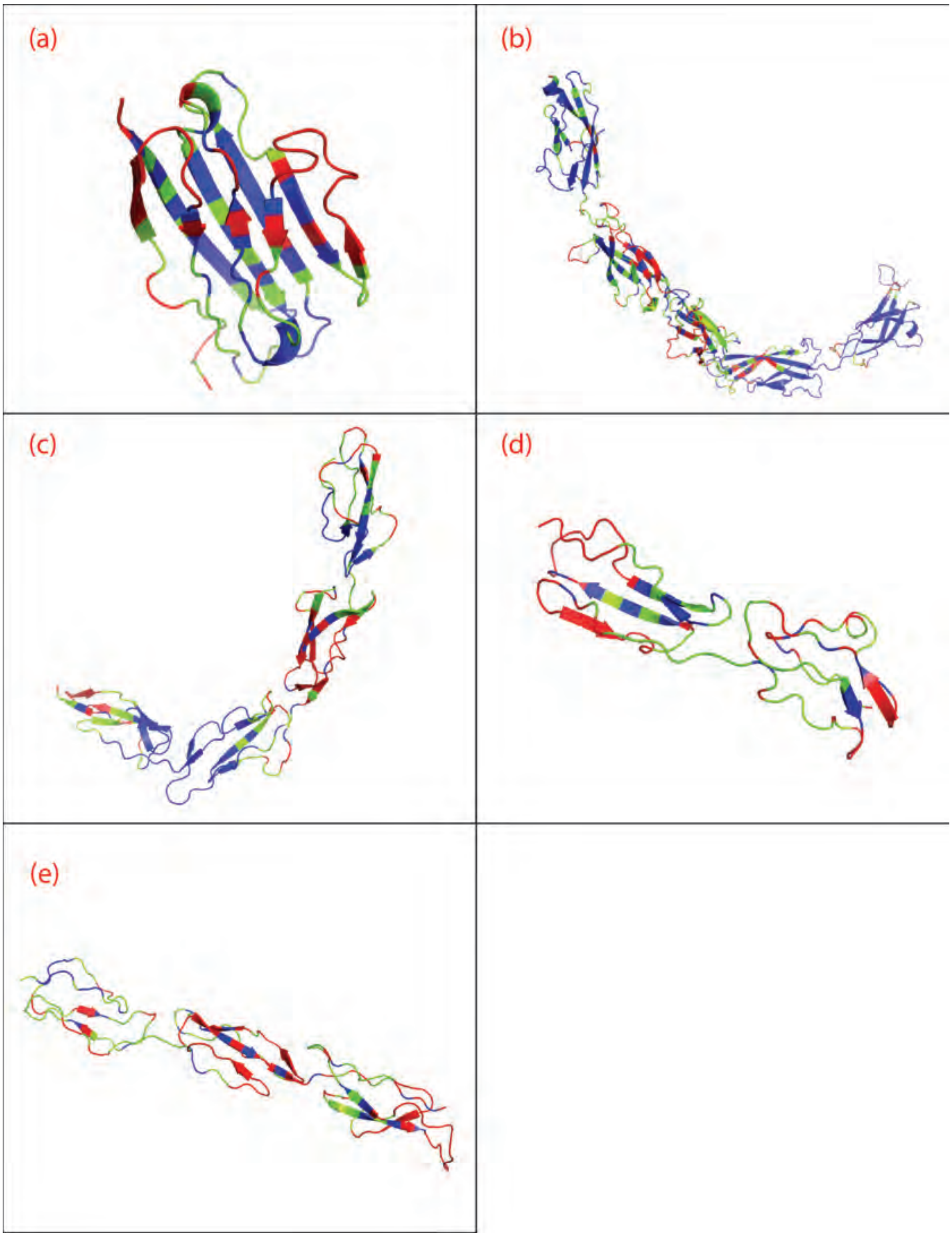

Figure (S6) **CPORT active and passive residues representation of Human receptors for positive control. The list of active residues is provided in Table S3.** (a) The cartoon representation of CAR, red color ribbon show active residues and green color represent passive residue predicted by CPORT. (b) The cartoon representation active and passive residues of DSG2. (c)The representation of CD46 active and passive residues. (d) The cartoon representation active and passive residues of CR1 sushi domain 1 and 2 (PDB id: 2MCZ) predicted by CPORT. (e) The cartoon representation active and passive residues of CR1 sushi domain 15, 16, and 17 (PDB id: 5FO9) predicted by CPORT.

Table (S2). Active site residues of receptors predicted by CPORT.

Sr. No	Receptor Name	Active site residues
1	SLC2A1	115, 116, 120, 177, 178, 179, 183, 184, 185, 300, 302, 304, 305, 356, 357, 358, 359, 360, 361, 362, 363, 424, 425, 427, 428, 429
2	MET	Chain A: 40, 98, 137, 140, 162, 391, 394, 395, 396, 397, 398, 399, 401, 402, 403, 404, 405, 408, 415, 523, 524, 525, 526, 528, 540, 543, 549, 552, 554, 555, 586, 607, 740<br>Chain B: 104, 141, 385, 392, 393, 394, 395, 396, 397, 398, 399, 400, 401, 402, 403, 404, 405, 406, 416, 526, 527, 541, 543, 544, 553, 554, 555, 556, 587, 700, 703
3	IL1RAP	Chain A: -<br>Chain B: 172, 281, 329, 330, 331<br>Chain C: 25, 26, 27, 28, 29, 31, 32, 40, 51, 52, 55, 56, 57, 58, 60, 61, 63, 64, 71, 79, 80, 82, 83, 84, 85, 86, 87, 88, 89, 91, 92, 93, 102, 103, 119, 120, 121, 136, 137, 138, 178, 180, 181, 182, 183, 290, 340
4	NPR3	Chain A: 47, 87, 88, 89, 109, 122, 138, 209, 210<br>Chain B: 47, 48, 49, 50, 52, 84, 85, 86, 87, 88, 89, 90, 91, 92, 95, 96, 98, 107, 109, 122, 138, 209, 210
5	GABRP	1, 2, 3, 4, 5, 6, 7, 8, 9, 10, 11, 12, 13, 15, 16, 17, 18, 19, 20, 21, 22, 26, 30, 33, 34, 46, 47, 48, 51, 96, 110, 119, 121, 130, 131, 132, 133, 134, 135, 138, 139, 166, 182, 208, 223, 226, 241, 242
6	SLC6A6	146, 150, 151, 153, 154, 155, 156, 177, 178, 180, 181, 182, 183, 184, 185, 186, 187, 188, 189, 190, 191, 208, 211, 212, 213, 214, 215, 216, 217
7	TMPRSS4	54, 55, 56, 57, 58, 59, 61, 62, 70, 95, 204, 205, 206, 207, 208, 209, 210, 229, 245, 248, 249, 251, 253, 254, 276, 277, 278, 280, 281, 282, 283, 284, 285, 286, 287, 288, 298, 302, 303, 323, 332, 338, 369, 380, 385, 387, 406, 411, 425

Table (S3). Active site residues of CAR, DSG2, CD46 and CR1 predicted by CPORT.

Sr. No	Receptor name	Active site residues
1	CAR	16, 18, 19, 23, 24, 28, 29, 30, 31, 32, 33, 34, 35, 36, 38, 56, 70, 73, 79, 80, 81, 82, 83, 84, 85, 86, 87, 88, 89, 90, 92, 94, 106, 108, 109, 112, 138, 139
2	DSG2	97, 99, 101, 105, 110, 112, 113, 114, 116, 117, 118, 124, 125, 126, 127, 128, 129, 131, 133, 137, 139, 140, 141, 143, 146, 147, 158, 167, 168, 170, 172, 174, 175, 176, 179, 199, 200, 201, 202, 203, 206, 210, 222, 223, 224, 225, 226, 227, 228, 231, 238, 239, 240, 242, 243, 245, 247, 260, 284, 288, 304, 313, 314, 316, 317, 318, 320, 321, 322, 323, 324, 325, 327, 329, 330, 333, 334
3	CD46	14, 15, 18, 19, 21, 22, 23, 38, 39, 41, 45, 48, 49, 66, 67, 69, 70, 71, 72, 73, 74, 75, 76, 77, 79, 81, 82, 83, 88, 90, 92, 93, 94, 95, 97, 98, 99, 100, 101, 102, 103, 104, 106, 111, 115, 116, 117, 118, 119, 120, 122, 123, 125, 141, 144, 145, 146, 149, 150, 179, 205, 208, 221, 222, 224, 225, 226, 227, 228, 229, 248, 252
4	CR1 receptor sushi domain 1 and 2 (2MCZ)	1, 7, 9, 10, 12, 14, 17, 23, 24, 25, 26, 27, 29, 32, 33, 37, 46, 47, 48, 49, 51, 56, 66, 68, 70, 71, 72, 73, 74, 75, 76, 77, 78, 79, 80, 83, 89, 91, 92, 93, 94, 95, 96, 97, 98, 99, 100, 101, 117, 118, 119, 120, 121, 122
5	CR1 receptor sushi domain 15, 16, and 17 (5FO9)	955, 956, 965, 966, 969, 970, 975, 976, 983, 993, 994, 996, 997, 1009, 1010, 1011, 1012, 1013, 1014, 1015, 1016, 1017, 1018, 1019, 1020, 1022, 1023, 1024, 1025, 1026, 1028, 1030, 1031, 1033, 1034, 1035, 1036, 1037, 1038, 1039, 1040, 1042, 1044, 1058, 1060, 1062, 1063, 1065, 1067, 1068, 1071, 1072, 1073, 1074, 1075, 1076, 1077, 1078, 1079, 1080, 1083, 1084, 1086, 1087, 1094, 1096, 1097, 1098, 1099, 1100, 1101, 1102, 1103, 1104, 1105, 1106, 1107, 1108, 1109, 1110, 1112, 1118, 1122, 1129, 1131, 1133

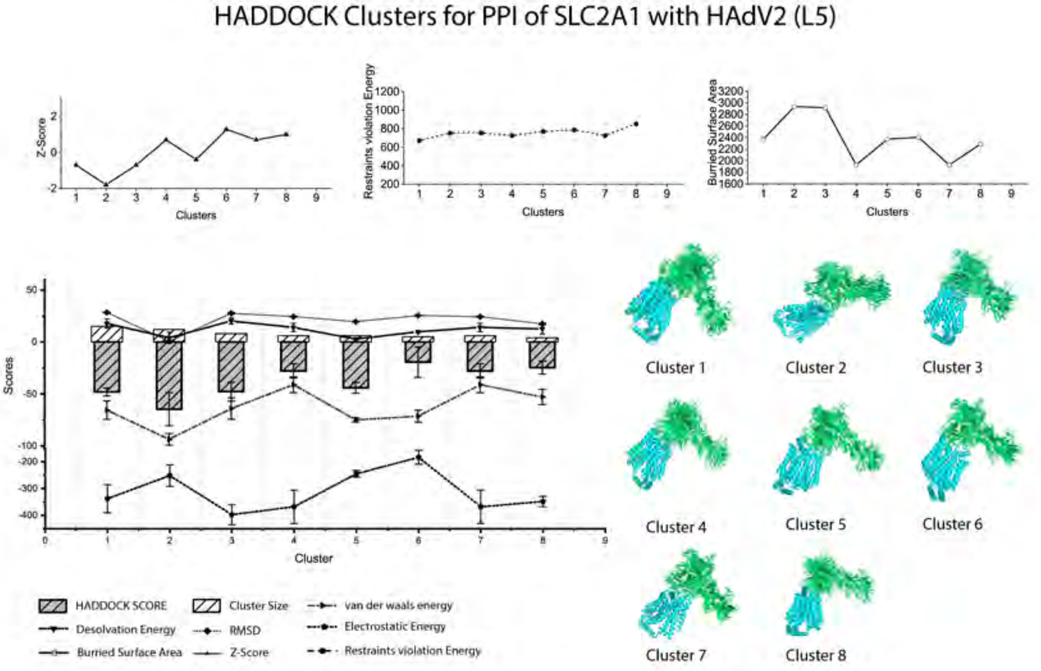

Figure (S7) **Protein-protein interaction complex of HAdV2 L5 protein with SLC2A1 receptor:** HADDOCK run generate 8 clusters, superimposed structures of all clusters and plots of HADDOCK scores, Cluster size, RMSD, Van der Waals energy, Electrostatic energy, Desolvation energy, Restraints violation energy, Buried Surface Area, and Z-Score are given above. The cluster 2 with HADDOCK score of -64.6 +/- 16.0 and lowest Z-score of -1.8 is the best cluster selected for the further analysis.

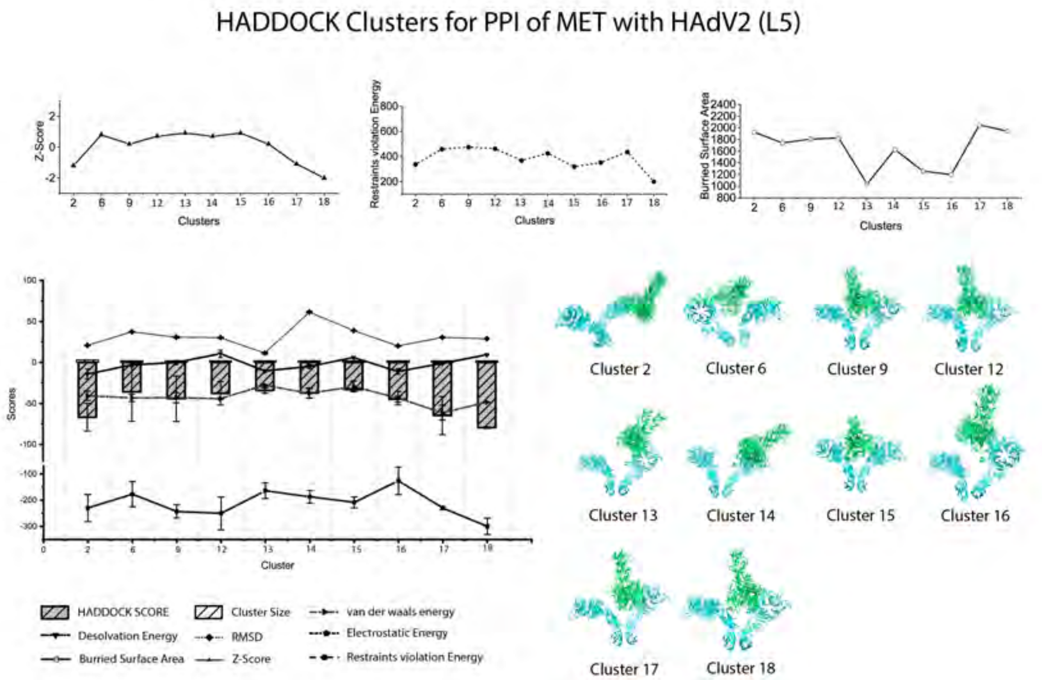

Figure (S8) **Protein-protein interaction complex of HAdV2 L5 protein with MET receptor:** HADDOCK run generate 18 clusters, superimposed structures of top 10 clusters and plots of HADDOCK scores, Cluster size, RMSD, Van der Waals energy, Electrostatic energy, Desolvation energy, Restraints violation energy, Buried Surface Area, and Z-Score are given above. The cluster 18 with HADDOCK score of -79.5 +/- 1.1 and lowest Z-score of -2.0 is the best cluster selected for the further analysis.

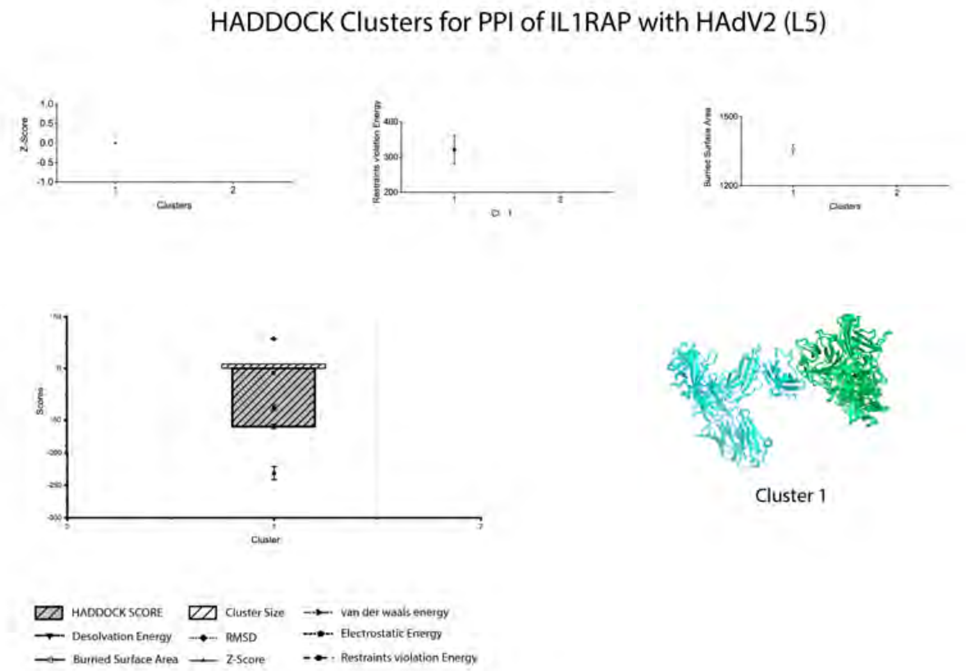

Figure (S9) **Protein-protein interaction complex of HAdV2 L5 protein with IL1RAP receptor:** HADDOCK run generate 1 clusters, superimposed structures of cluster and plots of HADDOCK scores, Cluster size, RMSD, Van der Waals energy, Electrostatic energy, Desolvation energy, Restraints violation energy, Buried Surface Area, and Z-Score are given above.

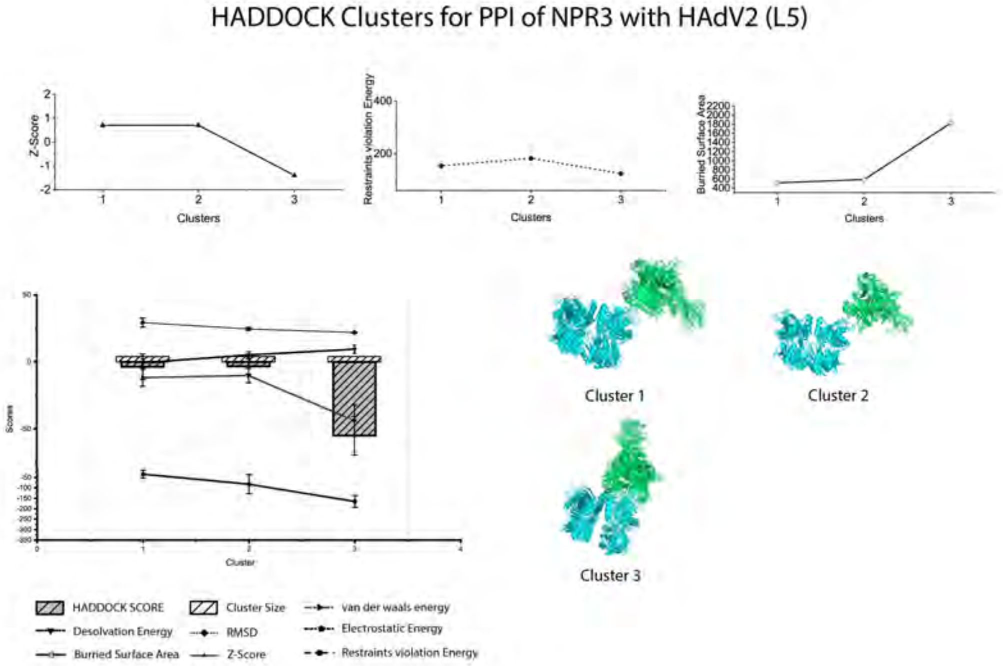

Figure (S10) **Protein-protein interaction complex of HAdV2 L5 protein with NPR3 receptor:** HADDOCK run generate 3 clusters, superimposed structures of top all clusters and plots of HADDOCK scores, Cluster size, RMSD, Van der Waals energy, Electrostatic energy, Desolvation energy, Restraints violation energy, Buried Surface Area, and Z-Score are given above. The cluster 3 is the top interaction complex with HADDOCK score of -55.0 +/- 14.4 and lowest Z-score of -1.4 selected for further analysis.

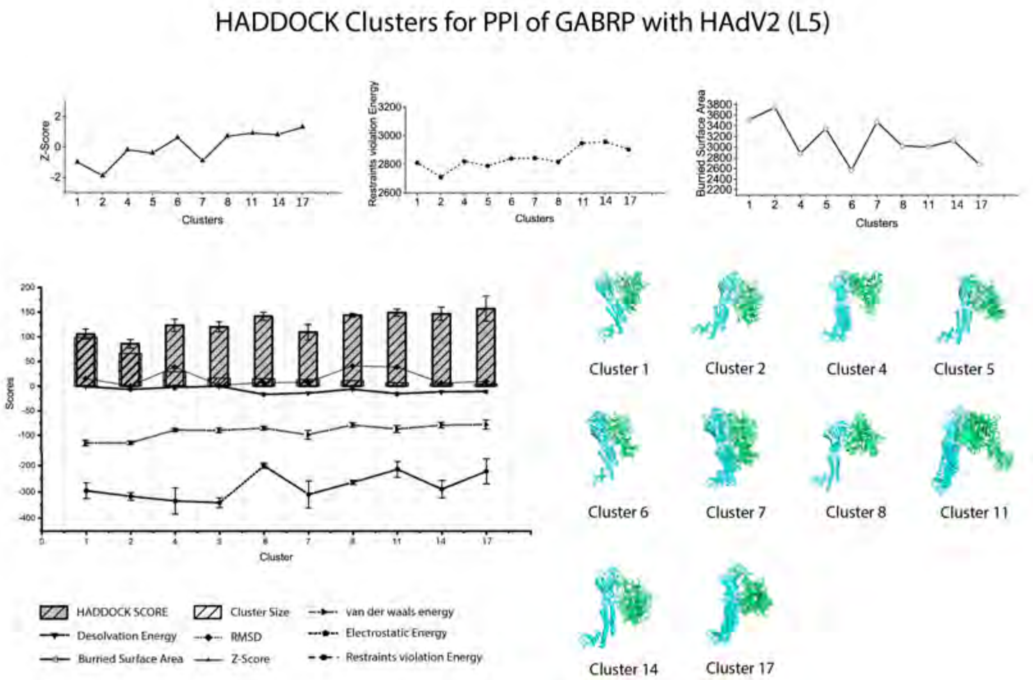

Figure (S11) **Protein-protein interaction complex of HAdV2 L5 protein with GABRP receptor:** HADDOCK run generate 17 clusters, superimposed structures of top 10 clusters and plots of HADDOCK scores, Cluster size, RMSD, Van der Waals energy, Electrostatic energy, Desolvation energy, Restraints violation energy, Buried Surface Area, and Z-Score are given above. The cluster 2 with HADDOCK score of 85.9 +/- 8.6 and lowest Z-score of -1.9 is the best cluster selected for the further analysis.

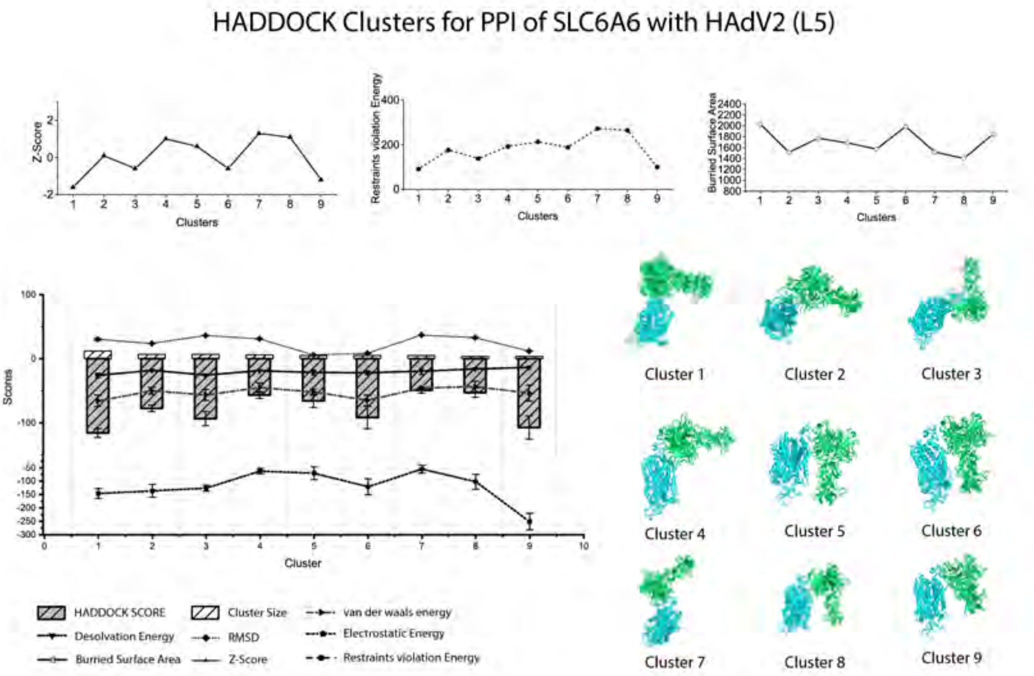

Figure (S12) **Protein-protein interaction complex of HAdV2 L5 protein with SLC6A6 receptor:** HADDOCK run generate 9 clusters, superimposed structures of all clusters and plots of HADDOCK scores, Cluster size, RMSD, Van der Waals energy, Electrostatic energy, Desolvation energy, Restraints violation energy, Buried Surface Area, and Z-Score are given above. The cluster 1 is the best possible interaction complex for SLC6A6 and HAdV2 with HADDOCK score of -115.8 +/- 6.9 and lowest Z-score of -1.6 selected for the further analysis.

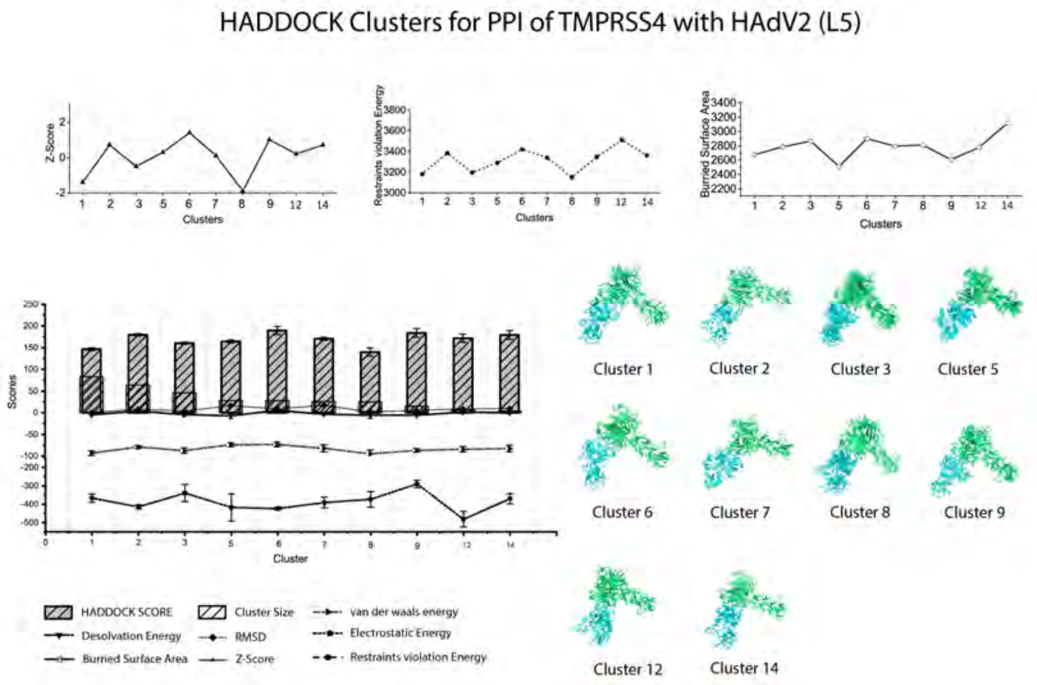

Figure (S13) **Protein-protein interaction complex of HAdV2 L5 protein with TMPRSS4 receptor:** HADDOCK run generate 8 clusters, superimposed structures of all clusters and plots of HADDOCK scores, Cluster size, RMSD, Van der Waals energy, Electrostatic energy, Desolvation energy, Restraints violation energy, Buried Surface Area, and Z-Score are given above. The cluster 8 is the best possible interaction complex for TMPRSS4 and HAdV2 L5 protein with HADDOCK score of 139.9 +/- 9.5 and Z-score of -1.9 selected for the further analysis.

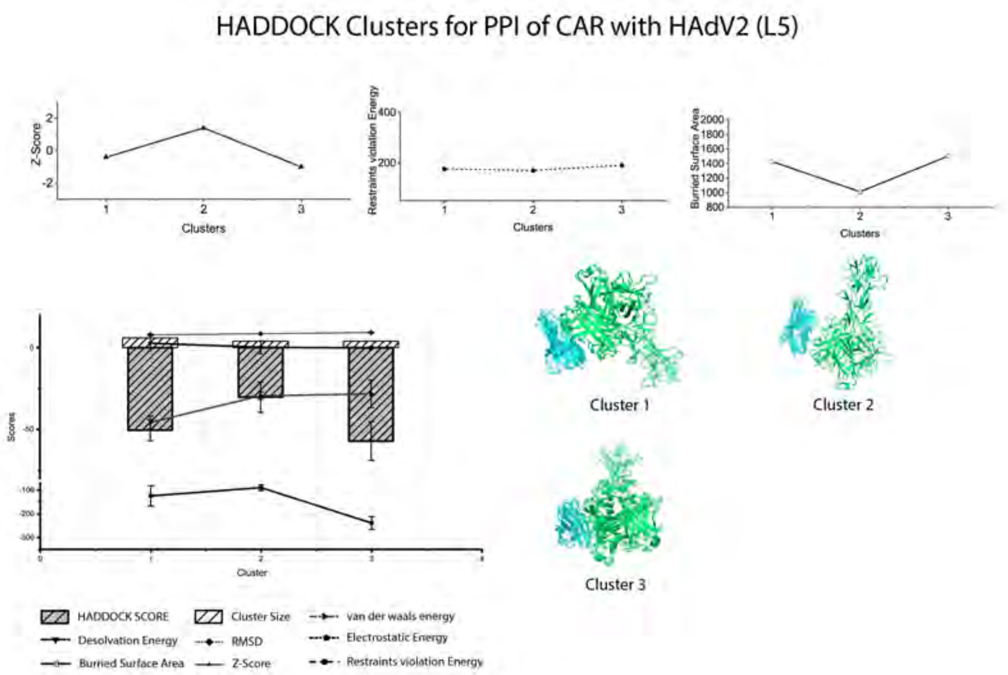

Figure (S14) **Protein-protein interaction complex of HAdV2 L5 protein with CAR receptor:** HADDOCK run generate 3 clusters, superimposed structures of all clusters and plots of HADDOCK scores, Cluster size, RMSD, Van der Waals energy, Electrostatic energy, Desolvation energy, Restraints violation energy, Buried Surface Area, and Z-Score are given above. The cluster 3 is the best possible interaction complex for CAR and HAdV2 L5 protein with HADDOCK score of -57.2 +/- 11.9 and Z-score of -1.0 selected for the further analysis.

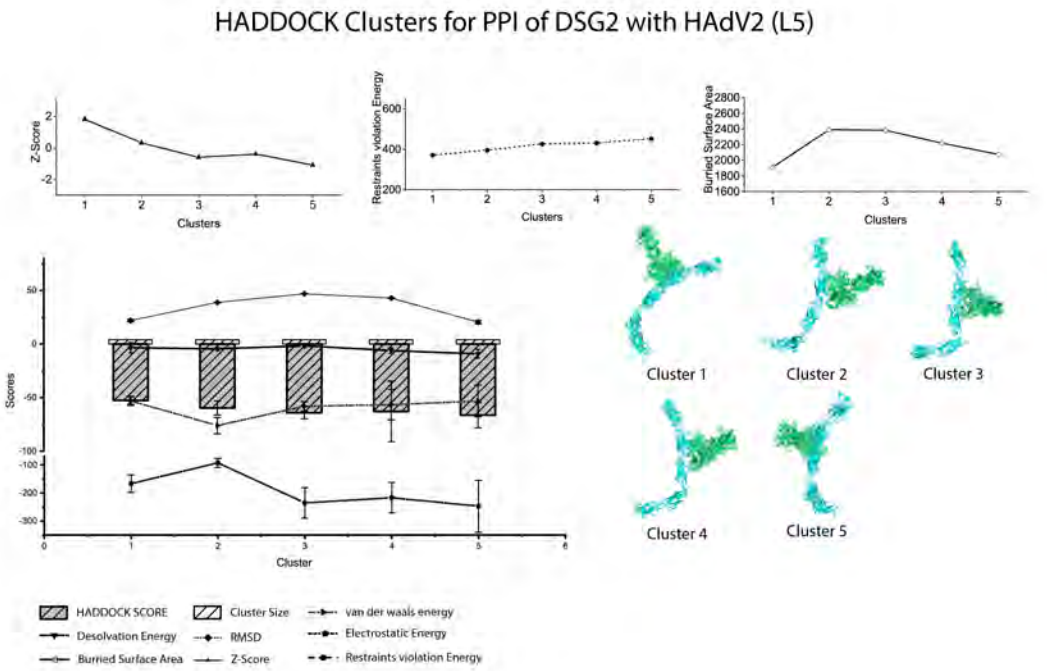

Figure (S15) **Protein-protein interaction complex of HAdV2 L5 protein with DSG2 receptor:** HADDOCK run generate 5 clusters, superimposed structures of all clusters and plots of HADDOCK scores, Cluster size, RMSD, Van der Waals energy, Electrostatic energy, Desolvation energy, Restraints violation energy, Buried Surface Area, and Z-Score are given above. The cluster 5 is the best possible interaction complex for DSG2 and HAdV2 L5 protein with HADDOCK score of -66.5 +/- 11.8 and Z-score of -1.1 selected for the further analysis.

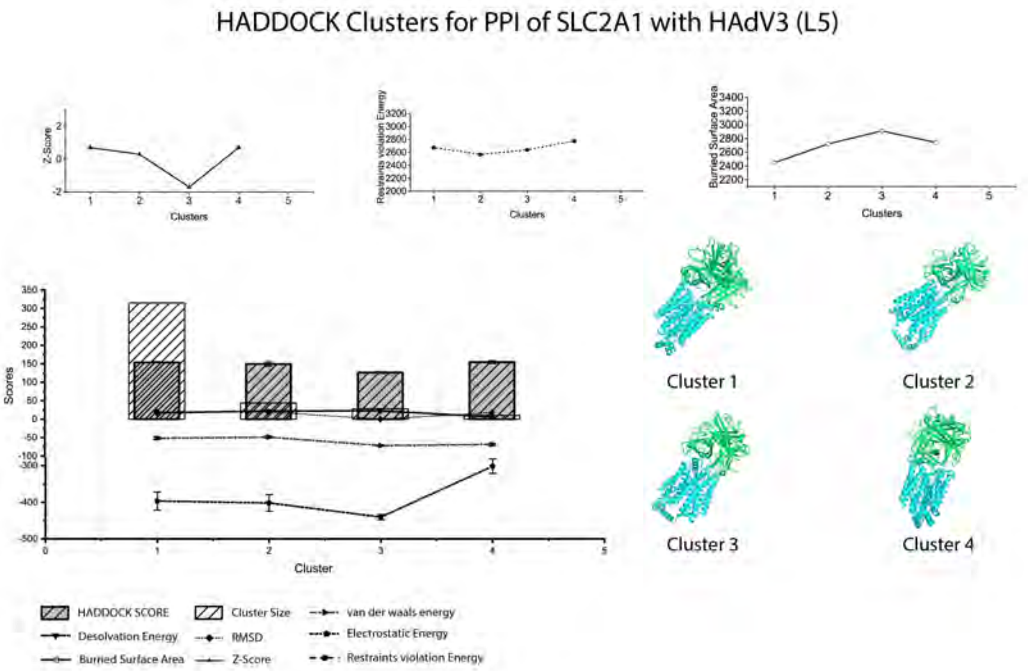

Figure (S16) **Protein-protein interaction complex of HAdV3 L5 protein with SLC2A1 receptor:** HADDOCK run generate 4 clusters, superimposed structures of all clusters and plots of HADDOCK scores, Cluster size, RMSD, Van der Waals energy, Electrostatic energy, Desolvation energy, Restraints violation energy, Buried Surface Area, and Z-Score are given above. The cluster 3 is the top interaction complex with HADDOCK score of 126.4 +/- 2.4 and lowest Z-score of -1.7 selected for the further analysis.

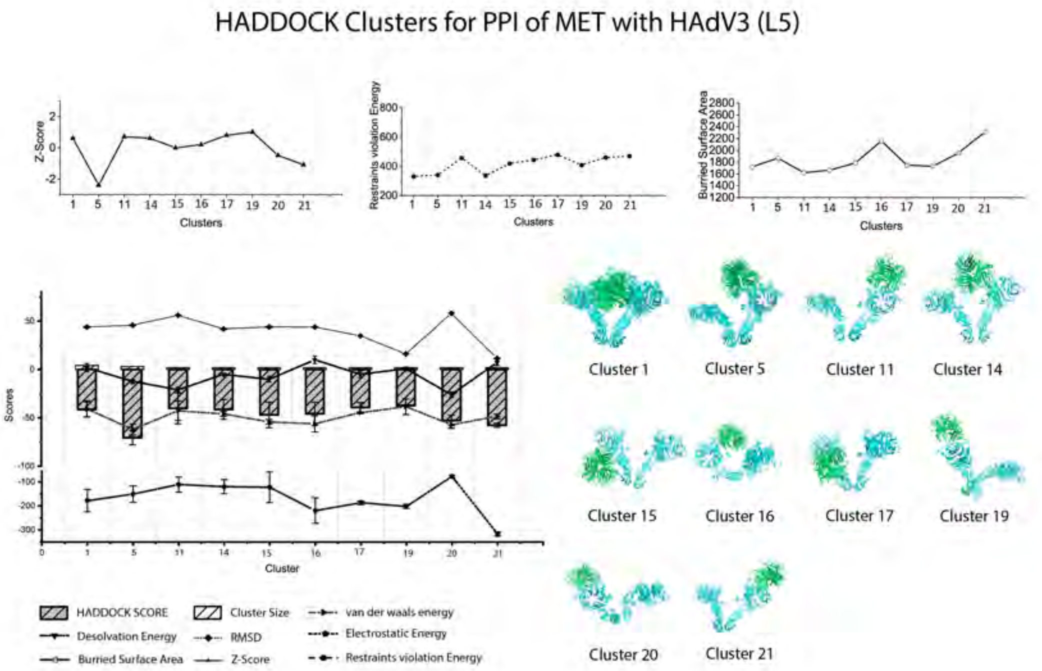

Figure (S17) **Protein-protein interaction complex of HAdV3 L5 protein with MET receptor:** HADDOCK run generate 21 clusters, superimposed structures of top 10 clusters and plots of HADDOCK scores, Cluster size, RMSD, Van der Waals energy, Electrostatic energy, Desolvation energy, Restraints violation energy, Buried Surface Area, and Z-Score are given above. The cluster 5 with HADDOCK score of -70.6 +/- 7.2 and lowest Z-score of -2.4 selected for the further analysis.

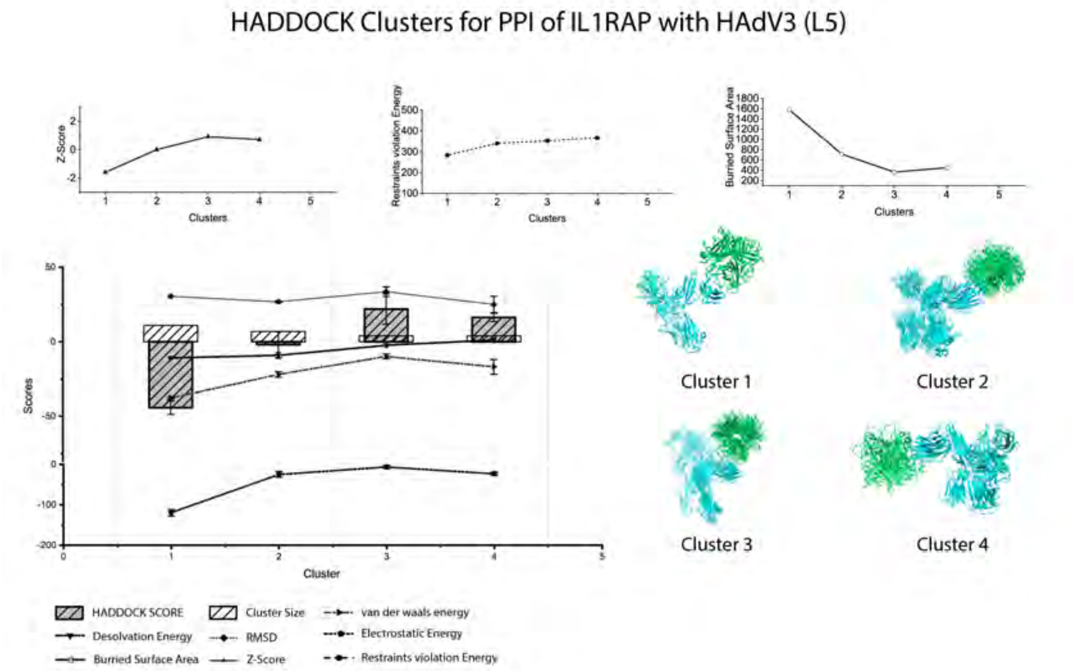

Figure (S18) **Protein-protein interaction complex of HAdV3 L5 protein with IL1RAP receptor:** HADDOCK run generate 4 clusters, superimposed structures of all clusters and plots of HADDOCK scores, Cluster size, RMSD, Van der Waals energy, Electrostatic energy, Desolvation energy, Restraints violation energy, Buried Surface Area, and Z-Score are given above. The cluster 4 is the top interaction complex with HADDOCK score of -44.2 +/- 4.5 and lowest Z-score of -1.6 selected for the further analysis.

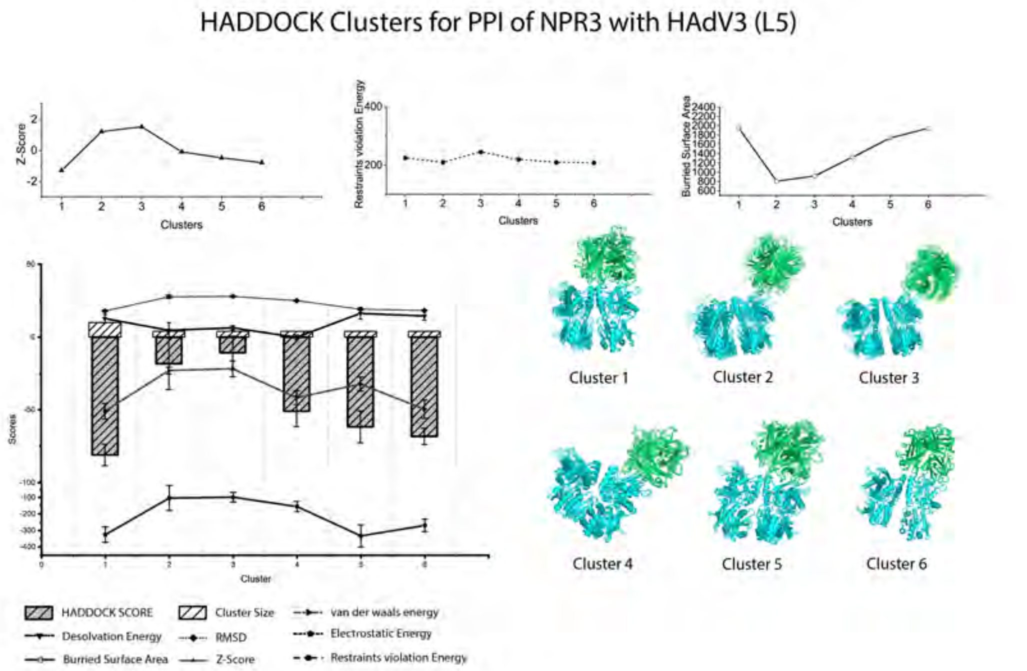

Figure (S19) **Protein-protein interaction complex of HAdV3 L5 protein with NPR3 receptor:** HADDOCK run generate 6 clusters, superimposed structures of all clusters and plots of HADDOCK scores, Cluster size, RMSD, Van der Waals energy, Electrostatic energy, Desolvation energy, Restraints violation energy, Buried Surface Area, and Z-Score are given above. The cluster 1 is the top interaction complex with HADDOCK score of -81.0 +/- 7.4 and lowest Z-score of -1.3 selected for the further analysis.

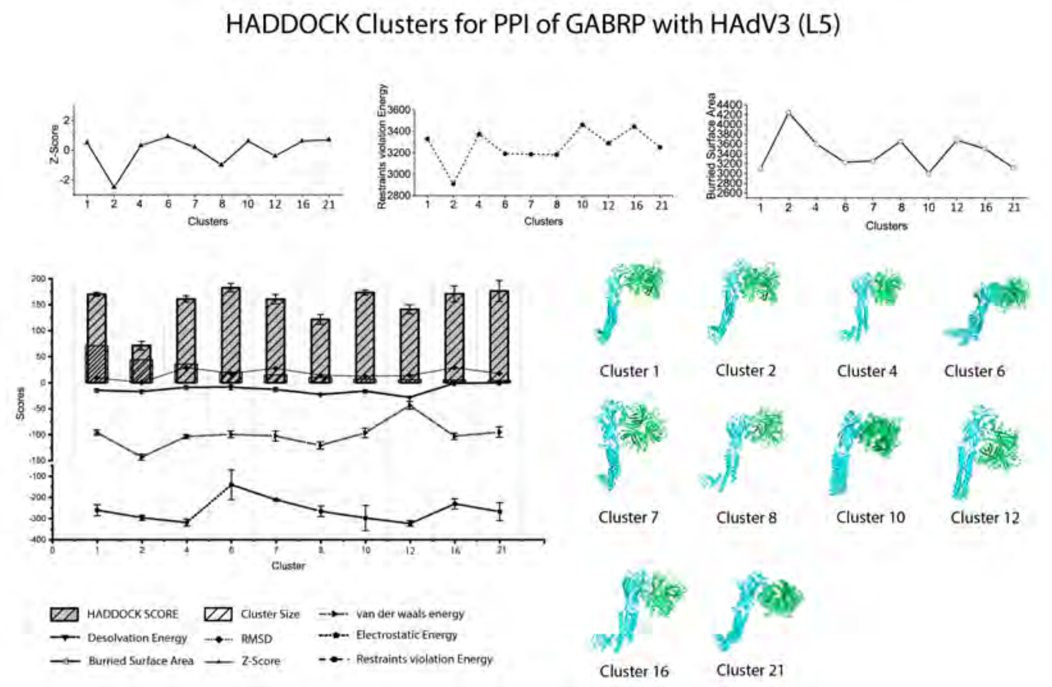

Figure (S20) **Protein-protein interaction complex of HAdV3 L5 protein with GABRP receptor:** HADDOCK run generate 22 clusters, superimposed structures of top 10 clusters and plots of HADDOCK scores, Cluster size, RMSD, Van der Waals energy, Electrostatic energy, Desolvation energy, Restraints violation energy, Buried Surface Area, and Z-Score are given above. The cluster 2 is top interacting complex with HADDOCK score of 71.4 +/- 8.2 and lowest Z-score of -2.5 selected for the further analysis.

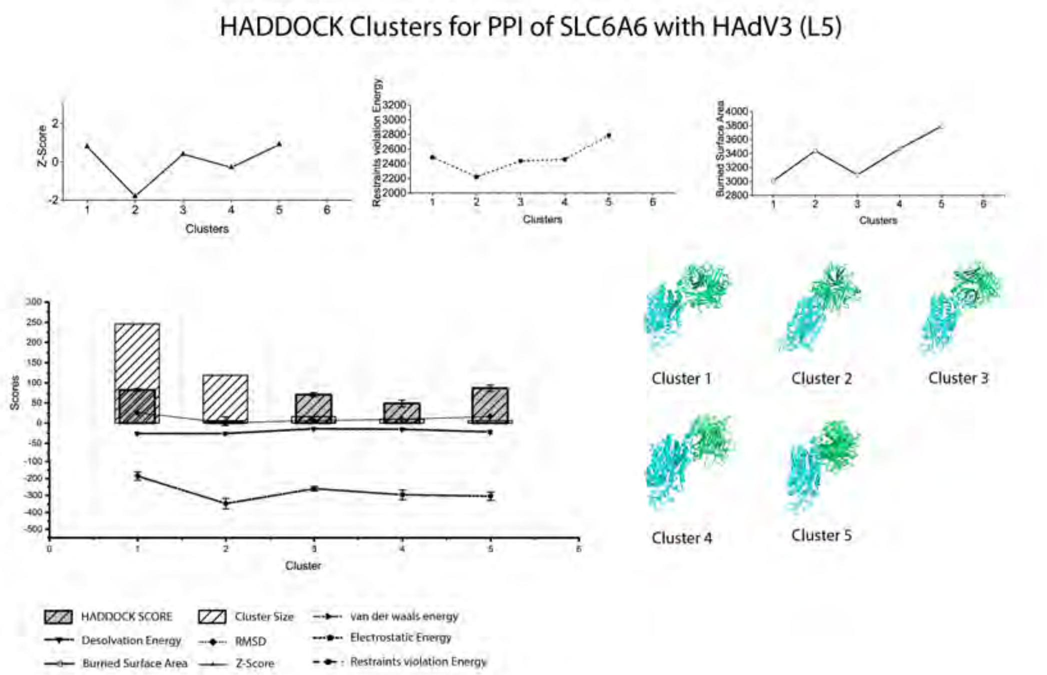

Figure (S21) **Protein-protein interaction complex of HAdV3 L5 protein with SLC6A6 receptor:** HADDOCK run generate 5 clusters, superimposed structures of all clusters and plots of HADDOCK scores, Cluster size, RMSD, Van der Waals energy, Electrostatic energy, Desolvation energy, Restraints violation energy, Buried Surface Area, and Z-Score are given above. The cluster 2 is top cluster with HADDOCK score of 4.8 +/- 10.8 and lowest Z-score of -1.8 selected for the further analysis.

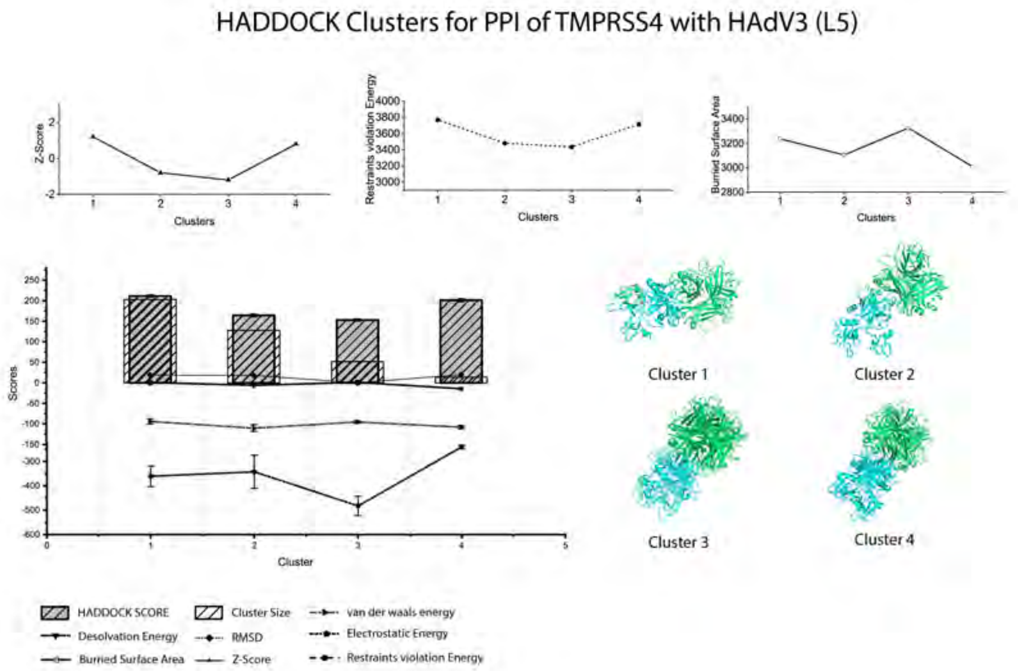

Figure (S22) **Protein-protein interaction complex of HAdV3 L5 protein with TMPRSS4 receptor:** HADDOCK run generate 4 clusters, superimposed structures of all clusters and plots of HADDOCK scores, Cluster size, RMSD, Van der Waals energy, Electrostatic energy, Desolvation energy, Restraints violation energy, Buried Surface Area, and Z-Score are given above. The cluster 3 is top interacting complex with HADDOCK score of 153.1 +/- 2.7 and lowest Z-score of -1.2 selected for the further analysis.

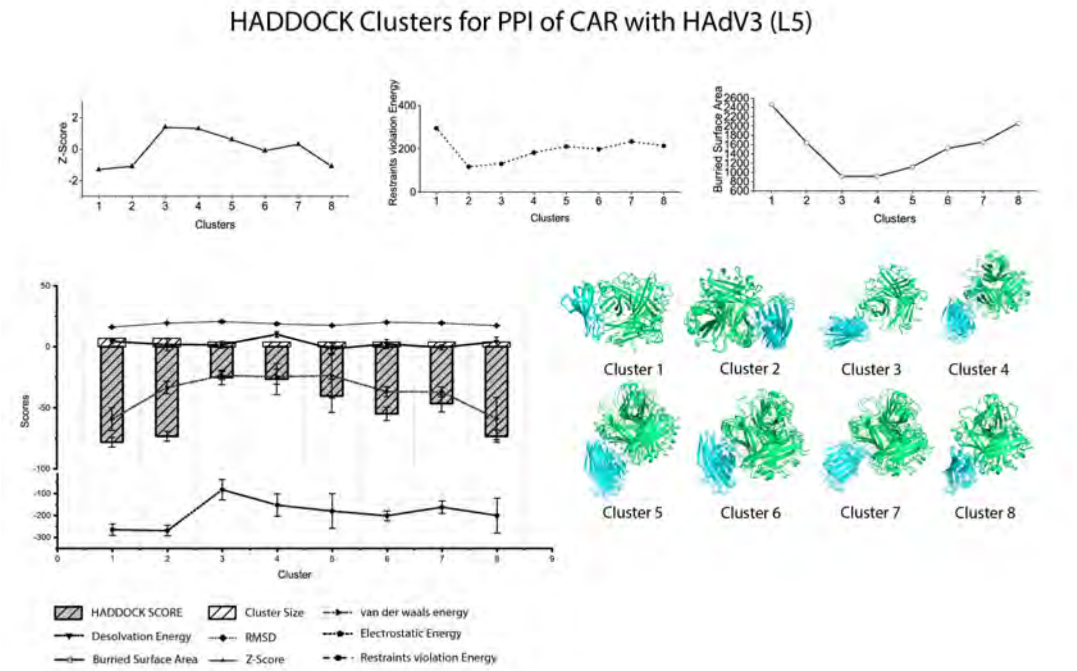

Figure (S23) **Protein-protein interaction complex of HAdV3 L5 protein with CAR receptor:** HADDOCK run generate 8 clusters, superimposed structures of all clusters and plots of HADDOCK scores, Cluster size, RMSD, Van der Waals energy, Electrostatic energy, Desolvation energy, Restraints violation energy, Buried Surface Area, and Z-Score are given above. The cluster 1 is the best possible interaction complex for CAR and HAdV3 L5 protein with HADDOCK score of -78.2 +/- 4.0 and Z-score of -1.3 selected for the further analysis.

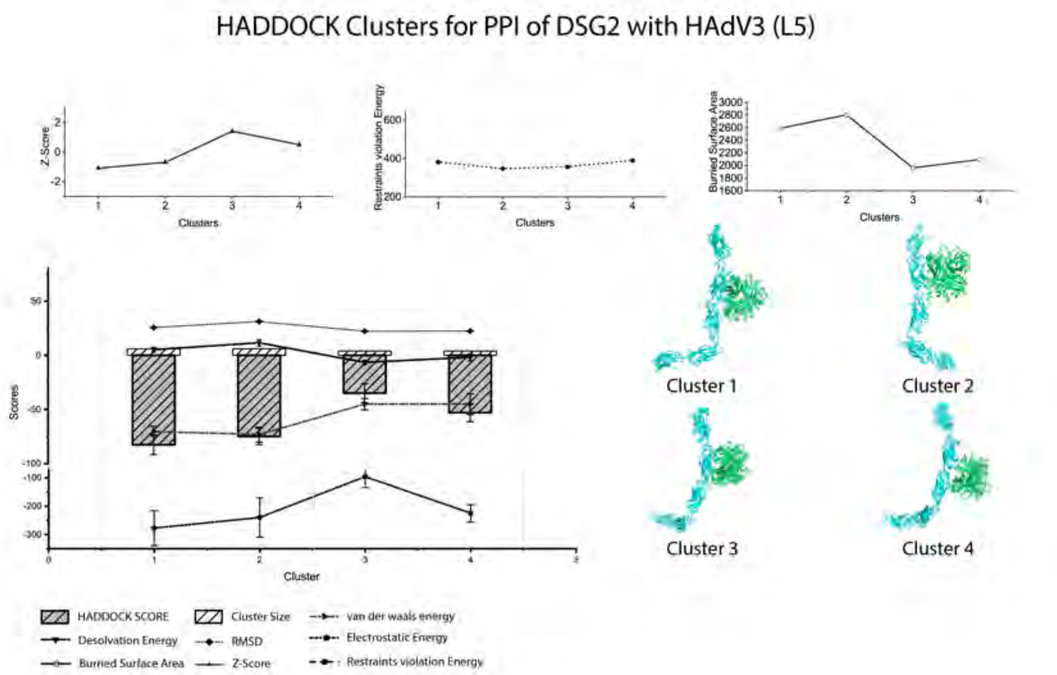

Figure (S24) **Protein-protein interaction complex of HAdV3 L5 protein with DSG2 receptor:** HADDOCK run generate 4 clusters, superimposed structures of all clusters and plots of HADDOCK scores, Cluster size, RMSD, Van der Waals energy, Electrostatic energy, Desolvation energy, Restraints violation energy, Buried Surface Area, and Z-Score are given above. The cluster 1 is the best possible interaction complex for DSG2 and HAdV3 L5 protein with HADDOCK score of -82.4 +/- 9.2 and Z-score of -1.1 selected for the further analysis.

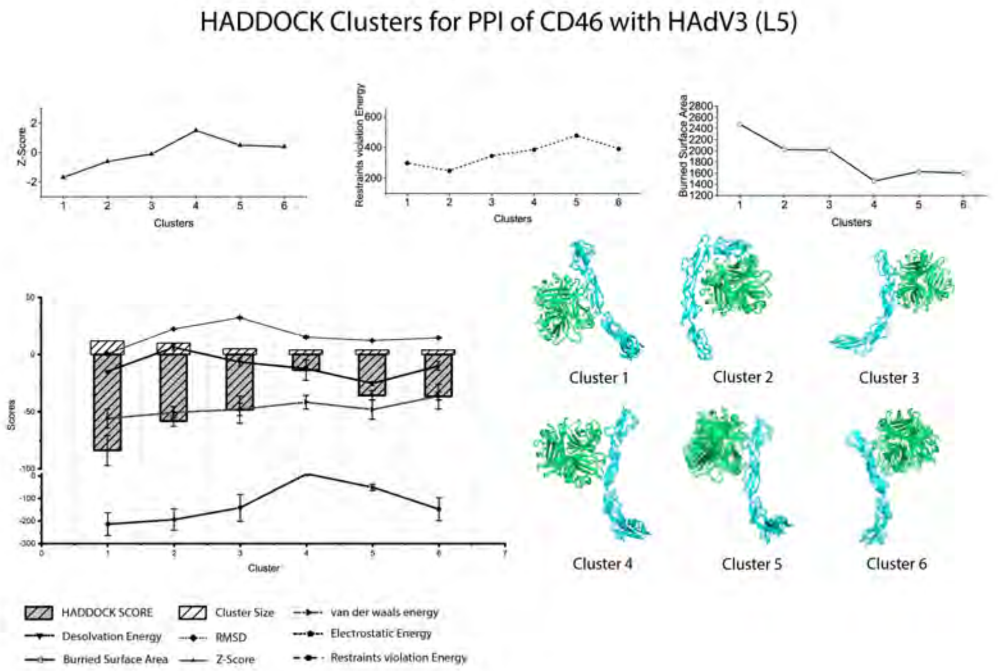

Figure (S25) **Protein-protein interaction complex of HAdV3 L5 protein with CD46 receptor:** HADDOCK run generate 6 clusters, superimposed structures of all clusters and plots of HADDOCK scores, Cluster size, RMSD, Van der Waals energy, Electrostatic energy, Desolvation energy, Restraints violation energy, Buried Surface Area, and Z-Score are given above. The cluster 1 is the best possible interaction complex for CD46 and HAdV3 L5 protein with HADDOCK score of -83.8 +/- 13.2 and Z-score of -1.7 selected for the further analysis.

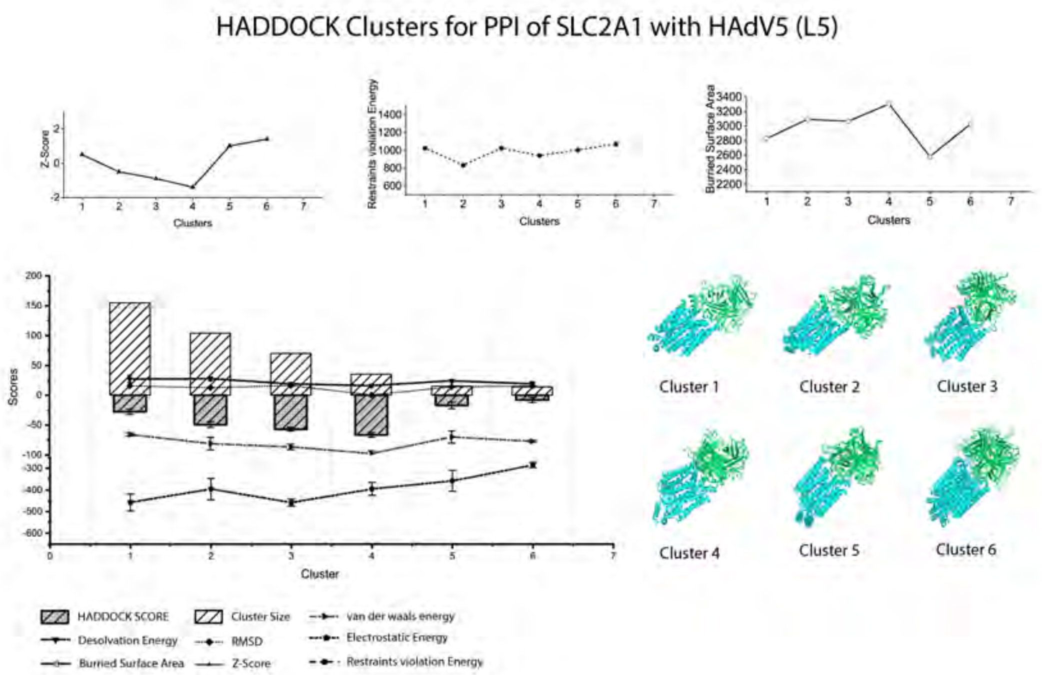

Figure (S26) **Protein-protein interaction complex of HAdV5 L5 protein with SLC2A1 receptor:** HADDOCK run generate 6 clusters, superimposed structures of all clusters and plots of HADDOCK scores, Cluster size, RMSD, Van der Waals energy, Electrostatic energy, Desolvation energy, Restraints violation energy, Buried Surface Area, and Z-Score are given above. The cluster 4 is the top interaction complex with HADDOCK score of -66.3 +/- 3.8 and lowest Z-score of -1.4 selected for the further analysis.

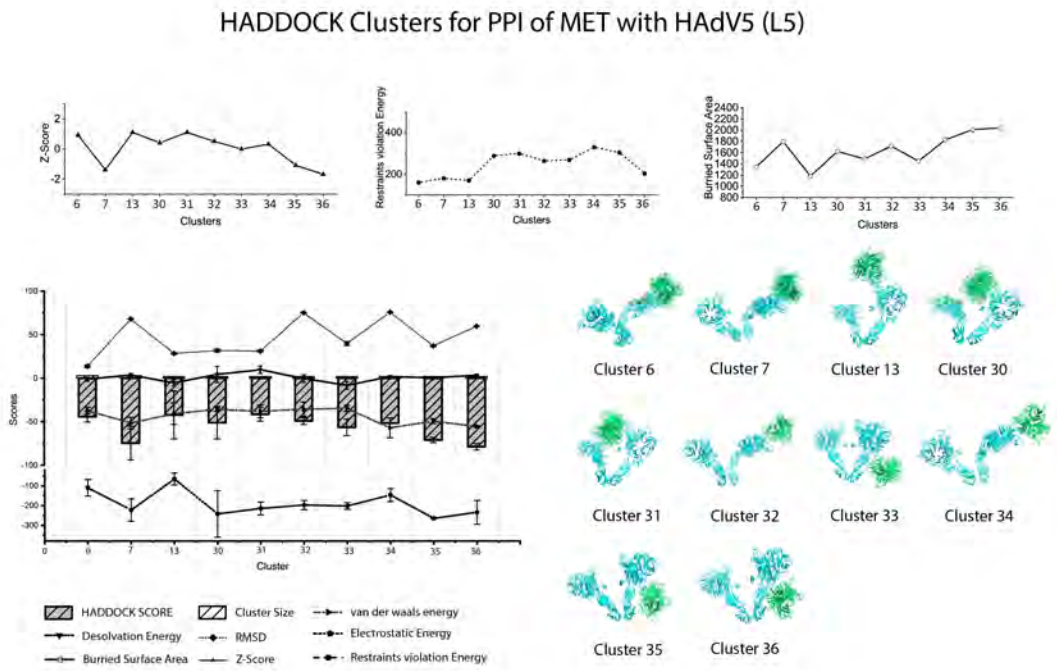

Figure (S27) **Protein-protein interaction complex of HAdV5 L5 protein with MET receptor:** HADDOCK run generate 36 clusters, superimposed structures of top 10 clusters and plots of HADDOCK scores, Cluster size, RMSD, Van der Waals energy, Electrostatic energy, Desolvation energy, Restraints violation energy, Buried Surface Area, and Z-Score are given above. The cluster 36 is the top interaction complex with HADDOCK score of -78.8 +/- 3.7 and lowest Z-score of -1.7 selected for the further analysis.

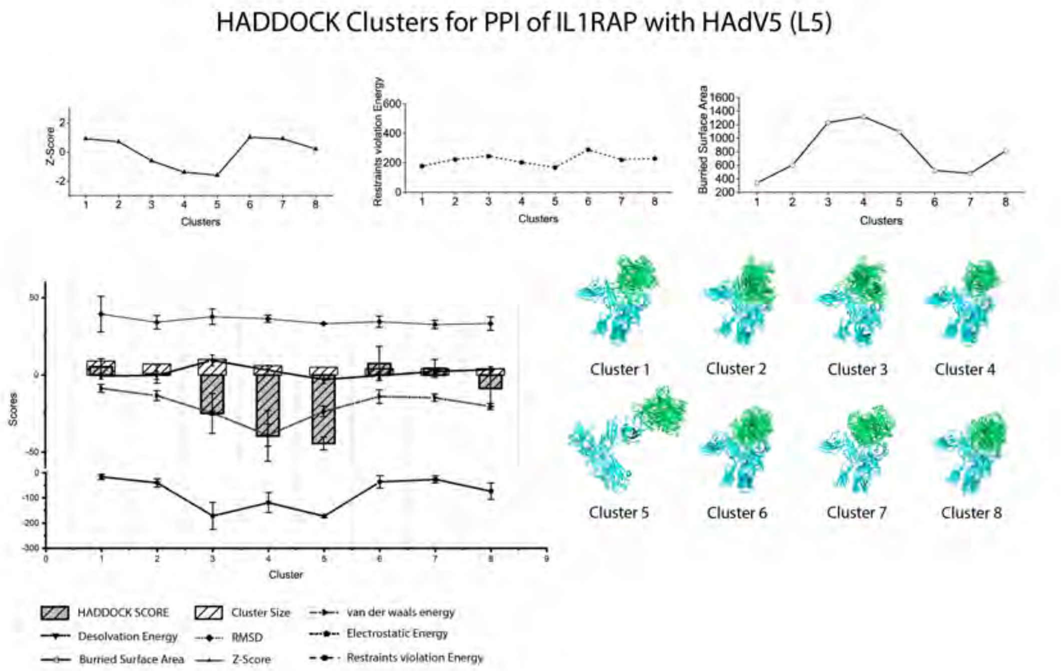

Figure (S28) **Protein-protein interaction complex of HAdV5 L5 protein with IL1RAP receptor:** HADDOCK run generate 8 clusters, superimposed structures of all clusters and plots of HADDOCK scores, Cluster size, RMSD, Van der Waals energy, Electrostatic energy, Desolvation energy, Restraints violation energy, Buried Surface Area, and Z-Score are given above. The cluster 5 is the top interaction complex with HADDOCK score of -44.4 +/- 4.2 and lowest Z-score of -1.6 selected for the further analysis.

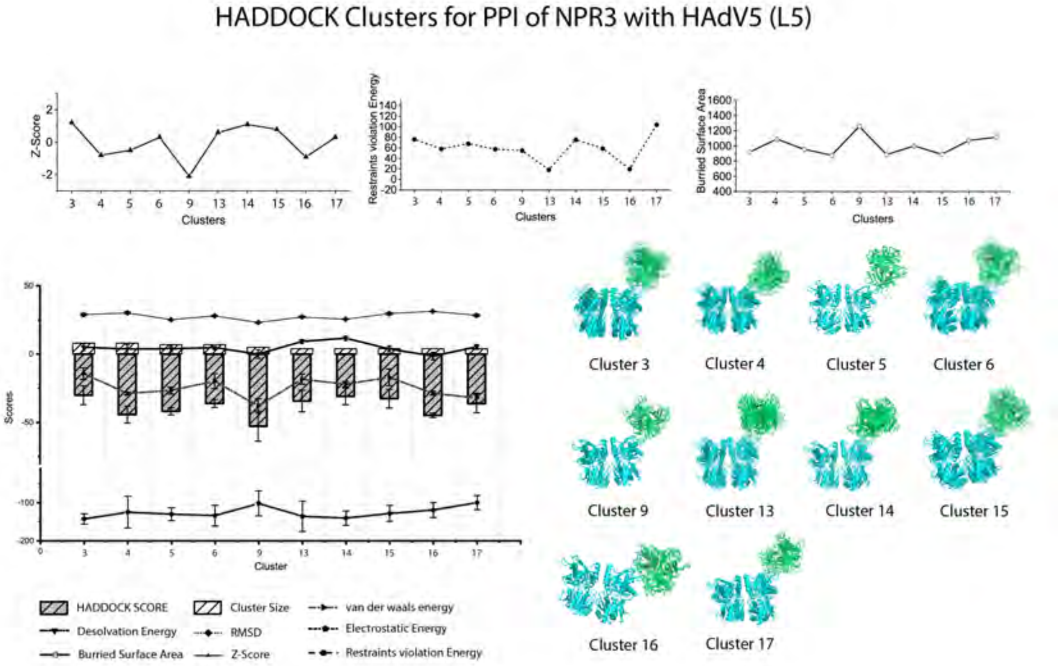

Figure (S29) **Protein-protein interaction complex of HAdV5 L5 protein with NPR3 receptor:** HADDOCK run generate 17 clusters, superimposed structures of top 10 clusters and plots of HADDOCK scores, Cluster size, RMSD, Van der Waals energy, Electrostatic energy, Desolvation energy, Restraints violation energy, Buried Surface Area, and Z-Score are given above. The cluster 9 is the top interaction complex with HADDOCK score of -52.7 +/- 11.0 and lowest Z-score of -2.1 selected for the further analysis.

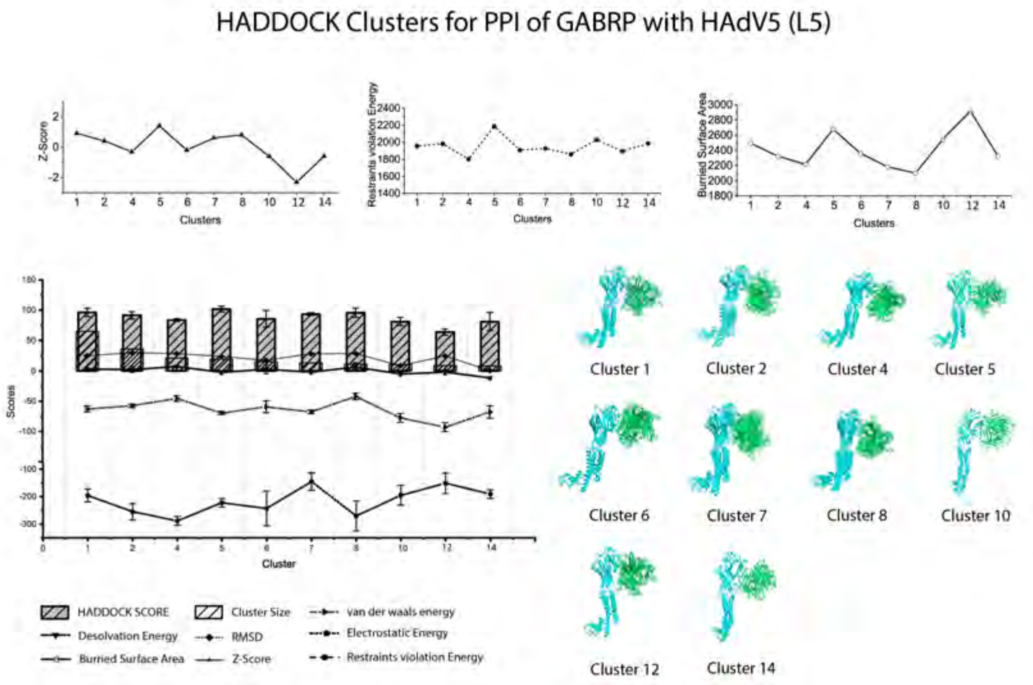

Figure (S30) **Protein-protein interaction complex of HAdV5 L5 protein with GABRP receptor:** HADDOCK run generate 22 clusters, superimposed structures of top 10 clusters and plots of HADDOCK scores, Cluster size, RMSD, Van der Waals energy, Electrostatic energy, Desolvation energy, Restraints violation energy, Buried Surface Area, and Z-Score are given above. The cluster 12 is the top interaction complex with HADDOCK score of 63.8 +/- 5.4 and lowest Z-score of -2.3 selected for the further analysis.

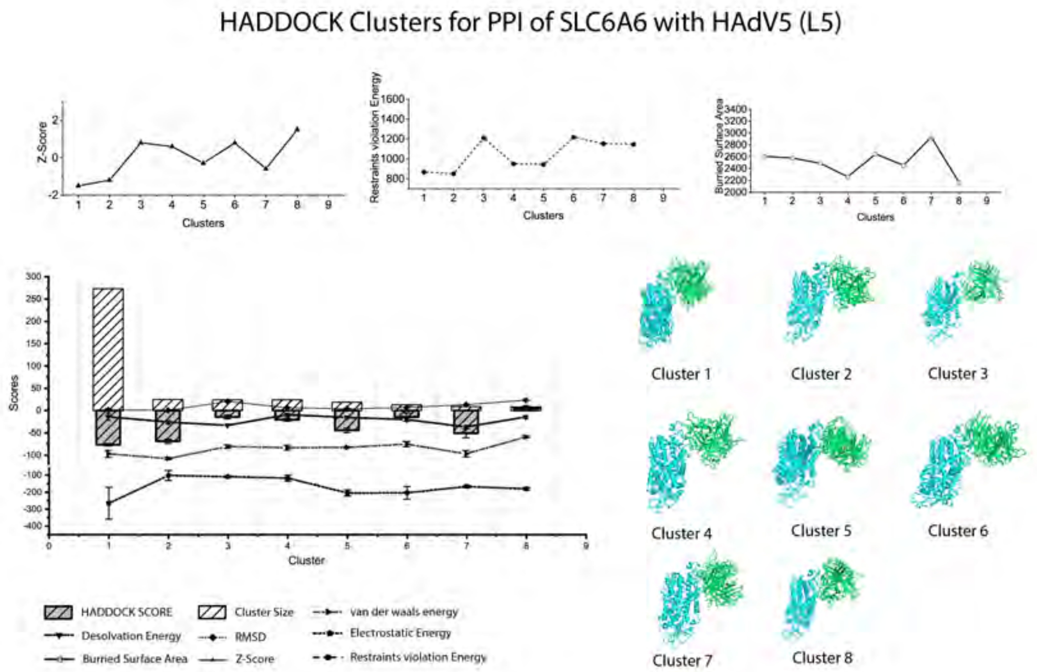

Figure (S31) **Protein-protein interaction complex of HAdV5 L5 protein with SLC6A6 receptor:** HADDOCK run generate 8 clusters, superimposed structures of all clusters and plots of HADDOCK scores, Cluster size, RMSD, Van der Waals energy, Electrostatic energy, Desolvation energy, Restraints violation energy, Buried Surface Area, and Z-Score are given above. The cluster 1 is the top interaction complex with HADDOCK score of -76.9 +/- 2.7 and Z-score of -1.5 selected for the further analysis.

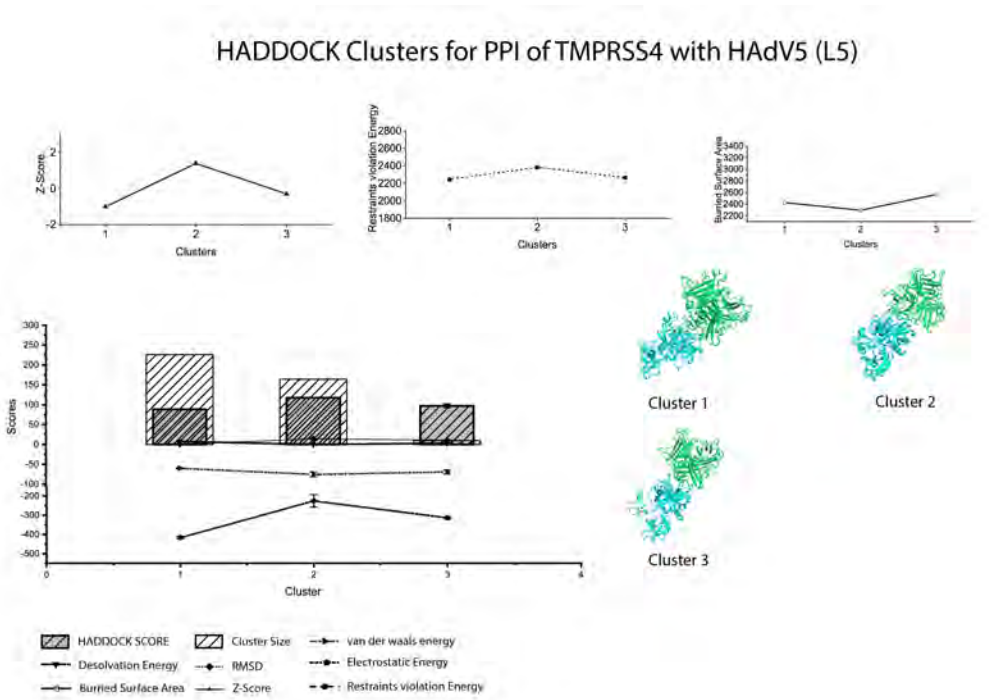

Figure (S32) **Protein-protein interaction complex of HAdV5 L5 protein with TMPRSS4 receptor:** HADDOCK run generate 3 clusters, superimposed structures of all clusters and plots of HADDOCK scores, Cluster size, RMSD, Van der Waals energy, Electrostatic energy, Desolvation energy, Restraints violation energy, Buried Surface Area, and Z-Score are given above. The cluster 1 is the top interaction complex with HADDOCK score of 88.5 +/- 0.9 and Z-score of -1.0 selected for the further analysis.

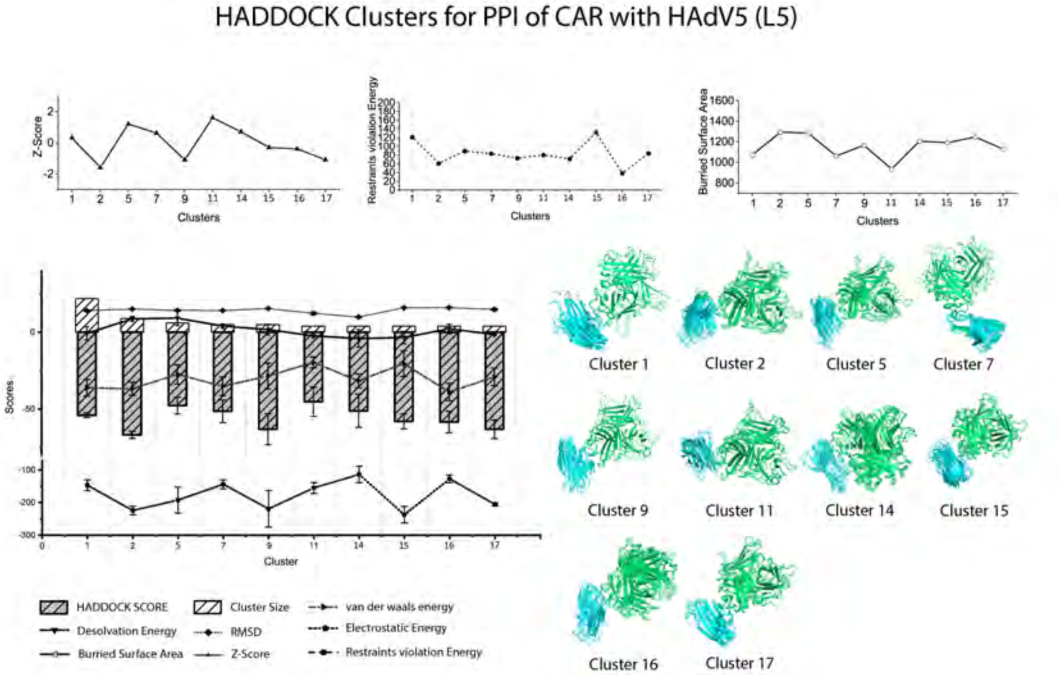

Figure (S33) **Protein-protein interaction complex of HAdV5 L5 protein with CAR receptor:** HADDOCK run generate 17 clusters, superimposed structures of top 10 clusters and plots of HADDOCK scores, Cluster size, RMSD, Van der Waals energy, Electrostatic energy, Desolvation energy, Restraints violation energy, Buried Surface Area, and Z-Score are given above. The cluster 2 is the best possible interaction complex for CAR and HAdV5 L5 protein with HADDOCK score of -66.9 +/- 2.3 and Z-score of -1.6 selected for the further analysis.

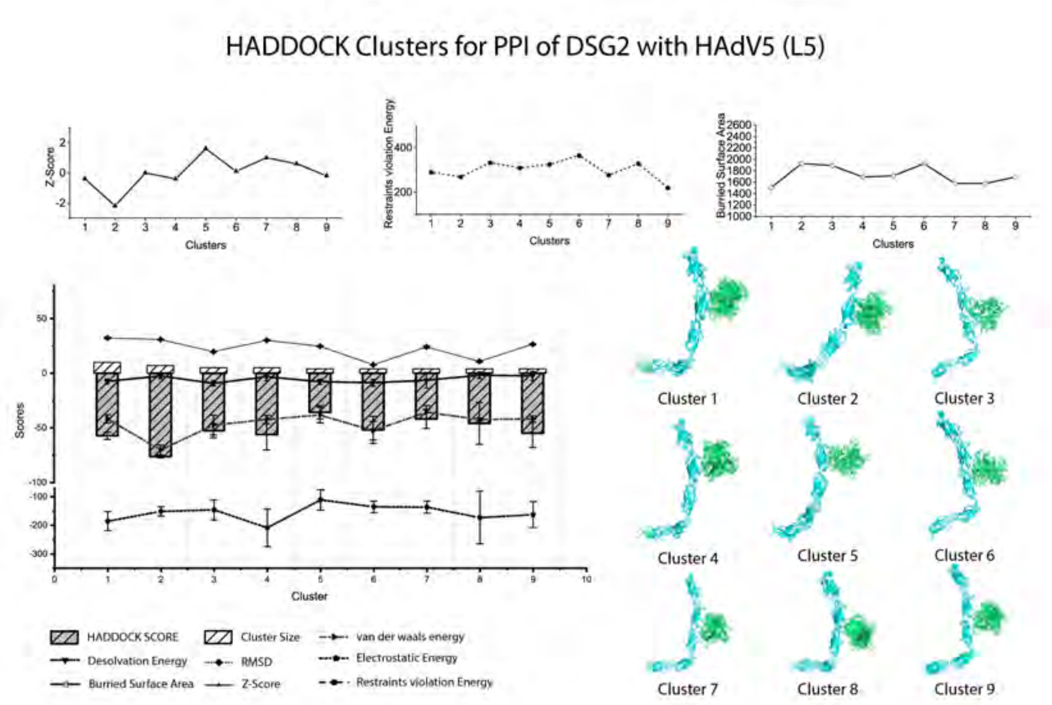

Figure (S34) **Protein-protein interaction complex of HAdV5 L5 protein with DSG2 receptor:** HADDOCK run generate 9 clusters, superimposed structures of all clusters and plots of HADDOCK scores, Cluster size, RMSD, Van der Waals energy, Electrostatic energy, Desolvation energy, Restraints violation energy, Buried Surface Area, and Z-Score are given above. The cluster 2 is the best possible interaction complex for DSG2 and HAdV5 L5 protein with HADDOCK score of -76.2 +/- 1.3 and Z-score of -2.2 selected for the further analysis.

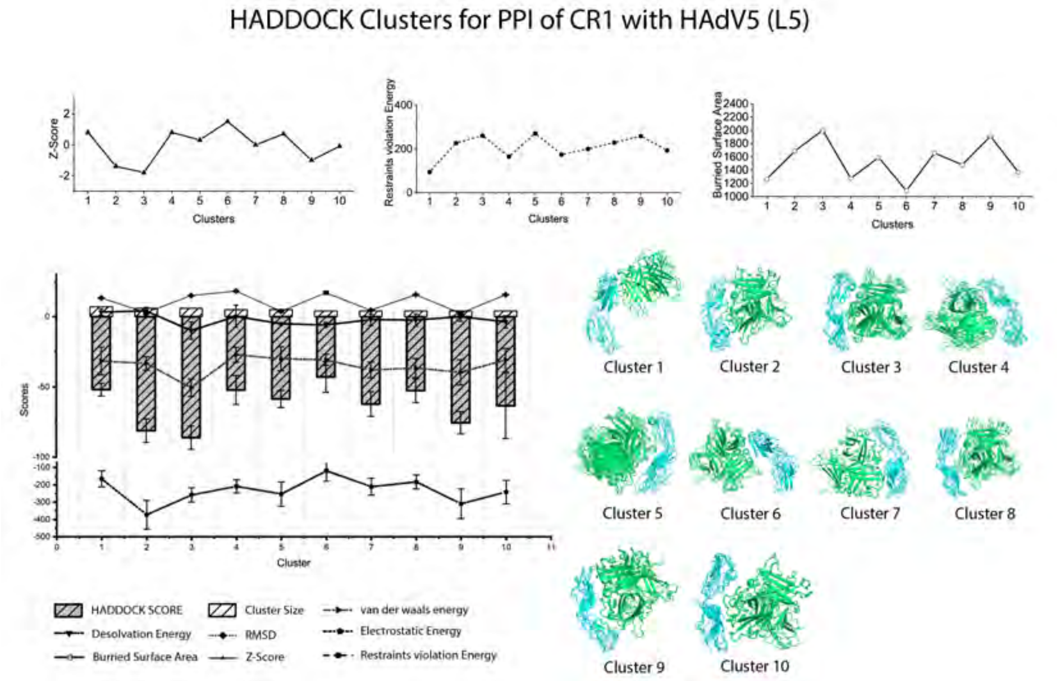

Figure (S35) **Protein-protein interaction complex of HAdV5 L5 protein with CR1 receptor sushi domain 1 and 2:** HADDOCK run generate 10 clusters, superimposed structures of all clusters and plots of HADDOCK scores, Cluster size, RMSD, Van der Waals energy, Electrostatic energy, Desolvation energy, Restraints violation energy, Buried Surface Area, and Z-Score are given above. The cluster 3 is the best possible interaction complex for CR1 and HAdV5 L5 protein with HADDOCK score of -86.1 +/- 8.3 and Z-score of -1.8 selected for the further analysis.

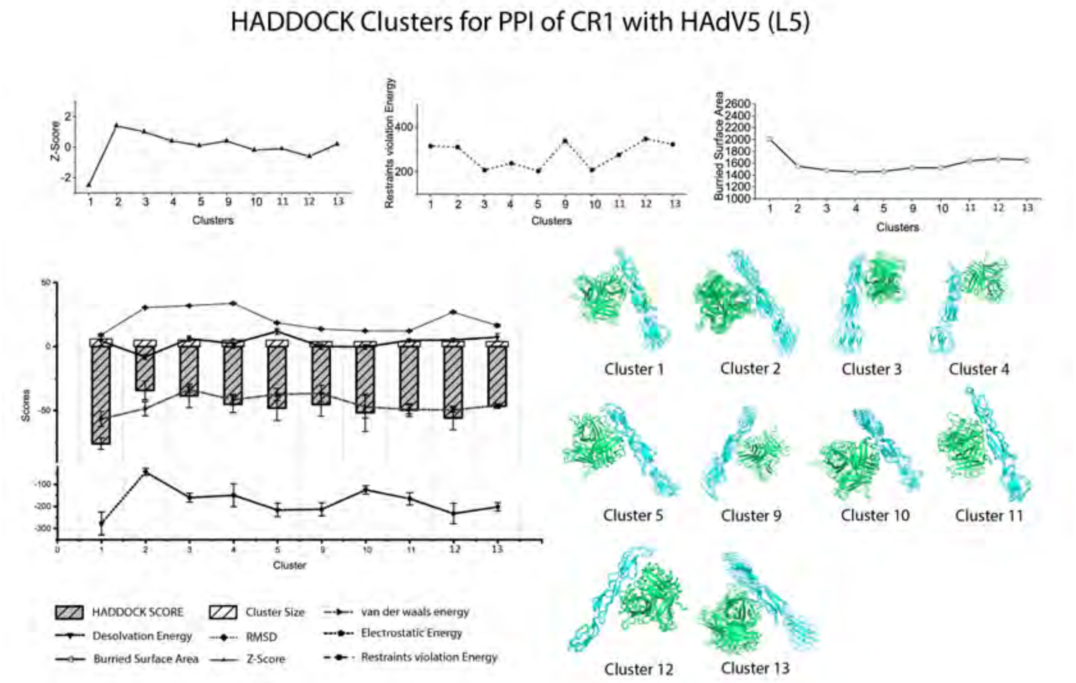

Figure (S36) **Protein-protein interaction complex of HAdV5 L5 protein with CR1 receptor sushi domain 15, 16 and 17:** HADDOCK run generate 13 clusters, superimposed structures of top 10 clusters and plots of HADDOCK scores, Cluster size, RMSD, Van der Waals energy, Electrostatic energy, Desolvation energy, Restraints violation energy, Buried Surface Area, and Z-Score are given above. The cluster 1 is the best possible interaction complex for CR1 and HAdV5 L5 protein with HADDOCK score of -75.9 +/- 4.4 and Z-score of -2.5 selected for the further analysis.

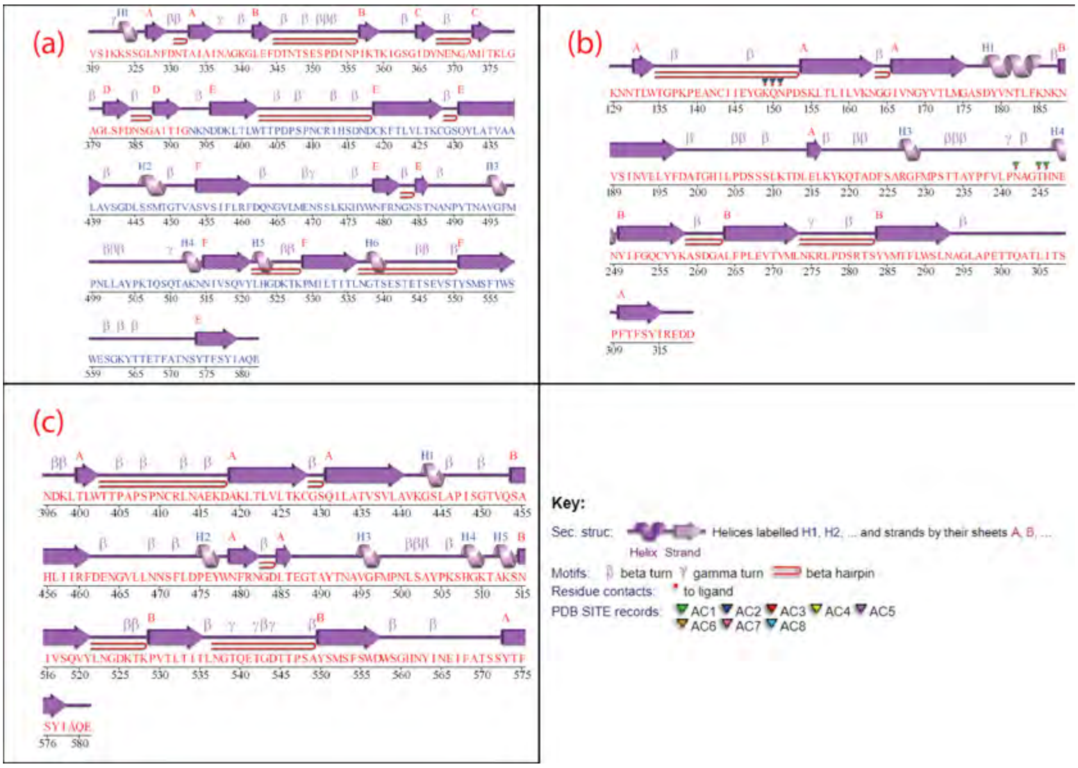

Figure (S37) **The PDB sum secondary structure of human adenovirus L5 proteins:** (a) HAdV2 (b) HAdV3 (c) HAdV5

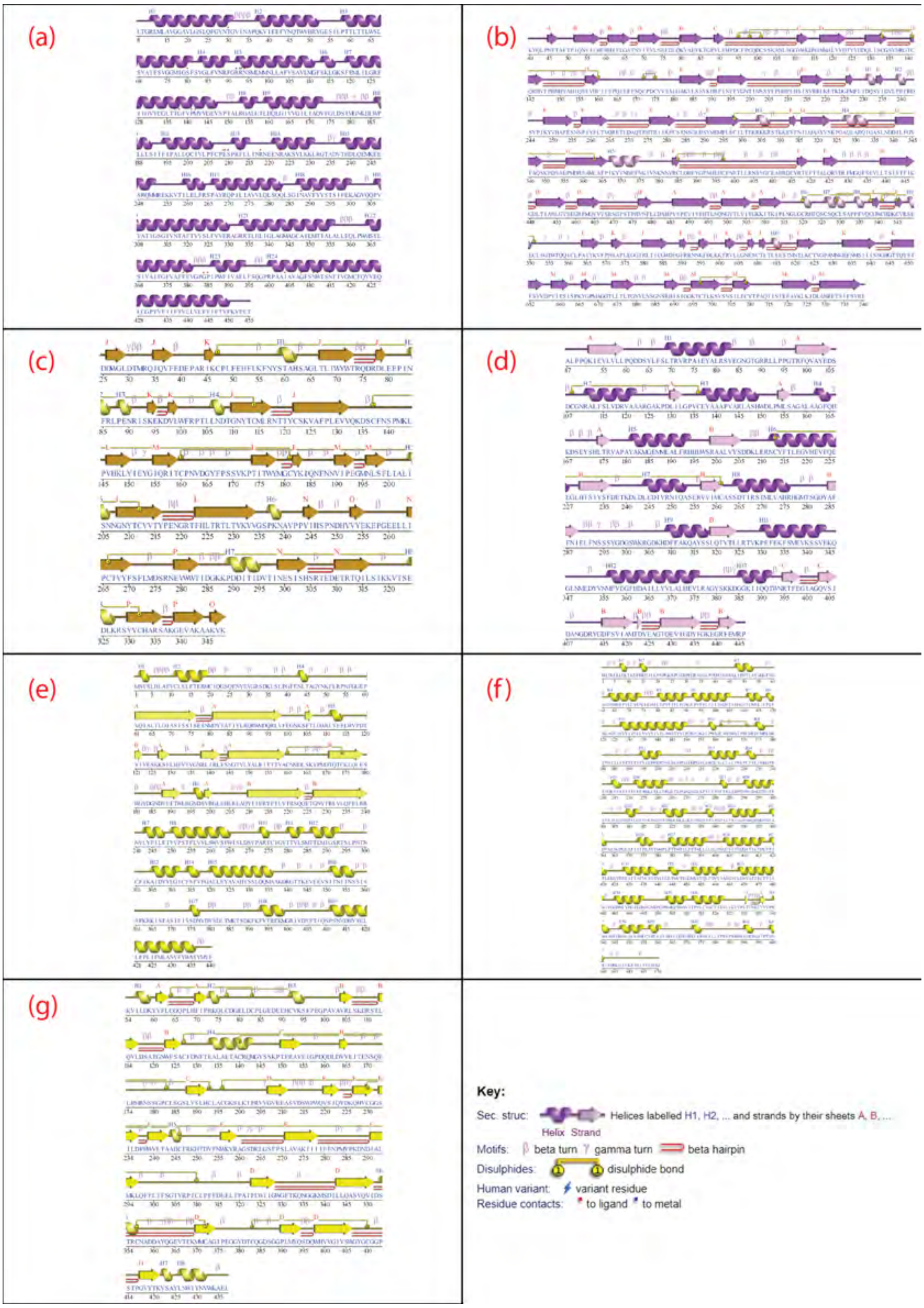

Figure (S38) **The PDB sum secondary structure of human pancreatic cancer receptors:** (a) SLC2A1 (b) MET (c) IL1RAP (d) NPR3 (e) GABRP (f) SLC6A6 (g) TMPRSS4

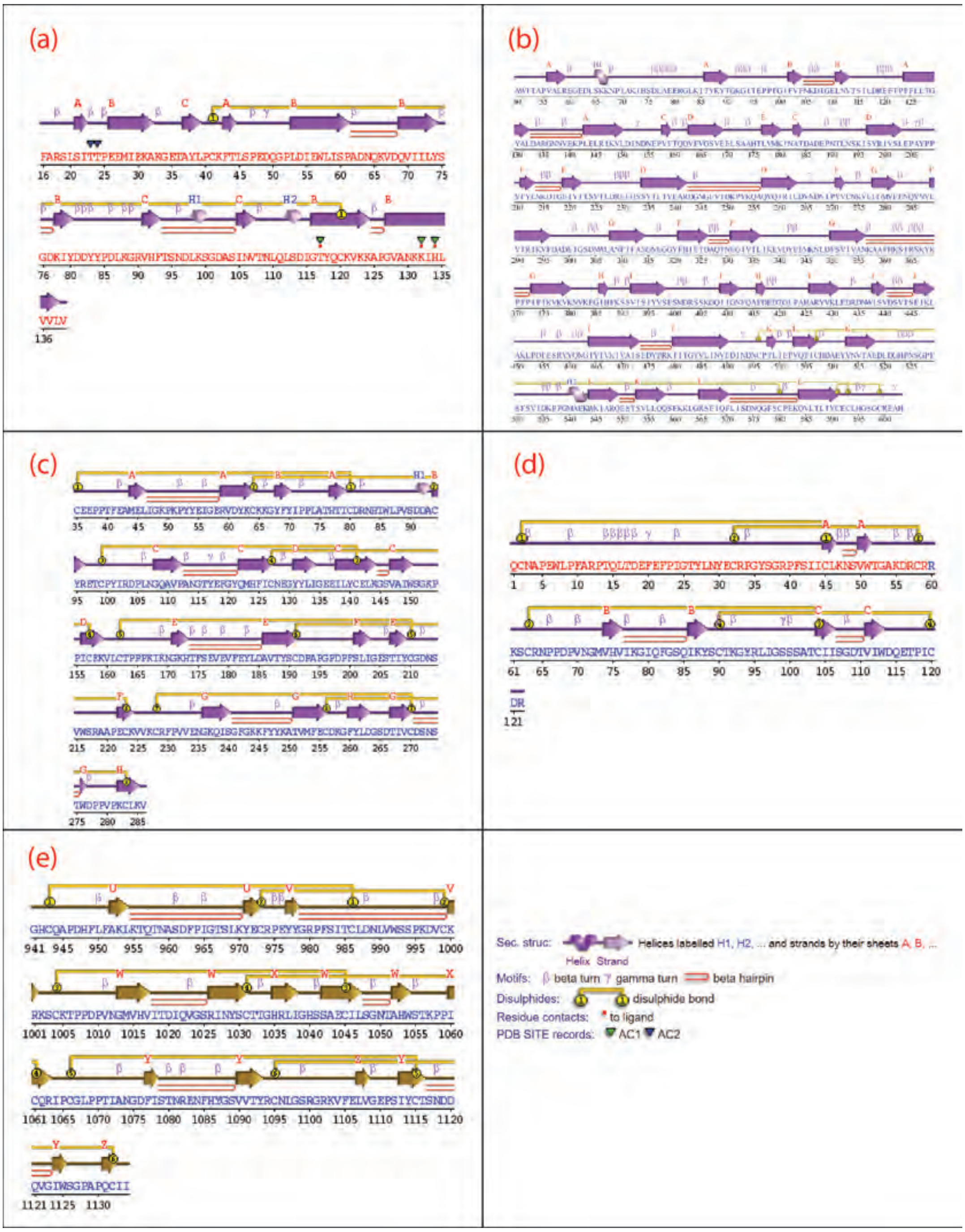

Figure (S39) **The PDB sum secondary structure of human receptors positive control:** (a) CAR (b) DSG2 (c) CD46 (d) CR1 receptor sushi domain 1 and 2 (e) CR1 receptor sushi domain 15, 16, and 17
